# Electronic isomerism in a heterometallic nickel–iron–sulfur cluster models substrate binding and cyanide inhibition of carbon monoxide dehydrogenase†

**DOI:** 10.1039/d4sc00023d

**Published:** 2024-03-27

**Authors:** Luke C. Lewis, José A. Sanabria-Gracia, Yuri Lee, Adam J. Jenkins, Hannah S. Shafaat

**Affiliations:** a Department of Chemistry and Biochemistry, The Ohio State University Columbus OH 43210 USA; b Department of Chemistry and Biochemistry, University of California, Los Angeles Los Angeles CA 90095 USA shafaat@ucla.edu

## Abstract

The nickel–iron carbon monoxide dehydrogenase (CODH) enzyme uses a heterometallic nickel–iron–sulfur ([NiFe_4_S_4_]) cluster to catalyze the reversible interconversion of carbon dioxide (CO_2_) and carbon monoxide (CO). These reactions are essential for maintaining the global carbon cycle and offer a route towards sustainable greenhouse gas conversion but have not been successfully replicated in synthetic models, in part due to a poor understanding of the natural system. Though the general protein architecture of CODH is known, the electronic structure of the active site is not well-understood, and the mechanism of catalysis remains unresolved. To better understand the CODH enzyme, we have developed a protein-based model containing a heterometallic [NiFe_3_S_4_] cluster in the *Pyrococcus furiosus* (*Pf*) ferredoxin (Fd). This model binds small molecules such as carbon monoxide and cyanide, analogous to CODH. Multiple redox- and ligand-bound states of [NiFe_3_S_4_] Fd (NiFd) have been investigated using a suite of spectroscopic techniques, including resonance Raman, Ni and Fe K-edge X-ray absorption spectroscopy, and electron paramagnetic resonance, to resolve charge and spin delocalization across the cluster, site-specific electron density, and ligand activation. The facile movement of charge through the cluster highlights the fluidity of electron density within iron–sulfur clusters and suggests an electronic basis by which CN^−^ inhibits the native system while the CO-bound state continues to elude isolation in CODH. The detailed characterization of isolable states that are accessible in our CODH model system provides valuable insight into unresolved enzymatic intermediates and offers design principles towards developing functional mimics of CODH.

## Abstract 2

A protein-based model of carbon monoxide dehydrogenase displays distinct X-ray absorption, EPR, and vibrational signatures upon CO and CN^−^ binding that support ligand-dependent electronic rearrangement throughout the nickel–iron–sulfur core.

## Graphical abstract

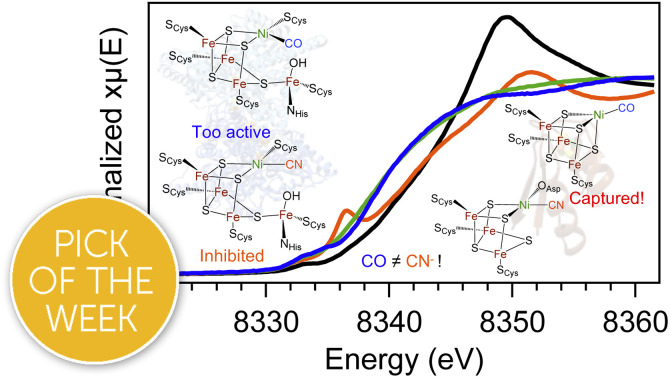

## Introduction

Biological iron–sulfur (Fe–S) clusters perform a wide array of chemical functions across all kingdoms of life,^1^ from electron transfer to complex chemical reactions.^2–6^ A subset of iron–sulfur clusters employ a heterometallic (MFe_*x*_S_*y*_) structure (M = Mo, V, Ni) to perform challenging biological transformations such as nitrogen fixation and carbon dioxide activation, driving great interest in their study.^7–9^ The carbon monoxide dehydrogenase (CODH) enzyme uses a cuboidal [NiFe_4_S_4_] cluster, known as the C-cluster,^10^ to catalyze the reversible reduction of carbon dioxide (CO_2_) to carbon monoxide (CO) at ambient temperatures and pressures.^11^ The enzyme performs this difficult interconversion with high turnover rates, low overpotential, and perfect selectivity,^7,12–14^ a set of characteristics that has yet to be replicated in any synthetic catalyst.^15,16^ Thus, understanding the enzymatic mechanism and elucidating key intermediates in the catalytic cycle has become an active area of research, with the overarching goals of applying these principles to the design of future synthetic catalysts for global carbon cycling and conversion.

**Fig. 1:**
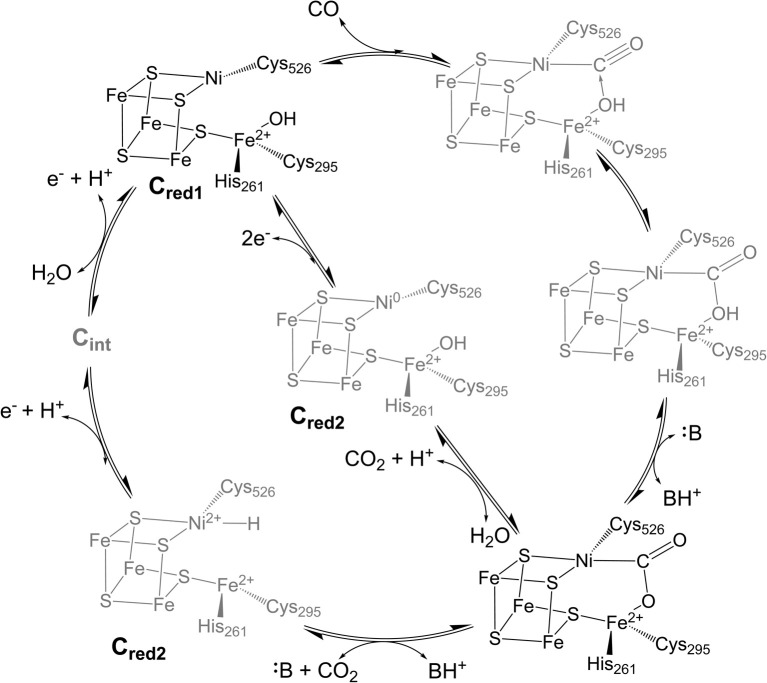
Proposed catalytic cycle for CO oxidation and CO_2_ reduction at the C-cluster of CODH showing two different hypothesized structures for C_red2_. Amino acid numbering is from CODH II_Ch_. Structures in gray indicate intermediate states that are not well-characterized.

However, CODH is a large and complex homodimeric protein with two additional [Fe_4_S_4_] clusters per subunit required for electron transfer, complicating attempts to characterize intermediates within the catalytic cycle.^17,18^ At this point in time, few CODH states can be considered well-understood (Fig. 1). The C_red1_, C_red2,_ and “C_red2-CO_2__” states have been isolated and characterized spectroscopically and crystallographically,^10,19^ while the C_red1_ and C_red2_ states have been further analyzed using electrochemical techniques.^13,20^ However, the electronic structure of the C_red2_ state is still unknown, as two isoelectronic species have been proposed (Fig. 1).^7,21^ An EPR study indicating the presence of an EPR-silent state (C_int_) between C_red1_ and C_red2_ has further convoluted the proposed mechanism, indicating two potential pathways to the “C_red2-CO_2__” state.^22^ Moreover, despite its importance, no CO-bound state of CODH has ever been isolated or observed. While an early structure of CO-exposed *Methanosarcina barkeri* CODH found coordination of a diatomic ligand at the nickel site, the identity of this ligand has since been attributed to a bound formyl species.^23^ Considering the high reactivity of CODH towards CO oxidation,^12,20^ understanding this intermediate will provide valuable insight into the electronic basis for catalysis.

One strategy to elucidate structure–function relationships of complex metalloenzymes includes the development and study of model systems that replicate key structural motifs.^24^ The most popular method for modelling the active sites of enzymes has been to design complexes that mimic the primary coordination sphere within a synthetic ligand scaffold.^7,25–28^ While modelling the heterometallic iron–sulfur cubane of CODH has been achieved in select systems,^29–33^ and some of these model compounds also bind cyanide (CN^−^), a known inhibitor of CODH,^29^ none of the aforementioned structural models have demonstrated the propensity to bind CO_2_ or CO.^7,34^ Furthermore, these synthetic models are generated in aprotic organic solvents and typically utilize π-accepting ligands such as phosphines, which have significantly different bonding characteristics than the π-donating cysteine thiolate ligands of the native system. The development of protein-based models addresses many of these key differences, replicating exact geometric and electronic structural motifs while in some cases also mimicking the reactivity of the native system.^35–39^ Inspired by other model systems, we sought to develop a protein-based model of the C-cluster within a ferredoxin scaffold. By reproducing the iron–sulfur-cluster moeity, we hypothesized that we could access reactivity analogous to CODH and use spectroscopy to identify key electronic motifs promoting reactivity.

Our initial work has shown that incorporation of Ni^II^ into the site-differentiated iron–sulfur cluster of the *Pyrococcus furiosus* (*Pf*) ferredoxin (Fd) produced a stable, heterometallic [NiFe_3_S_4_]^2+^ cluster in Fd (NiFd_ox_) capable of rapid, reversible electron transfer.^40,41^ The reduced [NiFe_3_S_4_]^+^ Fd (NiFd_red_) displayed the ability to bind both CN^−^ and CO, as evidenced by EPR spectroscopy. The NiFd–CO species serves as the first model for the CO-bound state of CODH. Herein, we use an array of complementary spectroscopic techniques to resolve the electronic structures of the NiFd_red_, NiFd_ox_, NiFd–CN, and NiFd–CO species, including variable-temperature electron paramagnetic resonance (EPR) spectroscopy, resonance Raman (rR) spectroscopy, X-ray absorption spectroscopy (XAS), and computational modelling. This study reveals distinct changes in the system upon reduction and binding of CO and CN^−^, suggesting a rearrangement of electron density within the cuboidal heterometallic iron–sulfur cluster depending on the identity of the ligand. The relationship between the NiFd–CO species and the “untrappable” C_red1-CO_ state in CODH is discussed, along with the role of electronic isomerization in driving inhibition of CODH activity by CN^−^. As the C_red1-CO_ state has yet to be observed in the native system, the in-depth characterization of the NiFd–CO species presented here provides valuable insight into the spin state, geometry, and complex electronic structure of this elusive C_red1-CO_ intermediate. Given the high catalytic rates and full reversibility of native CODH towards CO oxidation and CO_2_ reduction,^12,20^ elucidating the structure of this essential species will facilitate the development of functional models that reproduce this reactivity, with long-term implications in environmental remediation and energy storage.

## Results

### Isotopic labelling indicates spin is delocalized across Ni and Fe

**Fig. 2:**
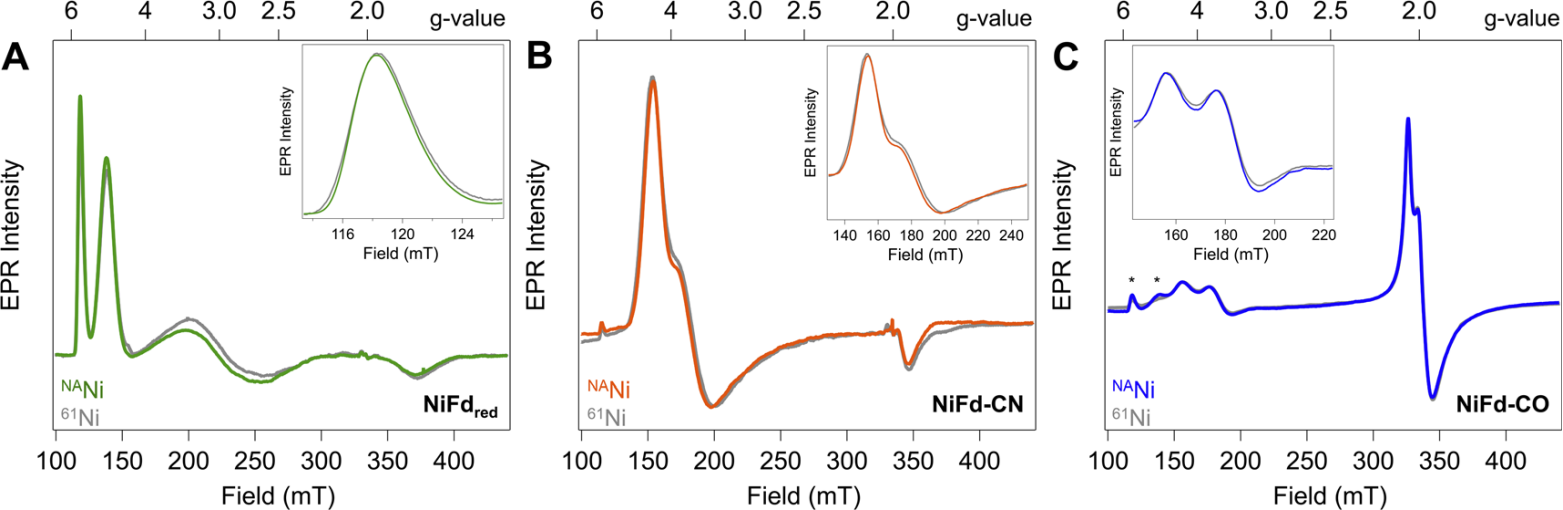
CW X-band EPR spectra (*ν* = 9.37 GHz, *P*_μw_ = 20 mW, *T* = 8.0 K) of natural abundance (colored traces) and ^61^Ni-isotopically labelled (gray traces) samples of (A) NiFd_red_, (B) NiFd–CN, and (C) NiFd–CO. (Insets) Zoomed in views of low-field turning points highlight ^61^Ni-induced broadening. * indicates residual NiFd_red_ that remains after exposure to CO.

Reduction of the EPR-silent nickel-incorporated NiFd_ox_ species to the NiFd_red_ state gives a characteristic X-band EPR spectrum spanning ∼300 mT, with sharp peaks at *g*_app_ = 5.7 and *g*_app_ = 4.3 as well as broad peaks around *g*_app_ ∼ 2.7 and *g*_app_ ∼ 1.9 that are consistent with the formation of a single *S* = 3/2 species. This species had previously been simulated with spin Hamiltonian parameters of *g*_iso_ = 2.0 and |E/D| = 0.16 (Fig. 2A). Introduction of CN^−^ results in quantitative conversion to a new *S* = 3/2 species, with peaks at *g*_app_ = 4.35, 3.9, 1.92 that had been simulated with spin Hamiltonian parameters of *g*_iso_ = 2.0 and |E/D| = 0.07 (Fig. 2B). A more complicated spectrum is obtained upon exposure to CO, as conversion to the new species is not quantitative. We attribute the residual NiFd_red_ to the limited solubility of CO in aqueous solutions and weak binding affinity of NiFd_red_ towards CO. However, the spectra are dominated by two new apparent sets of signals- one set starting around *g*_app_ ∼ 4 that would be consistent with an *S* = 3/2 species, and one set around *g*_app_ ∼ 2 that would be consistent with an *S* = 1/2 species (Fig. 2C). These signals were assumed to derive from a spin-coupling scheme within the cluster that involved all 4 metal centers with local high spin, analogous to conventional iron sulfur clusters. This intracluster coupling was implied to change upon ligand binding, giving rise to distinct spectra.

To test this hypothesis and obtain element-specific information on the spin projection across each center in the EPR-active NiFd_red_, NiFd–CN, and NiFd–CO states, we incorporated ^61^Ni (*I* = 3/2) and ^57^Fe (*I* = 1/2) into the cluster. As previously observed for homometallic iron–sulfur clusters and other heterometallic clusters, ^57^Fe labeling resulted in global broadening of all species, consistent with a significant degree of spin delocalized across the Fe centers (Fig. S1†).^42,43^ The nickel contributions were more distinct across the NiFd species. In all cases, the broad linewidth precluded observation of well-defined hyperfine peaks from the *I* = 3/2 nucleus, which is typical of high-spin systems.^40,44^ A modest, 0.4 mT line broadening was observed for the *g*_app_ = 5.7 feature of NiFd_red_ upon ^61^Ni incorporation (Fig. 2A), in good agreement with previously reported results.^40^ Similarly, upon isotopic labelling, the NiFd–CN and NiFd–CO exhibit 0.7 and 0.4 mT line broadening at the *g*_app_ = 4.35 and 3.65 features, respectively (Fig. 2B and C). Notably, there is no substantial broadening (<0.1 mT) of the feature at *g*_app_ = 2.05 for the NiFd–CO species (Fig. S2†). The measurable but small line broadening observed for all species indicates that spin density is distributed across the iron and nickel centers in the cluster, rather than being localized on a single ion, and highlights the integral role of nickel in the overall spin-coupling scheme of the cluster. Work is ongoing to obtain increased resolution of the electronic hyperfine coupling to ^57^Fe and ^61^Ni nuclei using variable-field Mössbauer and pulsed, high-frequency EPR techniques but is beyond the scope of this work.

### Variable temperature CW-EPR suggests the presence of a single NiFd–CO species

**Fig. 3:**
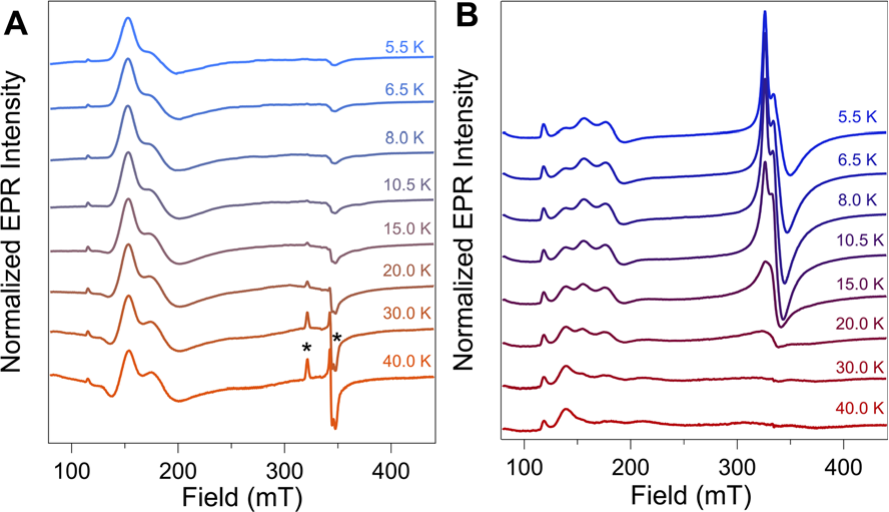
CW EPR spectra (*ν* = 9.37 GHz, *P*_μw_ = 20 mW) of (A) NiFd–CN and (B) NiFd–CO at the indicated temperatures. Spectra were normalized to temperature using a standard Curie dependence [*I* × *T*]. * denotes small (<5%) amount of contaminating *S* = 1/2 [Fe_4_S_4_]–CN Fd.

To further evaluate the origin of the distinct EPR signals at *g*_app_ ∼ 4 and *g*_app_ ∼ 2 in NiFd–CO, variable temperature (VT) CW-EPR spectra were measured and compared to samples of NiFd–CN (Fig. 3). The VT-EPR lineshapes of NiFd–CN do not change significantly from 5.5 K to 40 K, suggestive of a single *S* = 3/2 species (Fig. 3A). On the other hand, the VT-EPR lineshapes of NiFd–CO display unusual behavior. The feature centered at *g*_app_ = 2.05 broadens substantially and decreases in intensity from 5.5 to 15.0 K, counter to what is typically observed in conventional mononuclear or spin-coupled *S* = 1/2 systems (Fig. S3–S5†). By 20 K, the signal centered at *g*_app_ = 2.05 is completely gone, while the low-field signal at *g*_app_ ≈ 4 can still be observed. Comparison of the individual signal intensities to the total integrated intensity across the temperature range suggests the *g*_app_ ∼ 2 species converts into the *g*_app_ ∼ 4 species at higher temperatures, rather than arising from independent signals (Fig. S6 and S7†).

The power saturation behavior of the NiFd_red_, NiFd–CN, and NiFd–CO was assessed from 5.5 to 15.0 K to estimate the energetic spacing of excited states. The *P*_1/2_ values of the NiFd–CO features were consistently larger than those found for the other NiFd species, suggestive of faster relaxation rates. Both the NiFd_red_ and NiFd–CN thus appear to be well-isolated *S* = 3/2 spin systems (Fig. S8–S14†), while the NiFd–CO shows much faster relaxation. Collectively, the temperature-dependent lineshapes, Curie-corrected intensities, and relaxation properties suggest the NiFd–CO species possesses an *S* = 1/2 ground spin state with a low-lying *S* = 3/2 excited state.^45,46^

### *ν*_CO_ of NiFd–CO suggests a large degree of ligand activation

In prior work, Fourier transform infrared (FTIR) spectroscopy was used to probe the *ν*_CN_ mode frequency, which was observed at 2050 cm^−1^ and suggested a strong degree of σ donation from the CN^−^ ligand and only weak π back-bonding from nickel.^41^ However, we were unable to observe an IR band for the NiFd–CO sample from 1800 to 2200 cm^−1^ that would be indicative of a bound CO ligand (Fig. S15†). The lack of signal was attributed to the low binding affinity of CO to NiFd_red_ and limited CO solubility in aqueous solutions, which prevents measurements at high protein concentrations. Because we were unable to acquire vibrational information using FTIR spectroscopy, resonance Raman spectroscopy was employed.

**Fig. 4:**
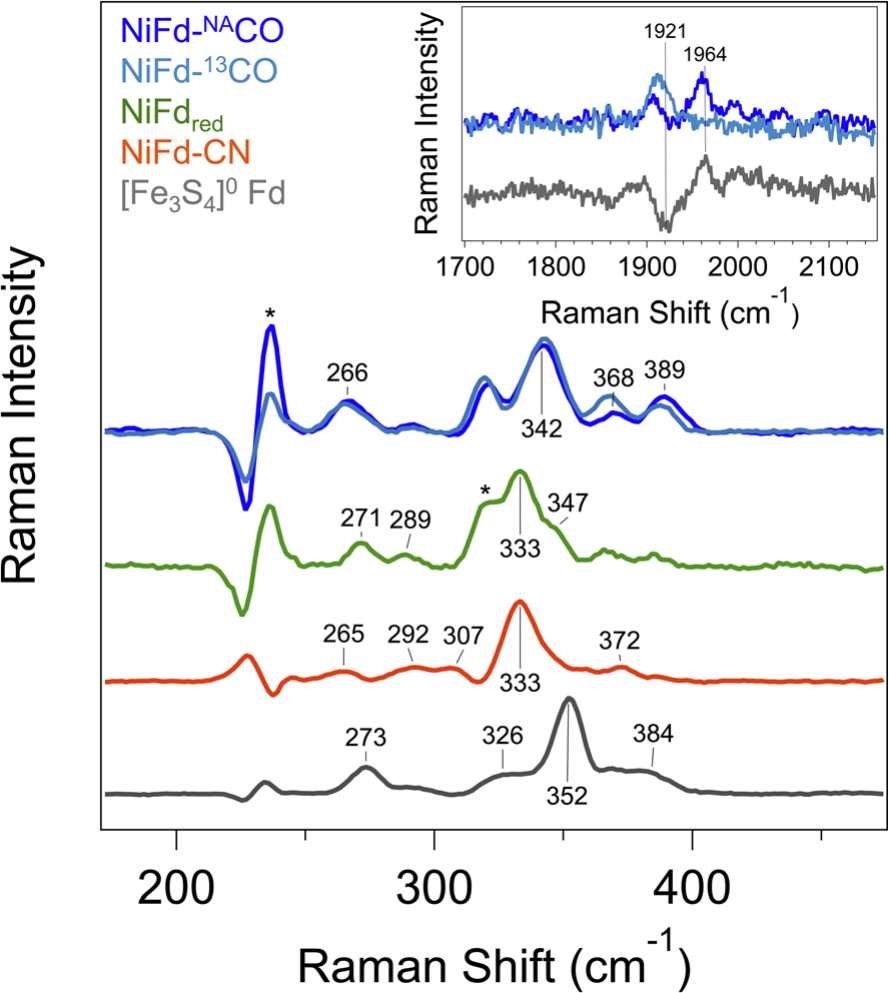
Resonance Raman spectra of [Fe_3_S_4_]^0^ Fd (gray), NiFd_red_ (green), NiFd–CN (orange), and NiFd–CO (blue). Samples were collected at 77 K using an excitation wavelength of 407 nm, *P* = 8 mW. Residual features corresponding to buffer are indicated with an *. Bands arising from buffer, DT, and quartz were subtracted after collection. (Inset) High frequency region of the resonance Raman spectra of NiFd–CO prepared with natural abundance CO (dark blue) and ^13^CO (light blue), shown offset from the difference spectrum (gray). The band at 1906 cm^−1^ is present in both samples and independent of the CO isotope.

In addition to revealing the *ν*_CO_ mode frequency, this technique provides information on structure by resolving the low-frequency cluster vibrational modes. The resonance Raman spectrum of reduced [Fe_3_S_4_]^0^ Fd exhibits a strong vibrational band centered at 352 cm^−1^, which is attributed to the symmetric Fe–S_bridging_ stretching modes (Fig. 4). Weaker vibrational bands can be seen arising from the Fe–S_terminal_ modes.^47^ Upon incorporation of the Ni center into the cluster, the dominant vibrational band shifts to 333 cm^−1^. This bathochromic frequency shift is consistent with incorporation of a fourth metal center into the cluster.^47^ Additional weak bands can be observed for all other NiFd species (Fig. 4). The Raman spectrum does not change significantly upon CN^−^ binding, with the major vibrational band from the M–S_bridging_ mode remaining at 333 cm^−1^. In contrast, CO binding to the NiFd_red_ cluster significantly changes the low frequency region of the Raman spectrum. The dominant vibrational band representing the M–S_bridging_ mode shifts to 342 cm^−1^. The bands at 365 and 386 cm^−1^ shift slightly to lower energy in the presence of ^13^C-labeled CO, consistent with CO displacement coupling into these modes. As the shifts are substantially lower than those estimated for a local Ni–C or Fe–C oscillator, the CO motion in those bands must be coupled into other vibrational modes. Importantly, an additional isotopically sensitive band at 1964 cm^−1^ is present in the NiFd–CO species, which shifts to 1921 cm^−1^ for NiFd-^13^CO (Fig. 4 and S16†). This shift is nearly exactly as calculated for a local C–O oscillator, suggesting it corresponds to the *ν*_CO_ mode of the NiFd–CO species. This vibrational frequency is significantly lower than that of free CO (2170 cm^−1^), indicative of significant π back-donation and activation of the CO bond.

### Near edge X-ray absorption spectra of NiFd species highlight electronic structure changes localized at the Ni center

**Fig. 5:**
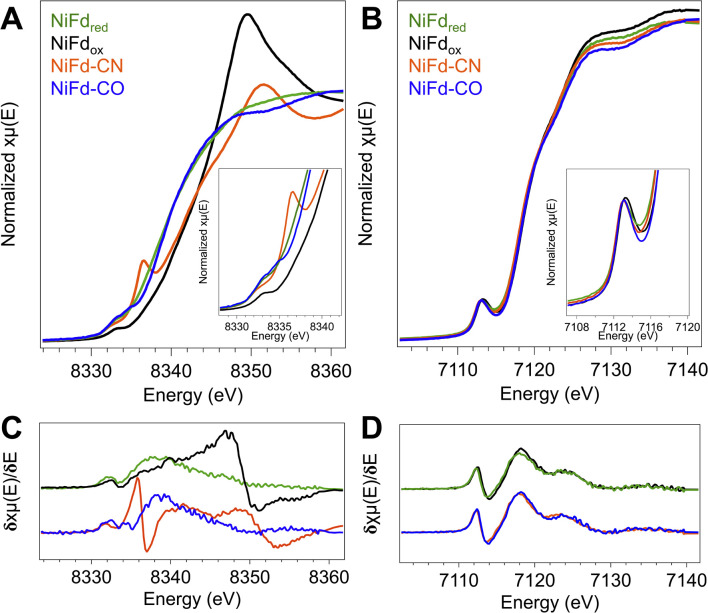
(A) Ni K-edge XANES of the four isolated forms of NiFd. (Inset) Zoom in on the pre-edge region. (B) Fe K-edge XANES of the four isolated forms of NiFd. (Inset) Zoom in on the pre-edge region. (C) Derivative of the Ni K-edge XANES from the traces in A. (D) Derivative of the Fe K-edge XANES from the traces in B.

In order to obtain element-specific information on the electronic and geometric structure of the different NiFd species, both Ni and Fe K-edge X-ray absorption spectroscopies were used (Fig. 5). The edge transition energy of NiFd_ox_ occurs at 8343.8 eV, accompanied by a low-intensity, pre-edge feature located at 8333 eV. This feature arises from the formally dipole forbidden 1s–3d transition, which gains intensity from mixing of the 4p orbitals with the 3d t_2_ orbitals in non-centrosymmetric geometries (*e.g.*, tetrahedral).^48–51^ Upon reduction of the cluster to the NiFd_red_ state, the edge position shifts by −4.2 eV to 8339.6 eV, consistent with an increase of electron density at the nickel center. We note that making formal and physical oxidation state assignments for Ni ions using edge positions alone is challenging, as the edge position is not only dependent on the physical oxidation state but is also impacted strongly by metal–ligand covalency and geometry.^48,51,52^ The pre-edge transition of NiFd_red_ occurs at a very similar energy and intensity as that of the oxidized cluster, suggesting the Ni center remains in a tetrahedral (or distorted tetrahedral) geometry. Interestingly, the XAS spectrum of NiFd–CN shows significant changes when compared to NiFd_red_. The edge position shifts higher in energy to 8342.2 eV, appearing at a similar energy as NiFd_ox_. More importantly, the cyanide-bound cluster exhibits an intense pre-edge feature at 8336 eV and a very low intensity pre-edge feature at 8332.4 eV, suggesting a change in the geometric structure. Previous studies on model nickel compounds have also observed an intense pre-edge feature around 8336 eV, which is suggested to derive from a 1s–4p transition induced by substantial mixing of the d and p orbitals in a low-spin, square planar geometry.^48,49^ On the other hand, CO binding to NiFd_red_ shifts the edge energy only slightly, to 8339.8 eV, with the appearance of a similarly intense pre-edge feature at 8334.8 eV. The second pre-edge feature observed in the spectrum at 8333 eV belongs to residual NiFd_red_ that is not bound to CO. The relative edge shift between NiFd–CO and NiFd_red_ suggests only a modest decrease in electron density at the Ni center upon CO binding.

### Ni K-edge EXAFS provides insight into structural changes at the Ni site of NiFd

**Fig. 6:**
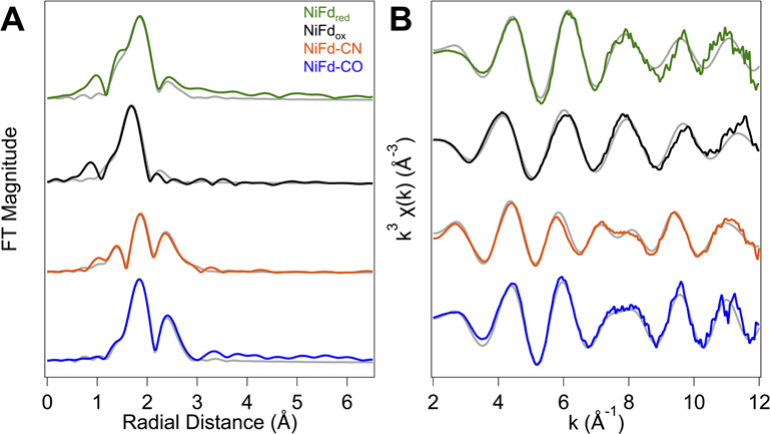
Ni K-edge EXAFS of the four isolated forms of NiFd. (A) Comparison of experimental Fourier transform (FT) EXAFS data (solid) for the four different forms of NiFd overlaid with the best fit (gray). (B) Comparison of experimental k^3^ EXAFS data (solid) for the four different forms of NiFd overlaid with the best fit (gray).

In addition to information on electron density and local geometry, the Ni K-edge XAS spectra provide solution-phase structural information through analysis of the EXAFS region (Fig. 6). The best fit to the data for the NiFd_ox_ was obtained with a primary shell consisting of one N/O atom, three S atoms, and three Fe atoms at distances of 2.01, 2.22, and 2.65 Å, respectively (Table 1). The fit for the NiFd_red_ is similar to that of the oxidized cluster, with contributions from one N/O, three S, and three Fe atoms at distances of 1.95, 2.27, and 2.65 Å, respectively. The distance along the S scattering pathways lengthens slightly, consistent with reduction of the Ni center and in line with previously observed trends for reduction of other [Fe_4_S_4_] clusters using Fe K-edge EXAFS.^53^ In both the reduced and oxidized states of NiFd, the EXAFS data suggest the nickel center retains coordination to three bridging sulfide ligands and a single water/aspartate residue (Fig. S20, S21 and Tables S1, S2†).

**Table 1:** Ni K-edge EXAFS fit results for NiFd_ox_, NiFd_red_, NiFd–CO, and NiFd–CN species giving number of scatters (*n*), interatomic distances (*r*) and Debye–Waller factors (*σ*^2^)

	NiFd_red_	NiFd_ox_	NiFd–CO	NiFd–CN
**Ni–O shell**				
*n*	1	1	—	1
*r* (Å)	1.95	2.01	—	1.87
*σ* ^2^ × 10^3^ (Å^2^)	0.32	3.72	—	3.20

**Ni–C shell**				
*n*	—	—	1	1
*r* (Å)	—	—	1.80	1.88
*σ* ^2^ × 10^3^ (Å^2^)	—	—	5.84	12.47

**Ni–S shell**				
*n*	3	3	3	2
*r* (Å)	2.27	2.22	2.29	2.29
*σ* ^2^ × 10^3^ (Å^2^)	3.48	6.94	6.35	5.76

**Ni–Fe shell**				
*n*	3	3	3	3
*r* (Å)	2.65	2.65	2.73	2.75
*σ* ^2^ × 10^3^ (Å^2^)	13.88	20.77	7.47	12.92
Δ*E*_o_	4.481	−8.72	3.160	1.989
*R*-factor	0.074	0.054	0.042	0.039
Reduced *χ*^2^	1 552 690	7 349 274	1 001 669	2 848 655

The NiFd–CN species showed significant changes in the XANES region, suggestive of substantial distortion towards a square planar structure. With this information, the preferred best-fit model of the experimental EXAFS data includes one O/N, one N/C, two S, and three Fe scattering pathways at distances of 1.87, 1.88, 2.29, and 2.73 Å, respectively, implicating loss of a bridging sulfide ligand. A significantly more intense peak at *R* + Δ = 2.3 Å in the *R*-space data is also observed. This feature is attributed to contributions from both a Ni–Fe single scattering pathway as well as the Ni–CN multiple scattering pathways (Fig. S22 and Table S3†). The EXAFS trace of NiFd–CO is similar to that of the NiFd–CN, including the intense peak in the *R*-space data at *R* + Δ = 2.3 Å. Including the parameters for residual NiFd_red_ in a two-component fit to the EXAFS *k*- and *R*-space traces, the best fit to the data includes one C/N, three S, and three Fe pathways at distances of 1.80, 2.29, and 2.73 Å, respectively, suggesting that the three sulfide bridges remain intact with loss of the carboxylate ligand (Fig. S23 and Table S4†).

### Fe K-edge XANES suggest minor changes at Fe across cluster oxidation states

The changes in the edge and pre-edge positions of the different NiFd species are significantly less pronounced in the Fe K-edge XAS spectra. The Fe K-edge XANES of the [Fe_3_S_4_] Fd and the [Fe_4_S_4_] Fd species were measured for comparison (Fig. S24†) and observed to follow the trends previously reported for other known biological and synthetic iron–sulfur clusters.^50,53–55^ The Fe edge positions of the four isolated forms of NiFd also lie within a narrow range, with values at ∼7119 eV for the NiFd_ox_, NiFd_red_, NiFd–CN, and NiFd–CO, respectively (Fig. 5). While the differences are small (<1 eV), the edge position does follow a general trend across the series, where NiFd–CN < NiFd–CO ≈ NiFd_red_ < NiFd_ox_. Similarly, the pre-edge feature at 7113.2 eV does not change significantly when the cluster is reduced or bound to the small ligands. The insensitivity of the Fe K-edge XANES features across all species suggests that the electronic structure perturbations introduced by oxidation state changes and binding of small molecules are predominantly localized to the Ni center.

### DFT geometry optimization suggests substrate binding can occur with minimal perturbation to protein structure

**Fig. 7:**
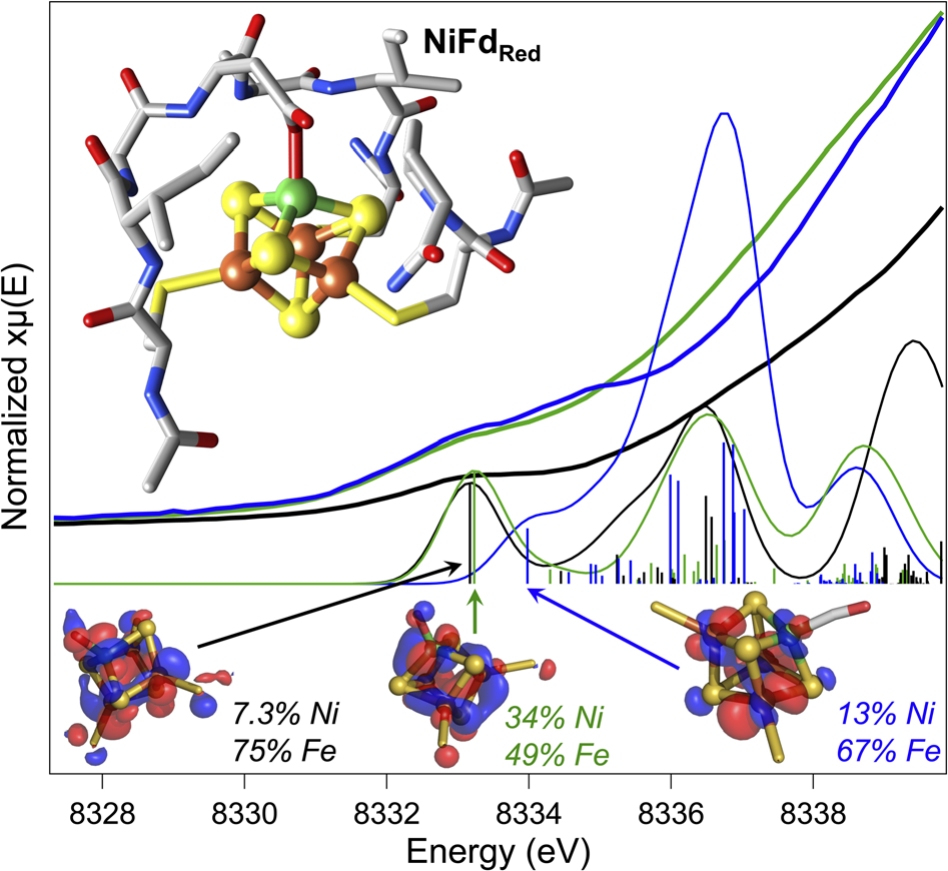
Experimental pre-edge Ni K-edge XANES spectra of NiFd_red_ (green), NiFd_ox_ (black), and NiFd–CO (blue) with calculated TD-DFT contributions using the DFT geometry-optimized structures of the NiFd species (inset). Dominant contributing orbital to the indicated transition for each species shown at an isosurface value of 0.03 with distribution over Ni and the Fe atoms indicated.

At present, there are no published X-ray crystal structures of the WT *Pf* Fd in the [MFe_3_S_4_] state, and we were not able to obtain crystals of NiFd in any oxidation state.^56,57^ To gain some information on the interaction between the metallocofactor and protein environment, density functional theory calculations were used. A computational model was constructed that included the ligating residues and secondary coordination sphere (Fig. 7). Geometry optimizations were performed for the cluster assuming local and global high spin configurations, which overestimates bond lengths but allows us to qualitatively consider general trends across redox state changes and upon CO binding (Tables S7 and S8†).^58^ This method was not deemed appropriate for calculations on the NiFd–CN state because of the XAS evidence in favor of a low-spin Ni^II^ center (*vide supra*). Minimal structural perturbations were observed upon cluster reduction from the NiFd_ox_ to the NiFd_red_ state, with all metal centers retaining a tetrahedral geometry (Fig. S25–27 and Table S7†). Two conformations of the Asp14 ligand could be optimized for the CO-bound state, one in which the aspartate remains ligated and a Ni–sulfide bond breaks, and another in which the aspartate rotates away from the cluster (Fig. S28–S30 and Table S8†). The EXAFS data and analyses led us to favor the latter geometry, in which the aspartate coordination to the cluster is broken (Fig. S29 and S30†), which preserves tetrahedral geometry at the nickel center and gives a near-linear Ni–C–O bond of 170°. No structures converged in which CO was bound to any of the Fe centers.

Preliminary validation of the optimized geometries was supported through comparison of the calculated and experimental Ni K-edge XAS pre-edge energies and intensities (Fig. 7). The calculated pre-edge transitions of NiFd_ox_ and NiFd_red_ exhibit similar pre-edge energies and intensities while the NiFd–CO pre-edge transition is shifted to higher energy. In all cases, the dominant orbitals contributing to the transitions are delocalized across the Ni and the three Fe centers. The general agreement between the calculated and experimental data supports the premise that CO binding and redox state changes can occur without inducing large structural perturbations to the protein scaffold.

## Discussion

### A high density of states is present in the NiFd–CO species

Given the importance of the C_red1-CO_ state in CODH, we sought to obtain detailed information on the electronic and geometric structure of the CO-bound state(s) of NiFd. Through a holistic analysis of the results from several complementary spectroscopic studies, as reported above, we propose that the NiFd–CO species possesses a ground spin state of *S* = 1/2 with a low-lying, *S* = 3/2 excited state within a cuboidal [NiFe_3_S_4_]^+^–CO cluster. This hypothesis derives from several observations: (1) the temperature-dependent EPR spectra reveal that the *g*_app_ = 3.65 feature relaxes more slowly than the *g*_app_ = 2.05 feature (Fig. 3, S8 and S9†), in contrast to the expected behavior for a standard *S* = 1/2 species;^59^ even the spin-coupled *S* = 1/2 [Fe_4_S_4_]^+^*Pf* Fd species relaxes more slowly than the high-spin component (Fig. S3†).^45^ Moreover, the integrated intensities of the features belonging to the NiFd–CO display a linear relationship as a function of 1/*T* between the temperatures of 6.5–30 K (Fig. S7†), indicating that these two features belong to the same species, with the *S* = 1/2 feature interconverting to the *S* = 3/2 feature at higher temperatures. (2) The small but demonstrable EPR line broadening observed for the ^61^Ni and ^57^Fe labelling suggest both Ni and Fe contribute to the overall spin of the cluster, supporting an integral role of Ni in the cluster and spin scheme. (3) The high-frequency region of the resonance Raman spectrum of NiFd–CO displays a single isotopically sensitive band at 1964 cm^−1^, consistent with the presence of a single species. The low-frequency rR bands are consistent with a cubane-like system and show small shifts upon ^13^CO incorporation, providing further evidence for a CO-bound nickel–iron–sulfur cluster. Considering the XANES, EXAFS, resonance Raman, and EPR data together, we propose a cuboidal structure for NiFd–CO, with tetrahedral coordination at all metals and CO bound to the nickel center. The presence of a low-lying excited state in NiFd–CO may facilitate multistate reactivity in this model system and, by extension, native CODH (*vide infra*).

### Ligand binding triggers electron redistribution throughout the NiFd cluster: formal oxidation state assignments

The electronic structure of iron–sulfur clusters is of significant interest for the study of biological electron transfer and cluster reactivity. The most common configurations of [Fe_4_S_4_] clusters are generally considered well-understood: formally, the oxidized canonical [Fe_4_S_4_]^2+^ cluster is best described as two pairs of mixed-valent Fe^2.5+^ centers that are coupled through a double exchange pathway.^60,61^ Similarly, the reduced cluster can be described as a single mixed valent Fe^2.5+^ pair antiferromagnetically coupled to a pair of Fe^2+^ centers. However, a growing body of work suggests that the formal and physical oxidation states of the iron centers are dictated by the overall bonding environment of the cluster.^32,62^ One such example in biological systems is found in the radical SAM intermediate, Ω, where formation of the organometallic species upon reductive SAM cleavage results in a state that resembles the high-potential iron–sulfur cluster proteins (HiPIPs), with an excess ferric site.^61,63,64^ The physical electronic structure of Ω is still under debate; however, models of this intermediate suggest that the unique Fe bound to the SAM fragment may adopt a local Fe^III^ electronic configuration.^32^ Similar electronic rearrangement has been observed in synthetic systems, where recent work from the Suess group has shown that binding of exogenous ligands to one unique site of a model [Fe_4_S_4_] cluster influences the oxidation states of the other irons within the cluster.^32,62,65^ This isomerization is likely linked to the close electronic communication between the metal centers through double exchange pathways within the cluster.

**Fig. 8:**
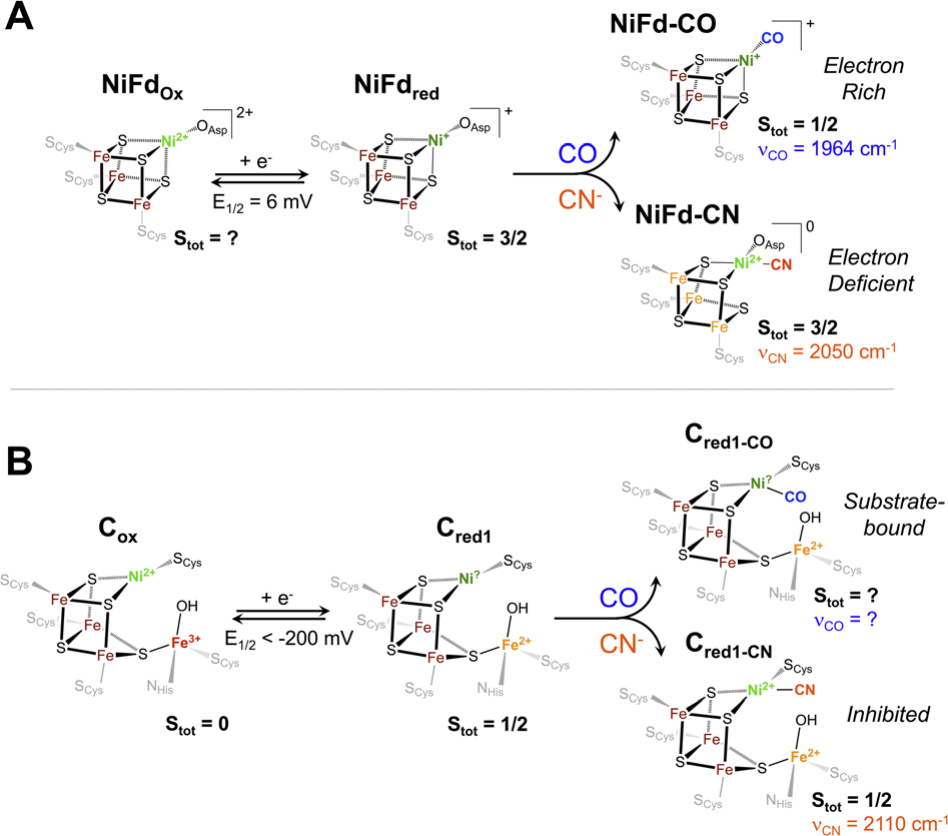
Proposed electronic and geometric structures of the (A) NiFd_ox_, NiFd_red_, NiFd–CO, and NiFd–CN species with key experimental metrics indicated that have been obtained from this work, and (B) analogous C_ox_, C_red1_, C_red1-CO_, and C_red1-CN_ states of CODH with spectroscopic metrics obtained from ref. 17, 75 and 76.

Analogous to these prior examples in biological and synthetic [Fe_4_S_4_] clusters, binding of exogenous ligands to NiFd impacts the electronic distribution within the [NiFe_3_S_4_] cluster. The initial reduction event from NiFd_ox_ to NiFd_red_ appears to be dominantly localized on the nickel center with minimal change at the iron centers, as evidenced by the XAS spectra. The similarity in the pre-edge features of NiFd_ox_ and NiFd_red_ and comparison to well-defined synthetic compounds suggests a tetrahedral nickel center.^48,49,51,52^ While formal oxidation state assignments of metals provide only a rough connection to the physical oxidation states, particularly in systems with highly covalent bonding, the experimental evidence supports the assignment of a high-spin Ni^II^ in the NiFd_ox_ species and a Ni^I^ center in the NiFd_red_ species (Fig. 8), with a formally assigned [Fe_3_S_4_]^0^ cluster fragment for both states respectively. The highly activated CO ligand (*ν*_CO_ = 1964 cm^−1^) in NiFd–CO is consistent with significant electron donation into the π* orbitals of the CO ligand and is in line with other known Ni^I^–CO complexes.^66,67^ Typical four-coordinate Ni^II^–CO and Ni^0^–CO compounds exhibit *ν*_CO_ values of ∼2060 and 1830 cm^−1^, respectively.^68–71^ Considering *ν*_CO_ along with the XAS edge energy, which is similar to that of NiFd_red_, a Ni^I^–CO and [Fe_3_S_4_]^0^ configuration is suggested to be present in the NiFd–CO species. On the other hand, back-donation into the CN^−^ π* orbitals is relatively small in NiFd–CN, as the *ν*_CN_ is observed at 2050 cm^−1^, a small shift from free cyanide (*ν*_CN_ = 2080 cm^−1^).^41^ This vibrational frequency is consistent with other Ni^II^–CN complexes.^72^ The XANES spectra provide further evidence for differences in electronic structure at the nickel center. The edge position shifts to higher energies relative to NiFd_red_ or NiFd–CO and instead overlaps with that of NiFd_ox_, suggesting a significant loss of electron density from the nickel center. Additionally, the intense pre-edge feature that is observed at 8336 eV derives from the 1s–4p transition and is only prevalent in low-spin, square planar Ni^II^ species.^48,49,52^ Collectively, these observations suggest the presence of a low-spin, Ni^II^ oxidation state in NiFd–CN, with a [Fe_3_S_4_]^−^ fragment (Fig. 8). A shift in physical oxidation state for NiFd–CN relative to NiFd_red_ would suggest the electron density is redistributed back into the [Fe_3_S_4_] fragment. Indeed, careful analysis of the Fe K-edge XAS spectrum does show a shift of the NiFd–CN edge position to lower energy by approximately 0.2 eV, reflecting an average increase in electron density across the three iron centers (Fig. 5). Due to the highly delocalized nature of iron sulfur clusters, we would expect additional electron density to be further distributed across the bridging sulfide and terminal ligands. These analyses provide direct evidence for electronic communication and fluidity of electron density between the four metal centers, highlighting a potential mechanism for promoting multielectron chemistry within heterometallic clusters.

### Implications for substrate and inhibitor binding in native CODH

The Ni K-edge XANES spectrum of NiFd_red_ has remarkably similar features to previously observed spectra of reduced *Rr* CODH and other synthetic [NiFe_3_S_4_] clusters, including a weak pre-edge feature at 8333 eV.^49^ Oxidation of *Rr* CODH to the catalytically inactive C_ox_ state does not cause significant changes to the Ni K-edge XAS spectra, suggesting the redox state changes are not localized to the Ni center; instead, Mössbauer and Fe K-edge XAS spectroscopy have suggested the initial reduction event to activate CODH occurs at the pendant iron center, generating the resting state C_red1_ (Fig. 8).^17,49,73^ In the absence of an exogenous Fe ion, as in NiFd, the redox state changes are localized on the substrate-binding Ni center (Fig. 8), providing benchmarks for the limiting cases that might be observed throughout CODH catalysis (Fig. 5 and S24†). More parallels are observed in comparing the EXAFS data of reduced *Rr* CODH and NiFd_red_, as both species have an overall coordination number of four and similar distances to the bridging sulfide ligands. Like the spectra of *Rr* CODH, the NiFd_red_/NiFd_ox_ do not exhibit an intense Ni–Fe scattering feature that is present in the synthetic [NiFe_3_S_4_] cubane clusters. We have interpreted the latter observation to indicate that, in the absence of a small-molecule ligand, the NiFd cubane is not sufficiently rigid to resolve a clear Ni–Fe scattering vector.^49^ The Ni–Fe scattering pathway becomes more pronounced in the NiFd–CO and NiFd–CN samples, suggesting that binding of CO or CN^−^ rigidifies the cluster (Fig. S22, S23 and Tables S3–S4†). This may be the case for the [NiFe_3_S_4_] subsite of CODH as well, allowing conformational flexibility prior to substrate binding.

The EPR data show parallels to native CODH and other [NiFe_3_S_4_] synthetic models. The EPR spectra of C_red1_ and the cyanide-inhibited state, C_red1-CN_, both have total ground spin states of *S*_tot_ = 1/2 with *g*_avg_ = 1.82 and 1.72, respectively.^17,74,75^ From the Mössbauer and EPR data, this *S*_tot_ = 1/2 ground state is suggested to arise from antiferromagnetic coupling between the *S* = 3/2 [NiFe_3_S_4_] subsite and the *S* = 2 exogenous Fe subsite. The NiFd_red_ state has a ground spin state of *S* = 3/2, which is directly analogous to that of the [NiFe_3_S_4_] subsite of the C_red1_ state.^17,49^ Additionally, the electronic parameters of the NiFd_red_ species are similar to those of synthetic cubane [NiFe_3_S_4_]^+^ models by Holm and coworkers, which also have ground spin states of *S* = 3/2.^34,49^ In the case of NiFd_red_, we postulate this spin state likely arises from the coupling of an *S* = 1/2 Ni^I^ center to the [Fe_3_S_4_]^0^ fragment on the basis of the data presented here. Experiments using Mössbauer spectroscopy are currently underway to more precisely determine the local spin states and coupling scheme of Fe centers within the cluster. Additionally, like the native system, only small line-broadening is observed upon substitution of ^61^Ni,^43^ which may highlight the importance of electronic cooperativity and spin delocalization between the metal centers for reactivity in CODH. Binding of CN^−^ to NiFd_red_ preserves the *S* = 3/2 ground spin state but perturbs the EPR spectrum in a manner that is similar to the changes observed for synthetic cubane [NiFe_3_S_4_]^+^ models; the spin state of the cubane in native CODH is also suggested to remain *S* = 3/2 on the basis of coupling with the exogenous high-spin ferrous ion (Fig. 8).

The proposed geometry of the nickel center in NiFd–CN is also similar to that observed in the cyanide-bound structure of CODH II_Ch_; however, the *ν*_CN_ (2110 cm^−1^) of the native enzyme is notably higher than that in the NiFd–CN state.^76^ We believe this discrepancy derives from the differences in hydrogen bonding environments for the two systems. In native CODH, proposed hydrogen bonding interactions with secondary sphere residues may promote greater *σ*-donating and less π-accepting character from the CN^−^ ligand, giving rise to higher *ν*_CN_ vibrational frequencies.^77^

Though CO and CN^−^ are isoelectronic and often considered interchangeable in the context of structure and bonding, it is evident that each of these ligands imparts unique behavior to the nickel-substituted iron–sulfur cluster in NiFd. It is thus worth considering the subtle distinctions in electronic structure between the two small ligands. Both CO and CN^−^ bind to the Ni^I^ center in the stable NiFd_red_ state and to the nickel center in C_red1_,^76,78^ driving a natural comparison between the two systems across ligands. CO is a neutral ligand with strong π-accepting character, underpinning the tendency to bind to electron-rich metals,^79^ while the anionic CN^−^ ligand has greater *σ*-donor ability and more capacity for hydrogen bonding.^80^ The carbon center in CN^−^ is therefore more nucleophilic than in CO, which likely preferentially stabilizes the Ni^II^ oxidation state and drives the redistribution of electron density across the cluster in NiFd.^62^ This intracluster isomerization may hint at the mechanism through which CN^−^ inhibits turnover of CODH. When CN^−^ binds to the nickel center, which is suggested to be the binding site from multiple structural studies,^76,78^ the nucleophilicity of the carbon center may drive the nickel to adopt a formal divalent oxidation state, effectively trapping the system in a configuration that is more electron-deficient at the nickel center (Fig. 8). This electron-deficient state may stabilize the system in the presence of oxidants, as recent work suggests the CN-bound C-cluster is more resistant to O_2_ damage than the resting state, but requires reductive activation to re-enter the catalytic cycle.^12,81^ On the other hand, the electron-rich Ni^I^ center in NiFd_red_ is stabilized upon CO binding. The large degree of activation of the substrate CO ligand suggests a similarly nucleophilic Ni^I^ state may be present in the catalytically active C_red1_ and elusive C_red1-CO_ states (Fig. 8). The temperature-dependent EPR data on NiFd–CO may also provide insight into the absence of a known C_red1-CO_ signal. While the ground spin state of NiFd–CO is suggested to be *S*_tot_ = 1/2, it is not well-isolated, as evidenced by the clear appearance of high-spin signals at cryogenic temperatures. We consider it possible that low-lying excited states of C_red1-CO_ may be present in native CODH that could complicate EPR observation. Moreover, the presence of the exogenous Fe center in the C-cluster is expected to affect the electronic structure of the spin system. The absence of a nearby nucleophilic site in NiFd–CO likely minimizes reactivity, permitting facile observation of the CO-bound state. Access to excited states in NiFd may also indicate potential for multistate reactivity in CODH, though this discussion would require advanced computational analysis that is beyond the scope of this work.^45,82^ The distinct differences observed between the CN^−^ and CO underscore the importance of understanding the interactions between enzymes and their native substrates.

## Conclusions

A nickel-substituted [NiFe_3_S_4_] ferredoxin (NiFd) protein was investigated as a model of the cubane subsite of carbon monoxide dehydrogenase (CODH). The NiFd system was prepared in several oxidation and substrate-bound states to mimic key states of native CODH. Through the use of EPR, rR, K-edge XANES, and EXAFS spectroscopies, we have assigned the redox processes observed to be formally centered on nickel and characterized the NiFd–CO species as a Ni^I^–CO nickel center that has tetrahedral coordination. Cyanide binding induces redox isomerization, resulting in a low-spin, square planar nickel center and increased electron density spread across the iron atoms in the cluster. This is in line with phenomena previously observed within synthetic iron–sulfur clusters, providing evidence for the ability of heterometallic cubane clusters to shuffle electron density around within the cluster. Furthermore, extrapolating the results found using this protein-based model, we have postulated that similar phenomena may occur in native CODH, thus providing insight into the mechanism of inhibition by CN^−^ in the native system. Ultimately, the results found from this study highlight the utility of developing protein-based models of enzymes.

## Data availability

All raw and processed data generated in this study, including data presented in the main manuscript and the ESI† are freely available upon request.

## Author contributions

Collection of EPR, FTIR, optical, resonance Raman, and Ni/Fe K-edge XAS spectroscopies along with all sample preparation was done by LCL. The computational DFT results were completed by JASG. Analysis of all data was performed by LCL and HSS. YL assisted in collecting XAS data. AJJ prepared the proposal for beamline time and assisted in collection of the XAS data. The manuscript was written by LCL and HSS. HSS was responsible for oversight and management of the project.

## Conflicts of interest

There are no conflicts to declare.

## Supplementary Material

SC-015-D4SC00023D-s001
