# Supplementary material for: Electronic isomerism in a heterometallic nickel–iron–sulfur cluster models substrate binding and cyanide inhibition of carbon monoxide dehydrogenase
Source: Chem Sci. 2024 Mar 27;15(16):5916–28. doi: 10.1039/d4sc00023d (PMC11040638; doi:10.1039/d4sc00023d)
Supplement: SC-015-D4SC00023D-s001 [file SC-015-D4SC00023D-s001.pdf]

Electronic Supplementary Material (ESI) for Chemical Science.
This journal is © The Royal Society of Chemistry 2024

Electronic Isomerism in a Heterometallic Nickel-Iron-Sulfur Cluster Models Substrate Binding and Cyanide Inhibition of Carbon Monoxide Dehydrogenase

Luke C. Lewis[a], José A. Sanabria-Gracia[a], Yuri Lee[a,b], Adam J. Jenkins[a], and Hannah S. Shafaat[a,b,*]

[a] *Department of Chemistry and Biochemistry, The Ohio State University, Columbus, OH 43210.*

[b] *Department of Chemistry and Biochemistry, University of California, Los Angeles, Los Angeles, CA 90095.*

Supporting Information

Table of Contents

Materials and Methods **S3-7**

Figure S1. CW EPR spectra of ^{57}Fe and ^{61}Ni NiFd species **S8**
Discussion of ^{57}Fe labelling in NiFd species **S8**
Figure S2. CW EPR of ^{61}NiFd-CO **S9**
Figure S3. VT CW EPR spectra of $[Fe_4S_4]^+$ Fd **S10**
Figure S4. Temperature- and power-dependent EPR spectra of $[Fe_4S_4]^+$ Fd **S11**
Figure S5. EPR spin integration vs. 1/T for $[Fe_4S_4]^+$ Fd **S12**
Figure S6. Curie plot of g = 3.65 and g = 2.05 feature of NiFd-CO **S13**
Figure S7. EPR spin integration vs. 1/T for NiFd-CO **S14**
Figure S8. CW EPR power saturation curve for g = 2.05 feature of NiFd-CO **S15**
Figure S9. CW EPR power saturation curve for g = 3.65 feature of NiFd-CO **S16**
Figure S10. CW EPR power saturation curve for g = 5.7 feature of $NiFd_{red}$ **S17**
Figure S11. CW EPR power saturation curve for g = 5.0 feature of $NiFd_{red}$ **S18**
Figure S12. CW EPR power saturation curve for g = 4.35 feature of NiFd-CN **S19**
Figure S13. Orbach relaxation fits for NiFd species **S20**
Figure S14. Curie plot of g = 4.35 feature of NiFd-CN **S21**
Figure S15. FTIR spectrum of NiFd-CO **S22**
Figure S16. Gaussian fits of resonance Raman spectra of NiFd-$^{NA/13}$CO collected at 407 nm **S23**
Figure S17. Optical and EPR spectroscopy on representative resonance Raman samples **S24**
Figure S18. Resonance Raman spectra of NiFd species collected at 458 nm **S25**
Figure S19. Resonance Raman spectra of NiFd-$^{NA/13}$CN collected at 458 nm **S26**
Figure S20. R-space and k-space fit for $NiFd_{ox}$ with scattering pathways **S27**
Figure S21. R-space and k-space fit for $NiFd_{red}$ with scattering pathways **S28**
Figure S22. R-space and k-space fit for NiFd-CN with scattering pathways **S29**
Figure S23. R-space and k-space fit for NiFd-CO with scattering pathways **S30**
Table S1. EXAFS fits for $NiFd_{ox}$ **S31**
Table S2. EXAFS fits for $NiFd_{red}$ **S32**
Table S3. EXAFS fits for NiFd-CN **S33**
Table S4. EXAFS fits for NiFd-CO **S34**
Table S5. EXAFS fits for NiFd-CN using C/N/O scattering pathway **S35**
Table S6. EXAFS fits for NiFd-CO using C/N/O scattering pathway **S36**
Discussion of Ni K-edge EXAFS Fits **S36-38**
Figure S24. Fe K-edge XANES of Fd species **S39**
Table S7. DFT-optimized geometric parameters for $NiFd_{red}$/$NiFd_{ox}$ model **S40**
Figure S25. DFT-optimized structure of $NiFd_{ox}$ model **S41**
Figure S26. DFT-optimized structure of $NiFd_{red}$ model **S42**
Figure S27. Overlay of DFT-optimized structures of $NiFd_{red}$ and $NiFd_{ox}$ models **S43**
Table S8. DFT-optimized geometric parameters for $[NiFe_3S_4]^+$-CO models **S44**
Figure S28. DFT-optimized structure of $[NiFe_3S_4]^+$-CO model with D14 ligation **S45**
Figure S29. DFT-optimized structure of $[NiFe_3S_4]^+$-CO model without D14 ligation **S46**
Figure S30. Overlay of DFT-optimized structures of $NiFd_{red}$ and NiFd-CO models **S47**
Figure S31. FWHM determination for g = 5.7 of $^{NA/61}NiFd_{red}$ CW EPR spectra **S48**
Figure S32. CW EPR spectra of NiFd-CO with glassing agent **S49**
Figure S33. CW EPR spectra of XAS samples of $NiFd_{red}$ and $NiFd_{ox}$ **S50**
Figure S34. Experimental and simulated CW EPR spectra of NiFd-CN **S51**

Appendix S1. Sample ORCA Input File **S52-54**
Supplemental References **S55**

Detailed Materials and Methods

Expression, lysis, and purification of WT Pf Fd

Pure samples of WT *Pf* Fd were obtained as described previously.[1] The plasmid (pTrc99aPfFd) containing the gene for fdxA for WT *Pf* was received as a gift from Professor Michael Adams (University of Georgia). The plasmid was transformed into chemically competent JM105 *E. coli* cells (ATCC 47016). A 300 mL culture of Luria-Broth (LB) media containing 70 μg/mL of carbenicillin (Gold-Bio) was grown at 37 ºC while shaking at 200 RPM for 17 h. 50 mL of this culture was then used to inoculate 2 L of LB media supplemented with 70 μg/mL carbenicillin and 25 μM $FeCl_3$ (Ward's Science) in a 6 L flask. The culture was grown to OD_{600} = 0.4 at 30 ºC shaking at 200 RPM, at which point it was supplemented with 250 μM L-cysteine (BioBasic Canada). At OD_{600} = 1.0, protein expression was induced by addition of 1 mM IPTG (Gold-Bio) at 30 ºC shaking at 200 RPM for 12 hrs. The cells were harvested by centrifugation at 6800 xg for 7 min at 4 ºC. Finally, the cells were washed with 50 mM Tris-HCl buffer (VWR) and stored at -80 ºC until lysis.

To lyse the cells, the cell pellet was resuspended in 3 mL/g cell pellet of 10 mM Tris-HCl buffer, pH 8.0, containing 100 mM NaCl (Fisher), 1 mM EDTA (VWR), 5 mM PMSF (GoldBio), and 0.33 mg/mL of lysozyme (GoldBio). The suspension was then incubated at 30 ºC shaking at 200 RPM for 1 hr, then the suspension was sonicated to further lyse the cells. Next, 50 μg/mL of DNAse I (Gold-Bio) was added to the lysed cells, and the suspension was incubated for an additional 30 min at 30 ºC shaking at 100 RPM. To precipitate additional *E. coli* proteins from the lysis solution, the suspension was heated to 65 ºC for 1 h. Cellular debris was then pelleted out by centrifugation at 39,000 xg for 30 min at 4 ºC.

The resulting lysate was buffer-exchanged into 50 mM Tris-HCl buffer, pH 8.0, and loaded onto a HiTrap™ Q FF (GE) anion exchange column. The protein was eluted using a 100 mL linear gradient from 0 to 0.6 M NaCl in Tris-HCl pH 8.0. Fractions containing *Pf* Fd WT were pooled together and dialyzed overnight into 5 mM sodium acetate, pH 3.0. Any resulting precipitate was pelleted out by centrifugation at 39,000 xg for 10 min at 4 ºC. The lysate was then loaded onto a HiTrap™ SP HP (GE) cation exchange column in 5 mM sodium acetate buffer, pH 3.0. The protein was eluted using a 100 mL linear gradient from 0 to 0.6 M NaCl. Fractions containing *Pf* Fd were collected and exchanged into 50 mM Tris-HCl buffer, pH 8.0. This solution was concentrated and loaded onto a Sephadex G-75 size exclusion column (hand-packed) and eluted using 50 mM Tris-HCl buffer, pH 8.0. Pure fractions were collected and concentrated, before treatment with 50 mM $KFeCN_6$ (Sigma) and 10 mM EDTA at RT for 2 h. The protein solution containing $[Fe_3S_4]^+$ Fd was then desalted using a 10-DG Desalting Column (Bio-Rad) to remove excess iron and ferricyanide and was then concentrated and stored at 4 ºC. Protein purity was verified using SDS-PAGE.

Isotopic Labelling of $[NiFe_3S_4]$ Fd with ^{61}Ni and ^{57}Fe

Preparation of $[^{61}NiFe_3S_4]$ Fd was performed following the same procedure as above using $^{61}Ni(NO_3)_2$ as the nickel source. Reconstitution of the cluster with ^{57}Fe was performed as described previously.[2] Freshly purified $[Fe_3S_4]$ Fd in 25 mM Tris-HCl buffer, pH 8.0, was supplemented with 100 mM DTT (GoldBio) for 15 min before being treated with 16% TCA (RICCA Chemical) for 30 min at 25 ºC. The resulting precipitated protein was pelleted out by centrifugation at 5000 xg and resuspended in 500 mM Tris-HCl, pH 8.0. This process was performed two more times to ensure efficient conversion to apo-Fd. The apo-Fd was treated with 100 mM DTT for 30 min, then 16 molar equivalents of ^{57}Fe and Na_2S were added in four 30-min increments. The resulting solution was allowed to reconstitute overnight. The excess FeS was pelleted out by centrifugation at 10,000 xg, and the supernatant was loaded onto a 10-DG desalting column equilibrated with 50 mM Tris-HCl buffer, pH 8.0, to remove unreacted metal and salts. The protein fractions were then collected and loaded onto a HiTrap™ Q FF (GE) anion exchange column. The protein was eluted using a 100 mL linear gradient from 0 to 0.6 M NaCl in Tris-HCl buffer, pH 8.0. Pure fractions were collected and concentrated, before treatment with 50 mM $KFeCN_6$ (Sigma) and 10 mM EDTA at RT for 2 h. The protein solution containing $[Fe_3S_4]^+$ Fd was then desalted using a 10-DG desalting column (Bio-

Rad) to remove excess iron and ferricyanide and was then concentrated and stored at 4 °C. Reconstitution with Ni was performed as mentioned previously using $Ni(NO_3)_2$.

Preparation of $^{61}Ni(NO_3)_2$.
The $^{61}Ni(NO_3)_2$ solution was prepared by dissolving isotopically enriched ^{61}Ni (99.42 %) metal (ISOFlex USA) in 4 molar equivalents of HNO_3 at 37 °C, shaking at 200 RPM for 2 hrs. Polished platinum wire was added to the vessel to assist in dissolution. After total dissolution, the platinum wire was removed, and the solution was titrated with KOH to a final pH ~ 6 and stored at -80 °C until ready to use.

Preparation of $^{57}FeSO_4$.
The $^{57}FeSO_4$ solution was prepared by dissolving isotopically enriched ^{57}Fe (96.18%) metal (Cambridge Isotope Labs) with 2 molar equivalents of H_2SO_4 at 37 °C at 200 RPM for 12 hrs. After total dissolution, the solution was stored at -80 °C until ready to use.

Reconstitution of [MFe_3S_4] (M = Ni, Fe) cluster in WT Pf Fd.
Preparation of [MFe_3S_4]$^+$ Fd samples was performed as described previously.[1] To prepare [MFe_3S_4]$^+$ Fd samples, the purified [Fe_3S_4]$^+$ was reduced with 3 molar equivalents of sodium dithionite (DT, Beantown-Chemical) for 20 min under an anaerobic atmosphere. After the cluster had been fully reduced, 20 molar equivalents of $Ni(NO_3)_2$ (Sigma-Aldrich) or $(NH_4)_2Fe(SO_4)_2$ (Sigma-Aldrich) was added and allowed to incubate for 2 h under an anaerobic atmosphere. The resulting protein solution was then desalted using a 10-DG Desalting Column to remove excess metals and dithionite. Oxidized samples were prepared by first following the protocol above. The protein was then incubated with 20 molar equivalents of thionin acetate (VWR) for 10 min.

EPR Sample Preparation
All samples were prepared in an anaerobic chamber (<10 ppm O_2, Vigor Technologies). The 1 mM [$NiFe_3S_4$]$^+$ Fd EPR sample was prepared by incubating freshly desalted and concentrated [$NiFe_3S_4$] Fd with 4-fold molar excess DT for 10 min. The solution was then transferred into a quartz EPR tube and flash frozen in liquid nitrogen. The [$NiFe_3S_4$]-CN Fd sample was prepared by first preparing the [$NiFe_3S_4$]$^+$ Fd sample as described above. the protein was incubated with 4-fold molar excess DT and 30-fold molar excess KCN (Millipore Sigma) for 10 min. A small amount (< 5%) of [Fe_4S_4] Fd contamination was identified using EPR Spectroscopy at elevated temperatures (T > 10 K) (**Figure S34**). The [$NiFe_3S$]$^+$-CO Fd sample was prepared by incubating 1 mM of [$NiFe_3S_4$] Fd with 10 mM DT for 10 min. The solution was then loaded into a 2 mL borosilicate vial with a crimp seal, and pressurized to 40 psi CO. After 5 min, 200 µL of the sample was loaded into an EPR tube (Wilmad Lab-Glass 727-SQ-250M) and flash frozen in liquid nitrogen. *Caution! Cyanide samples must be handled at basic pHs (pH > 8) to avoid outgassing of toxic HCN fumes. All CO manipulation was carried out in a glovebox that was directly vented.*

Optical Spectroscopy
Absorption spectroscopy was carried out in an anaerobic chamber (<10 ppm O_2, Vigor Technologies) using a Cary 60 UV-Vis spectrophotometer (Agilent Technologies) using a pathlength of 0.2 cm. For the [Fe_3S_4]^0 Fd sample, purified [Fe_3S_4]$^+$ Fd was reduced with 4 molar equivalents of DT and incubated for 20 min. The protein solution was then desalted using a 10-DG desalting column equilibrated with 25 mM phosphate (VWR) buffer, pH 7.0. The solution was diluted to ~200 µM in 25 mM phosphate buffer, pH 7, supplemented with 2-fold molar excess DT, and transferred into a cuvette. The [$NiFe_3S_4$]$^+$ Fd sample was prepared as described above. After incubation with excess DT, the solution was desalted into 25 mM phosphate buffer, pH 7.0. The solution was diluted to ~200 µM, 2-fold molar excess DT was added, and the solution was transferred to a cuvette. The [$NiFe_3S_4$]-CN Fd sample was prepared by first preparing the [$NiFe_3S_4$]$^+$ Fd sample as described above. After the solution was diluted to ~200 µM using 25 mM phosphate buffer, pH 7.0, the protein was incubated with 2-fold molar excess DT and 30-fold molar excess KCN (Millipore Sigma) for 10 min and transferred to a cuvette. Likewise, the [$NiFe_3S_4$]$^+$-CO Fd sample

was prepared by first preparing the $[NiFe_3S_4]^+$ Fd sample as described above. However, after the solution was desalted, the protein was concentrated to ~1 mM and then diluted to ~200 µM using CO-saturated, 25 mM phosphate buffer, pH 7.0. The solution was then allowed to incubate for 20 min with 2-fold molar excess DT and transferred to a cuvette. All samples were then transferred to individual QSI tubes and checked for quality using EPR spectroscopy (**Figure S17**).

X-ray Absorption Spectroscopy Sample Preparation

All samples were prepared under a nitrogen atmosphere. All samples were loaded into homemade white Delrin sample cells with dimensions of 3 mm x 2 mm x 22.25 mm, covered with a Kapton tape window. Additionally, all samples were prepared in 100 mM CHES (Beantown Chemical) buffer, pH 8.0. For the $[NiFe_3S_4]^+$ and $[NiFe_3S_4]^+$-CO Fd samples, 300 mM sucrose (VWR) was used as a glassing agent in place of 20% glycerol to prevent CO dissociation from the cluster (**Figure S32**). The $[NiFe_3S_4]^+$ Fd sample was prepared by incubating freshly desalted and concentrated $[NiFe_3S_4]$ Fd with 10-fold molar excess DT for 10 min. After 10 min, 300 mM sucrose was added, and 140 µL of 1 mM protein was transferred to a white Delrin sample cell and frozen in liquid nitrogen. For the $[NiFe_3S_4]^+$-CO sample, 2 mM $[NiFe_3S_4]$ Fd was incubated with 10-fold molar excess DT for 10 min. After 10 min, the solution was diluted to a protein concentration of 1 mM with CO-saturated 100 mM CHES buffer, pH 8.0, and allowed to incubate for 30 min. 300 mM sucrose was added as a glassing agent, and 140 µL of 1 mM protein was then transferred into a Delrin sample cell and frozen in liquid nitrogen. The $[NiFe_3S]$-CN Fd sample was prepared by incubating $[NiFe_3S_4]$ Fd with 4-fold molar excess DT and 30-fold molar excess KCN for 10 min. The sample was then diluted with glycerol to a final concentration of 20%, and 140 µL of 1 mM protein was loaded into a white Delrin sample holder and frozen in liquid nitrogen. The $[NiFe_3S_4]^{2+}$ Fd sample was prepared by incubating $[NiFe_3S_4]$ Fd with 20-fold molar excess thionin acetate (VWR) for 15 min. After 15 min, 0.1 molar equivalents of $KFeCN_6$ was added along with 20% glycerol and 140 µL of 1 mM protein was loaded into a white Delrin sample cell and frozen in liquid nitrogen. No $KFeCN_6$ was used in the Fe K-edge sample to prevent contaminating Fe signal. It is noting that the $[NiFe_3S_4]^{2+}$ Fd has ~30% contamination from $[Fe_3S_4]^+$ Fd, which may contribute to the Fe K-edge features (**Figure S33**).

***EPR spectroscopy*.**

Continuous-wave (CW) X-band EPR spectra were collected using a Bruker EMXPlus equipped with a ColdEdge cryogen-free helium cryostat and recirculation system and an Oxford Instruments MercuryITC temperature controller. All presented spectra were obtained using a microwave frequency of 9.37 GHz and a modulation frequency and amplitude of 100 kHz and 10 G, respectively. Background signals were removed by baseline subtraction using IGOR Pro 9.00 (Wavemetrics, Lake Oswego, OR).

Power- and temperature-dependent experiments were performed by adjusting the $P_{\mu w}$ in a range from 63.25 - 0.0003991 mW at the indicated temperatures. Spectra were then baseline-corrected, and the intensities were tabulated. For the Curie-corrected data, the intensities were multiplied by the temperature at which the spectrum was collected ([I x T]). $P_{1/2}$ values were obtained by fitting the following equation to the power saturation curves.

$$S(signal) = \frac{C\sqrt{P}}{\sqrt{1 + \frac{P}{P_{1/2}}}} \qquad (1)$$

Quantification of the EPR line broadening of ^{61}Ni and ^{57}Fe isotopically labelled $NiFd_{red}$, NiFd-CN, NiFd-CO was performed by considering the difference in the FWHM of the indicated spectral signatures. The maximum intensity of the feature was determined (**Δ**y), then the width of the peak (**Δ**x) at ½ of the maximum intensity (**Δ**$y_{1/2}$) was measured to determine the FWHM of the peak (**Figure S31**). The

differences in the FWHM upon isotopic enrichment with ^{61}Ni or ^{57}Fe reflect the line broadening due to the electronic hyperfine coupling.

FTIR spectroscopy.

All FTIR samples were prepared in a nitrogen atmosphere glovebox. A 1 mM $[NiFe_3S_4]^+$-CO Fd sample was prepared by incubating a solution of freshly desalted and concentrated $[NiFe_3S_4]$ Fd with 4 mM DT while stirring under a headspace of CO in 100 mM HEPES (GoldBio), pH 7.0. After 30 min, 60 μL of sample was transferred into a sealed, temperature-controlled FTIR cell with CaF_2 windows (Sigma-Aldrich, ThermoFisher Scientific), using a .05 mm Teflon spacer. The sample was kept at 10 ºC using an IKA RC2 recirculating chiller. Spectra were averaged over 200 scans using a PerkinElmer Spectrum 65 FTIR-Spectrometer with 2 cm^{-1} resolution.

Resonance Raman spectroscopy.

Resonance Raman spectra were collected using a setup described previously.[3] All resonance Raman spectra were collected at 77 K using samples within a liquid nitrogen finger dewar with excess dithionite present to prevent cluster oxidation. The optical spectra suggested both 406.7 nm and 458 nm would provide resonance enhancement of the cluster modes across all NiFd species, though, experimentally, substantially lower enhancement was observed using an excitation wavelength of 458 nm (**Figures S17-S19**). For the spectra collected using a 407 nm excitation wavelength, a tunable Titanium:Sapphire (Spectra-Physics Tsunami) laser pumped by a 25 W DPSS laser (Spectra-Physics Millenia eV) and configured with a 10 ps Gires-Tournois interferometer was used to generate the 814 nm fundamental beam. Using a β-Barium Borate (BBO) crystal (Eksma Optics), the frequency was doubled to generate the 407 nm beam. For spectra collected using 458 nm excitiation wavelength, a 458 nm excitation beam was generated using a mixed gas Kr-Ar laser (Coherent Innova Spectrum 70-C, Laser Innovations). The excitation beam was then focused onto the sample using a 100 mm focal length UV plano-convex lens (Thorlabs), and the scattered light was collected using a UV-fused aspheric lens (Edmund Physics). Elastic scattering of the Rayleigh line was rejected using the corresponding long-pass edge filter (Semrock RazorEdge), and the Raman scattered light was imaged onto a spectrograph (Princeton Instruments Isoplane) furnished with an 1800 gr/mm holographic grating (407 nm excitation) or a 1200 gr/mm, 500-nm blazed grating (458 nm excitation) and measured with a CCD detector (Princeton Instruments Pixis 100B). Raman signal intensity was optimized and calibrated using known bands from a 1:1 v/v mixture of toluene and acetonitrile.[4] All spectra at 407 nm were collected using P_{407} = 8 mW and represent the sum of 180 one-minute exposures. All spectra at 458 nm were collected using P_{458} = 15 mW and represent the sum of 120 one-minute exposures. For both excitation wavelengths, buffer and quartz were subtracted from the spectra, and broad baselines were removed using spline subtraction using IGOR Pro 9.00

Ni K-edge XAS Spectroscopy.

All samples were run at the Stanford Synchroton Radiation Laboratory (SSRL) 9-3 beamline with samples maintained at 10 K for the duration of the data collection using an Oxford Instruments lHe cryostat. A Si(220) double-crystal monochromator was used to select for light monochromatization. The Ni Kα fluorescence data were collected using a 100-element solid-state Ge detector array (Canberra) with a 6 μm cobalt metal filter placed between the sample and the detector. Energy calibrations were performed by simultaneously measuring nickel foil with the first inflection point set to 8333.0 eV. Data were collected in 5 eV steps from 8050.0 – 8315.0 eV (1 s integration time), 0.2 eV steps from 8315.0 – 8359.8 eV (1 s integration time), 8359.8 eV – k = 14 Å^{-1} for steps of 0.05 Å^{-1}. All channels were individually inspected prior to averaging using Athena (Version 0.9.26).[5] Edge positions were determined by finding the energy value at half the intensity of the maximum white line intensity.[6]

Ni K-edge EXAFS Spectroscopy.

Upon averaging of the XAS spectrum, the data set was imported into the Artemis software for fitting of the spectra.[5] Data sets were cut off at k = 12 Å^{-1} to remove artifacts from the data that were present in the region

of k = 12 – 14 Å^{-1}. Spectra were fit in k-space from k = 2 – 12 Å^{-1}, and initial oscillation patterns were found by performing a feff calculation within the Artemis software by importing the DFT-optimized structures of the given species.[7] Best-fit values to the experimental data are presented in **Figure 4** and values in **Table 1**. Given the resolution of 0.130 Å afforded by fitting the data to k = 12 Å^{-1}, we cannot definitively quantify the changes in bond length of less than the resolution. An in-depth discussion of alternative EXAFS fits is given in the Supporting Information (**Tables S1-S6**). Contributions of each scattering pathway for all NiFd species are displayed in **Figures S20-S23**.

***Fe K-edge XANES Spectroscopy*.**

All samples were run at the Stanford (SSRL) 9-3 beamline with samples maintained at 10 K for the duration of the data collection using an Oxford Instruments lHe cryostat. A Si(220) double-crystal monochromator was used to select for light monochromatization. The Fe Kα fluorescence data were collected using a 100-element solid-state Ge detector array (Canberra) with a 3 μm manganese metal filter placed between the sample and the detector. Energy calibrations were performed by simultaneously measuring iron foil with the first inflection point set to 7111.3 eV. Data were collected in 10 eV steps from 6830 - 7095 eV (1 s integration time), 0.2 eV steps from 7095 – 7139.8 eV (1s integration time), and 4.5 eV steps from 7139.8 – 7405.3 eV (0.5 s integration time). All channels were individually inspected prior to averaging using the Athena software.[5] Edge positions were determined by finding the energy value at half the intensity of the maximum white line intensity.[6]

Density Functional Theory Geometry Optimizations.

The large cluster models of the active site were constructed as previously described,[3] including the full secondary sphere residues and truncating the amino acid side chains with N-terminal acetylation and C-terminal amidation. Detailed explanation into the initial construction of all models can be found in the Supporting Information. The quantum chemistry software package used for all calculations was ORCA v4.2.1.[8] For the DFT calculations, a hybrid TPSSh functional was used, along with zeroth-order regular approximation (ZORA) to account for the relativistic approximation employing def2 and the D3 dispersion corrections.[9,10] Def2-TZVP(-f) basis sets were used with RIJCOSX approximation to speed up calculations of the Coulombic and exchange integrals.[11] Tight SCF, SlowConv criteria, and increased integration grids (grid4 and gridX4) were applied. Positions of hydrogen atoms were optimized followed by the geometry optimization of the whole active site in the high-spin state with Cartesian constraints applied to the α-carbons on all four of the cluster ligands (Cys11, Asp14, Cys17, Cys56). TD-DFT calculations were carried out using a hybrid TPSSh functional with the ZORA-def2-TZVP(-f) basis set. An expanded core potential set CP(PPP) and increased integration grids (grid4 and gridX4) were applied to nickel for Ni K-edge spectra. Electronic structure properties were assessed through analysis of the frontier molecular orbitals (FMOs) and evaluation of the Löwdin and Mulliken charge and population densities.

Density Functional Theory Model Construction.

The computational models were developed starting from the crystal structure of the D14C ferredoxin mutant protein from *Pyrococcus furiosus* (PDB ID: 2Z8Q). This structure was trimmed and adapted to incorporate the first and second coordination spheres of the system. The amino acid sequence was truncated to include Cys11 to Cys17 and both Cys56 and Pro57. C-terminal amidation and N-terminal acetylation were used at each terminus to retain charge neutrality but still mimic the peptide bond. No change was made to the remaining residues other than substitution of Cys14 for an aspartate. Lastly, the Fe-coordinated by residue 14 was substituted for a Ni in the metal cluster. Constraints on the 4 alpha-carbons for the four metal-binding protein ligands were used preemptively to keep the cluster intact.

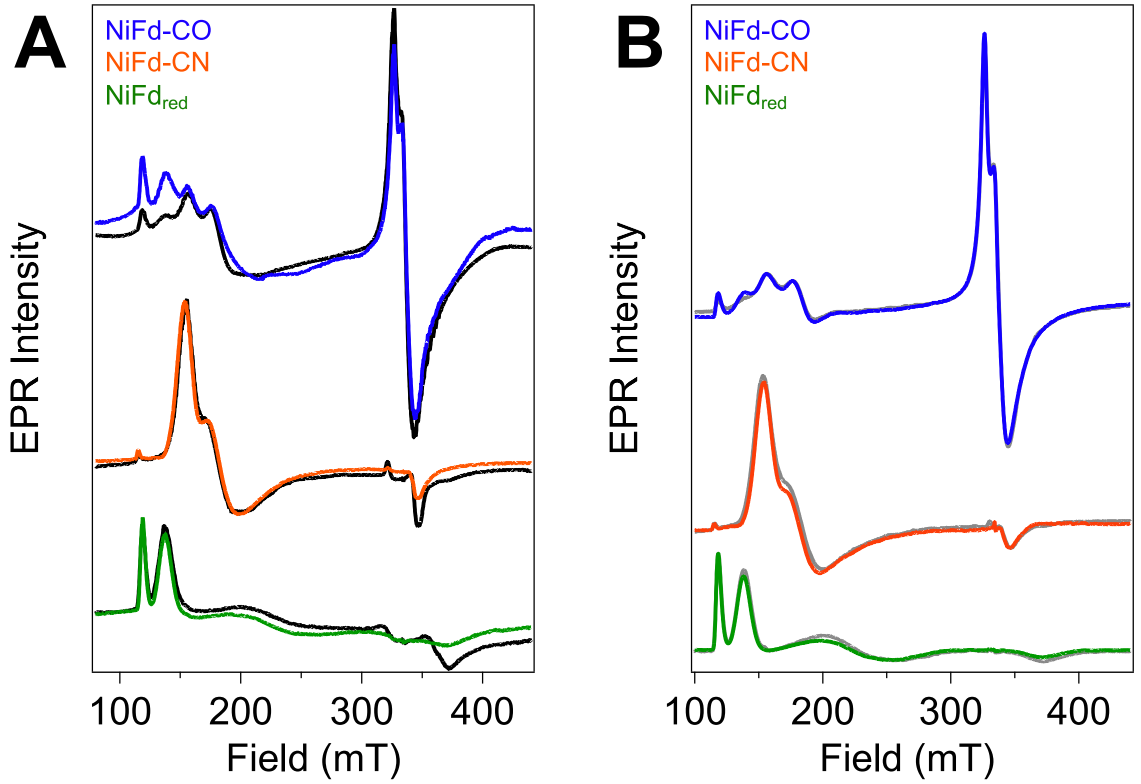

Figure S1. CW EPR spectra (ν = 9.37 GHz, T = 8 K, $P_{\mu w}$ = 20 mW) of (**A**) ^{57}Fe-labelled (*black*) and (**B**) ^{61}Ni-labelled (*gray*) NiFd samples overlaid with natural-abundance Fe (^{NA}Fe) and Ni (^{NA}Ni) samples (colored traces as indicated in legend).

Discussion of ^{57}Fe-labelled NiFd species

In conjunction with ^{61}Ni-labelling, we also performed ^{57}Fe-labelling for the three NiFd species. Only small changes in linewidth were observed, indicative of delocalized hyperfine coupling between the I = ½ nucleus of ^{57}Fe to the unpaired electrons in the cluster. Specifically, NiFd$_{red}$ exhibits 0.6 mT line broadening at the g = 5.7 feature upon incorporation of ^{57}Fe. Interestingly, the NiFd-CN does not exhibit any significant broadening (< 0.1 mT) of the g = 3.65 feature; however, incorporation of ^{57}Fe into NiFd-CO results in a broadening of 1.5 mT and 0.8 mT at the g = 3.65 and g = 2.05 features, respectively. The line broadening values observed by ^{57}Fe incorporation are not precise enough to determine any hyperfine values. To this end, Mössbauer and high-field pulsed EPR experiments are currently underway to determine the hyperfine values of the ^{57}Fe nuclei and will be the subject of a future report.

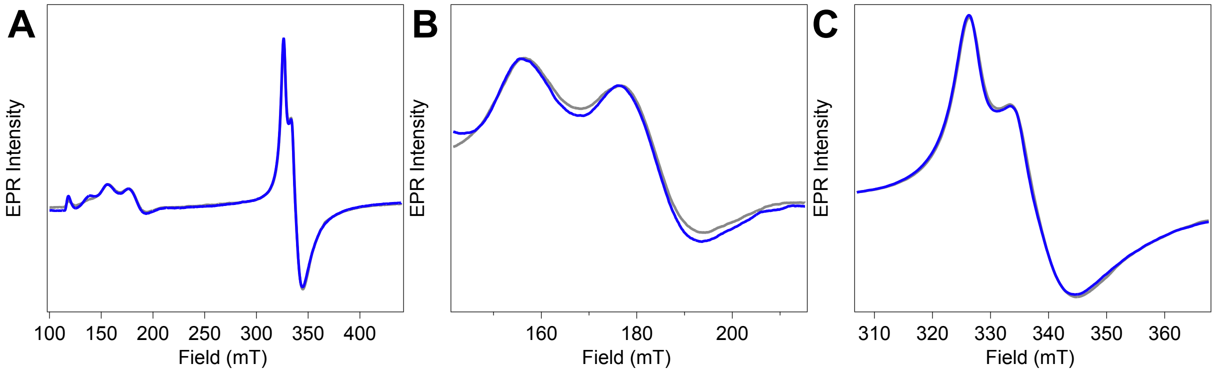

Figure S2. CW EPR Spectra (ν = 9.37 GHz, T = 8 K, $P_{\mu w}$ = 20 mW) of (**A**) ^{61}Ni-labelled (*gray*) and natural abundance Ni (^{NA}Ni) NiFd-CO (*blue*). (**B**) Zoomed-in view of low-field turning point. (**C**) Zoomed-in view of high-field turning point.

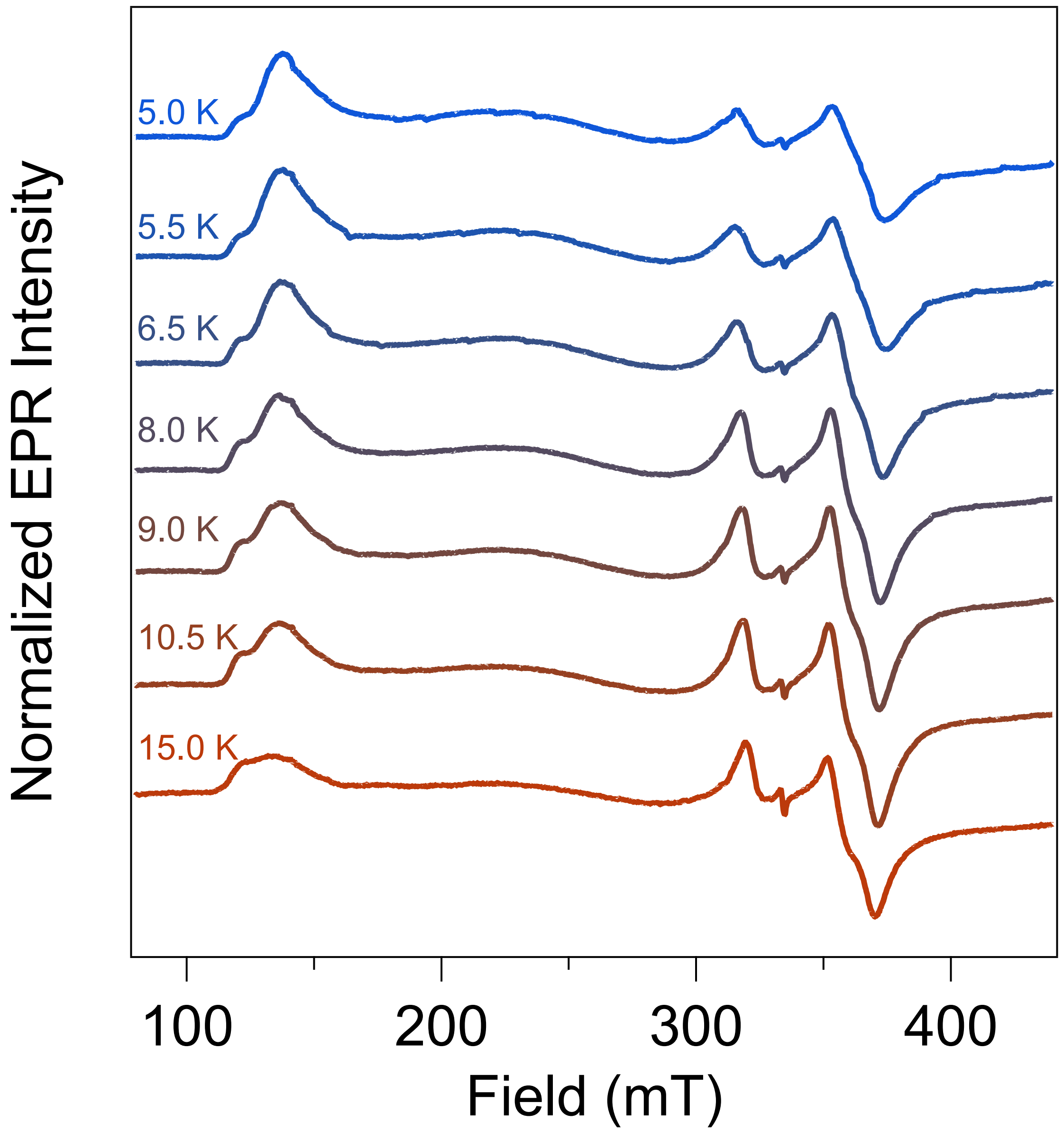

Figure S3. CW EPR spectra (ν = 9.37 GHz, $P_{\mu w}$ = 20 mW) of $[Fe_4S_4]^+$ Fd at the indicated temperatures. Spectra were normalized to temperature using the typical Curie relationship [I x T].

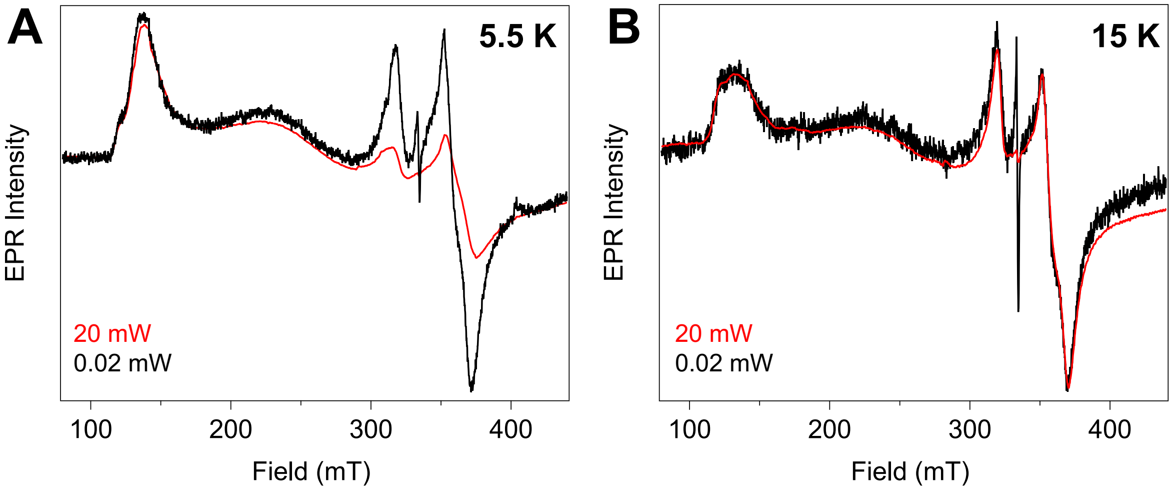

Figure S4. CW-EPR spectra (ν = 9.37 GHz) of $[Fe_4S_4]^+$ Fd at (**A**) T = 5.5 K and (**B**) 15 K at indicated powers. Spectra are scaled to correct for differences in microwave power.

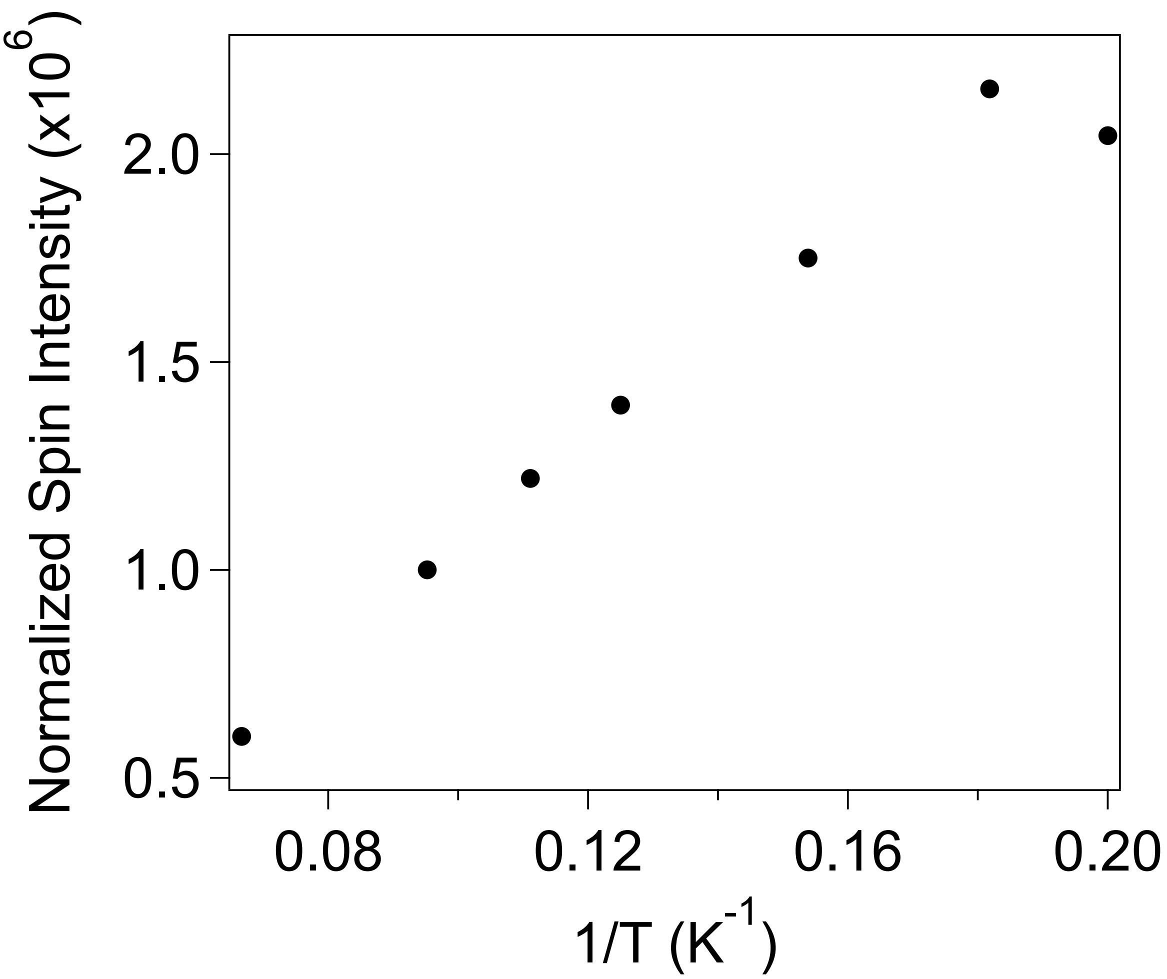

Figure S5. CW EPR spin-integrated intensity of $[Fe_4S_4]^+$ Fd at various temperatures ($P_{\mu w}$ = 2 mW).

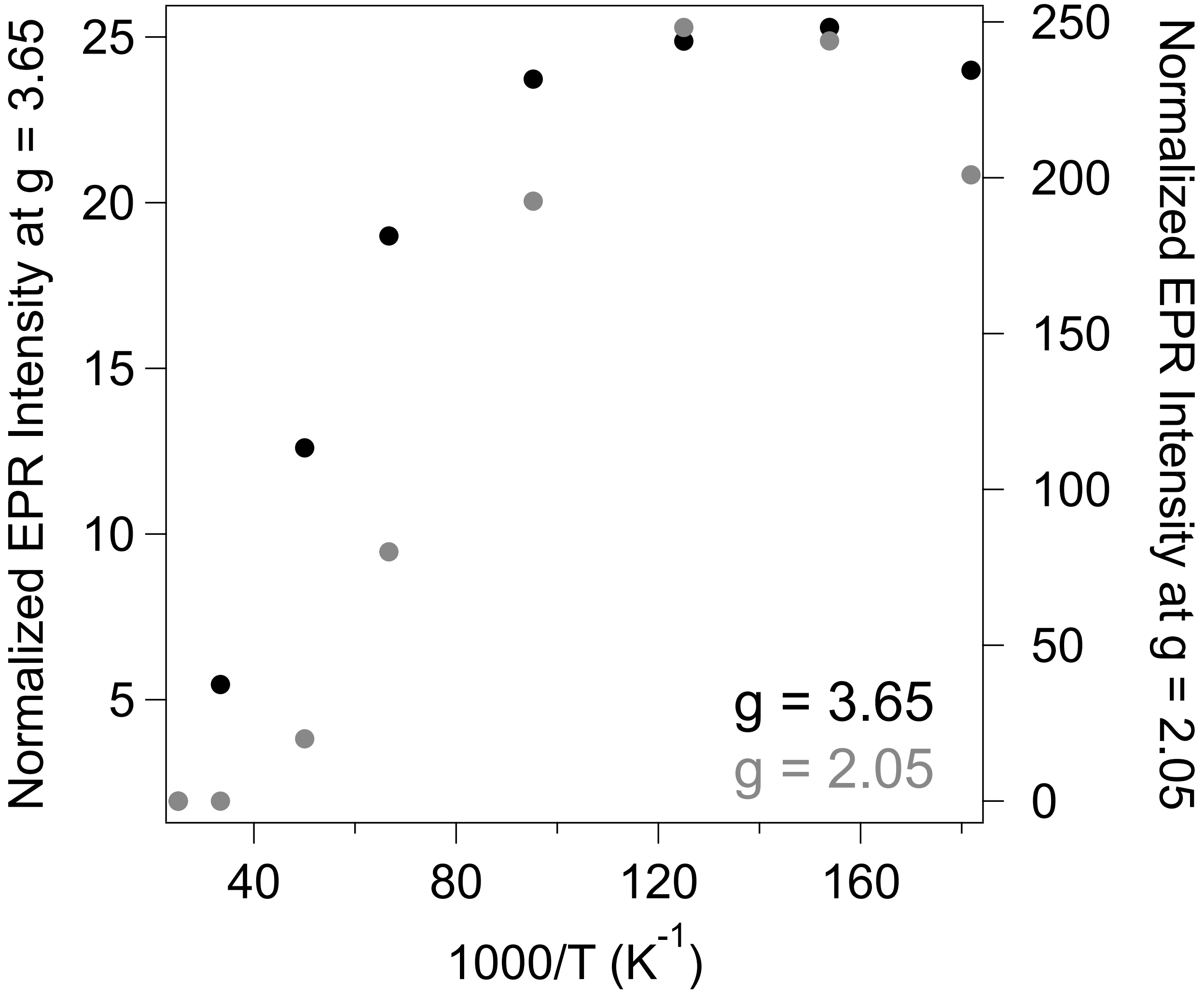

Figure S6. Curie plot of NiFd-CO CW EPR intensities ($P_{\mu w}$ = 20 mW) monitored using the features at g = 3.65 (*black*) and 2.05 (*gray*).

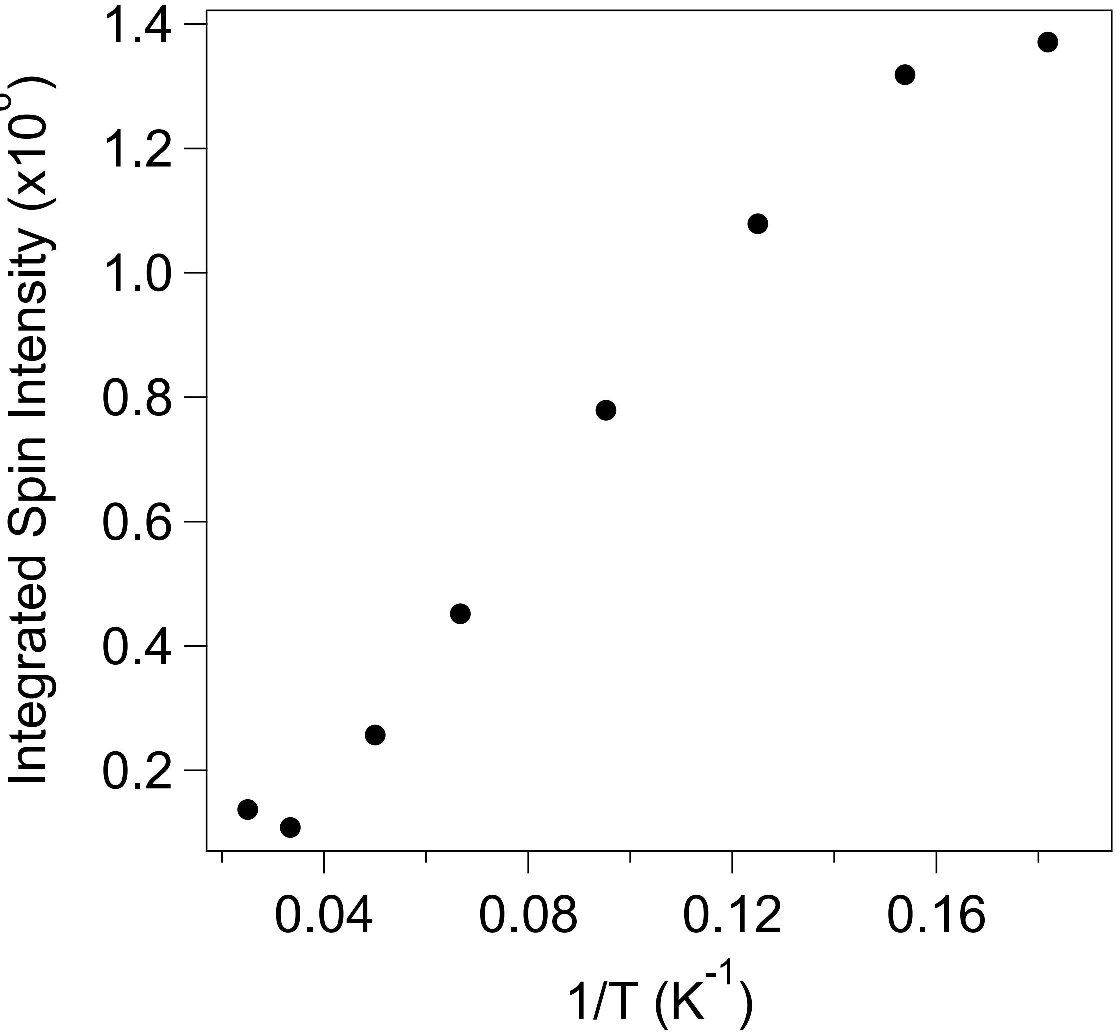

Figure S7. CW EPR spin integrated intensity of NiFd-CO at various temperatures ($P_{\mu w}$ = 20 mW).

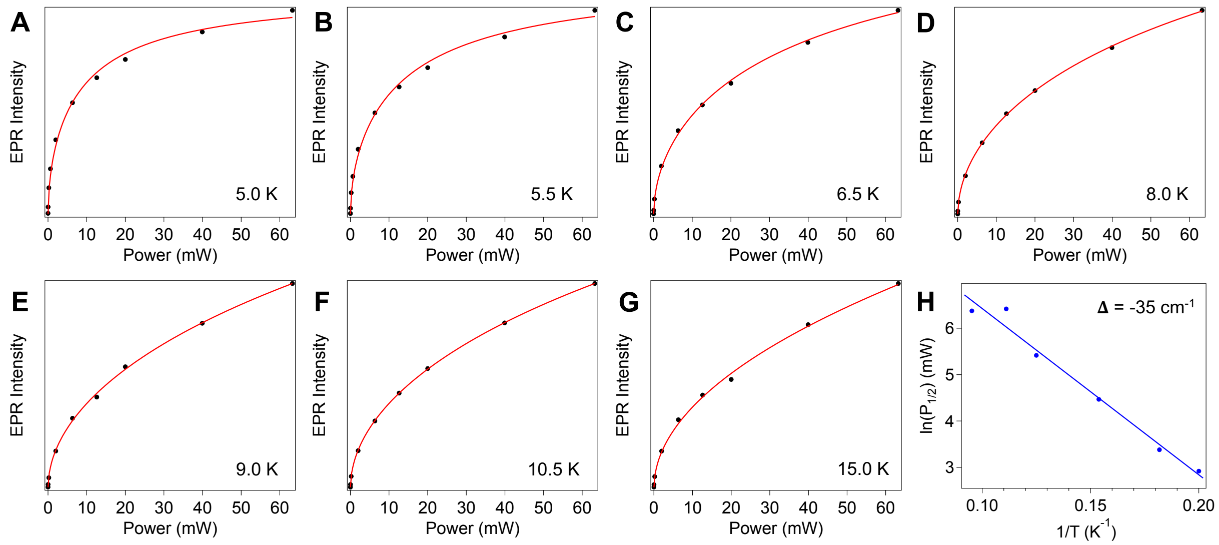

Figure S8. Temperature- and power-dependent EPR signal intensities of the g = 2.05 feature of NiFd-CO. (**A-G**) Power saturation curves were fit to Eq. 1 (*main text*) to obtain $P_{1/2}$ at each temperature. (**H**) A linear fit of $\ln(P_{1/2})$ as a function of inverse temperature yields the energy spacing **Δ**.

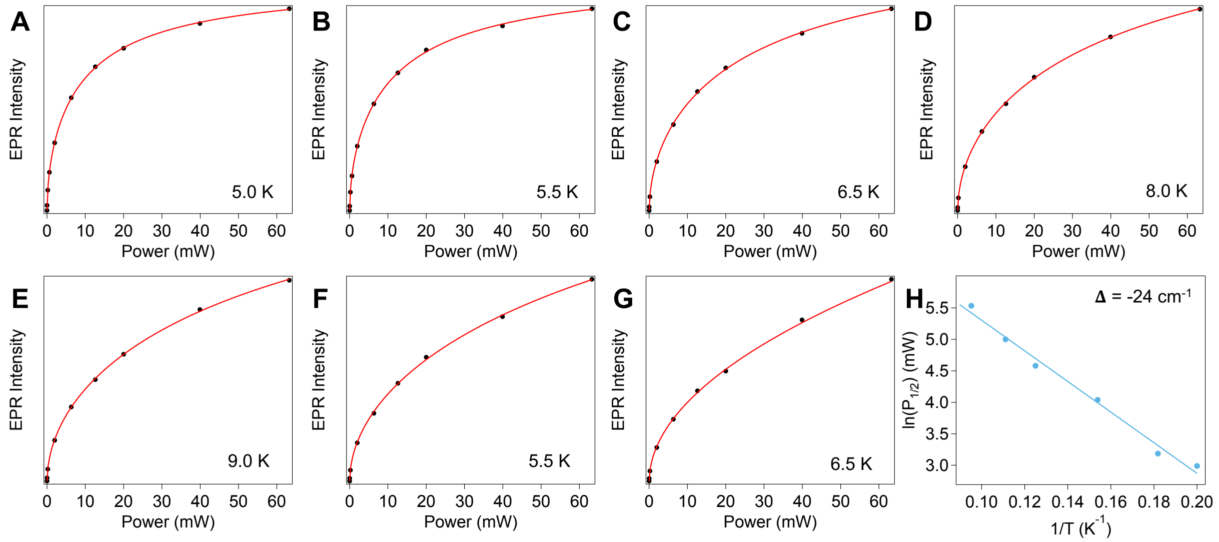

Figure S9. Temperature- and power-dependent EPR signal intensities of the g = 3.65 feature of NiFd-CO. (**A-G**) Power saturation curves were fit to Eq. 1 (*main text*) to obtain $P_{1/2}$ at each temperature. (**H**) A linear fit of ln($P_{1/2}$) as a function of inverse temperature yields the energy spacing **Δ**.

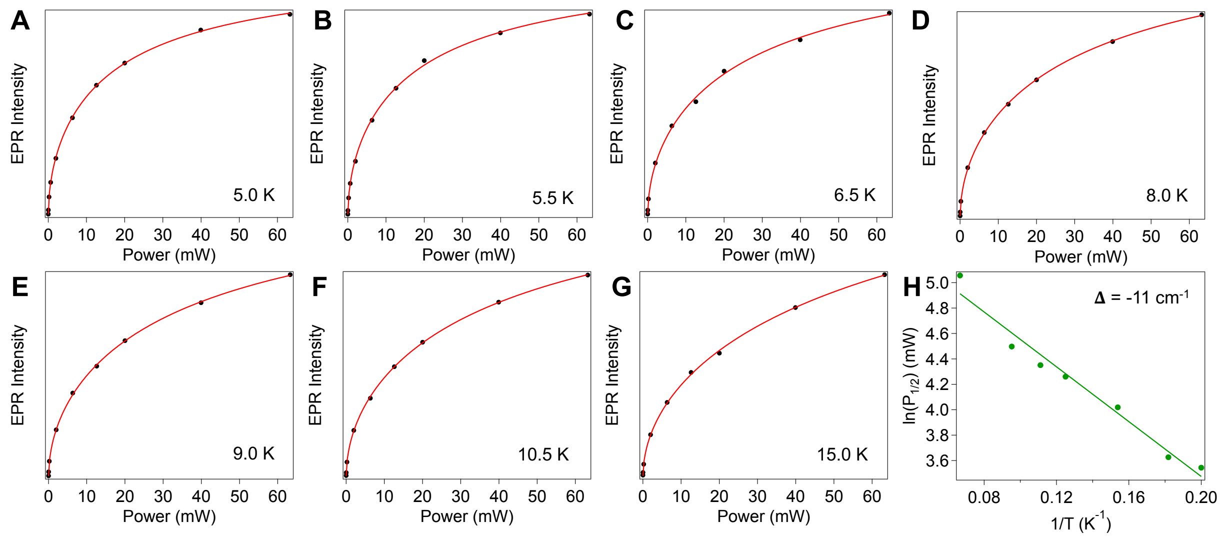

Figure S10. Temperature- and power-dependent EPR signal intensities of the g = 5.6 feature of $NiFd_{red}$. (**A-G**) Power saturation curves were fit to Eq. 1 (*main text*) to obtain $P_{1/2}$ at each temperature. (**H**) A linear fit of $\ln(P_{1/2})$ as a function of inverse temperature yields the energy spacing **Δ**.

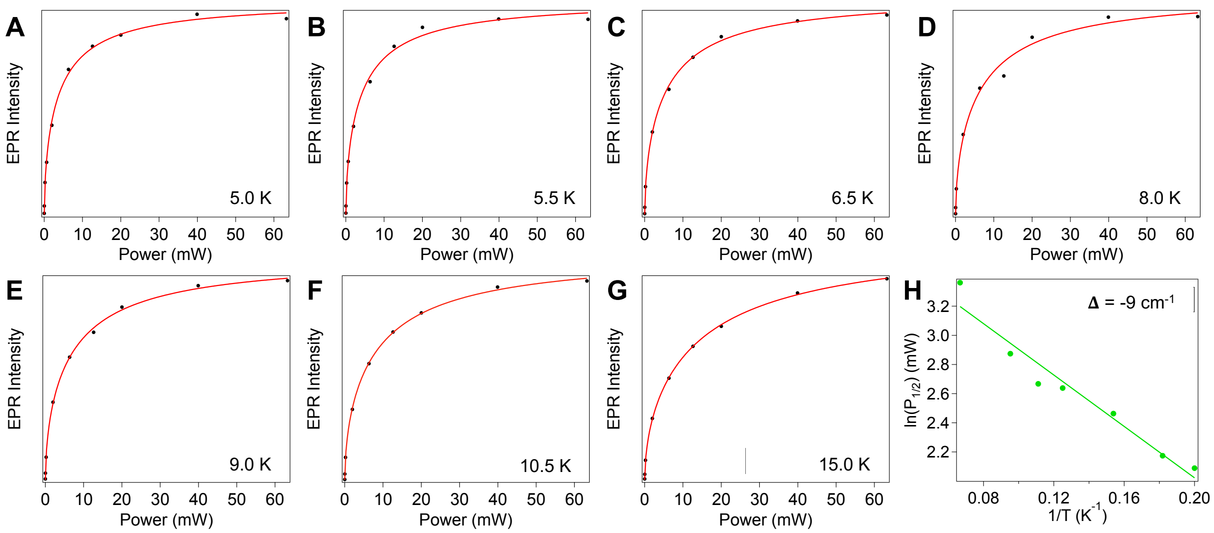

Figure S11. Temperature- and power-dependent EPR signal intensities of the g = 5.0 feature of $NiFd_{red}$. (**A-G**) Power saturation curves were fit to Eq. 1 (*main text*) to obtain $P_{1/2}$ at each temperature. (**H**) A linear fit of $\ln(P_{1/2})$ as a function of inverse temperature yields the energy spacing **Δ**.

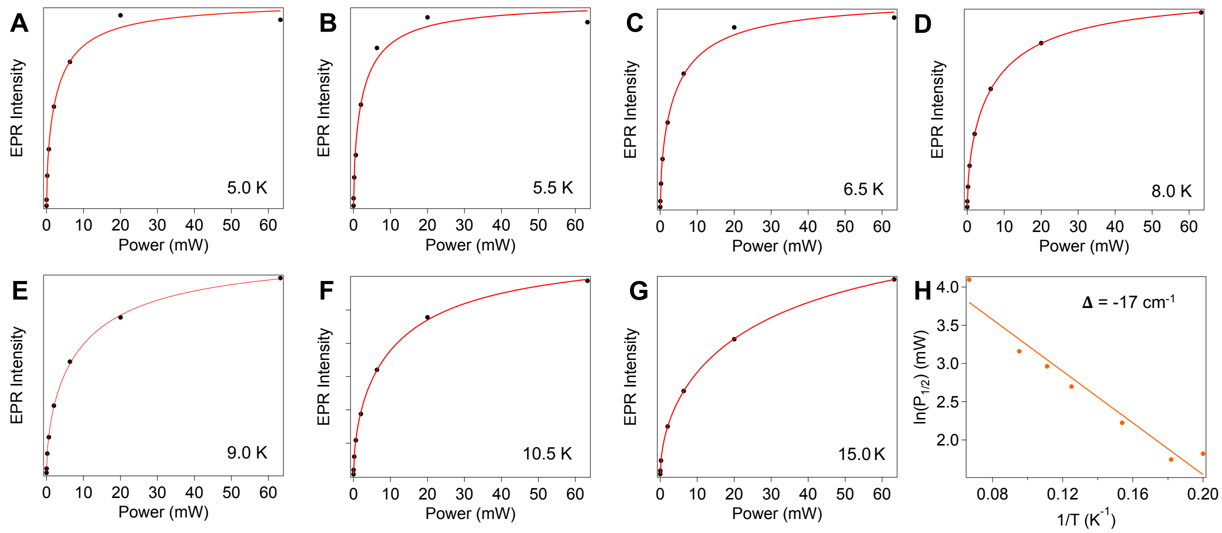

Figure S12. Temperature- and power-dependent EPR signal intensities of the g = 3.65 feature of NiFd-CN. (**A-G**) Power saturation curves were fit to Eq. 1 (*main text*) to obtain $P_{1/2}$ at each temperature. (**H**) A linear fit of ln($P_{1/2}$) as a function of inverse temperature yields the energy spacing **Δ**.

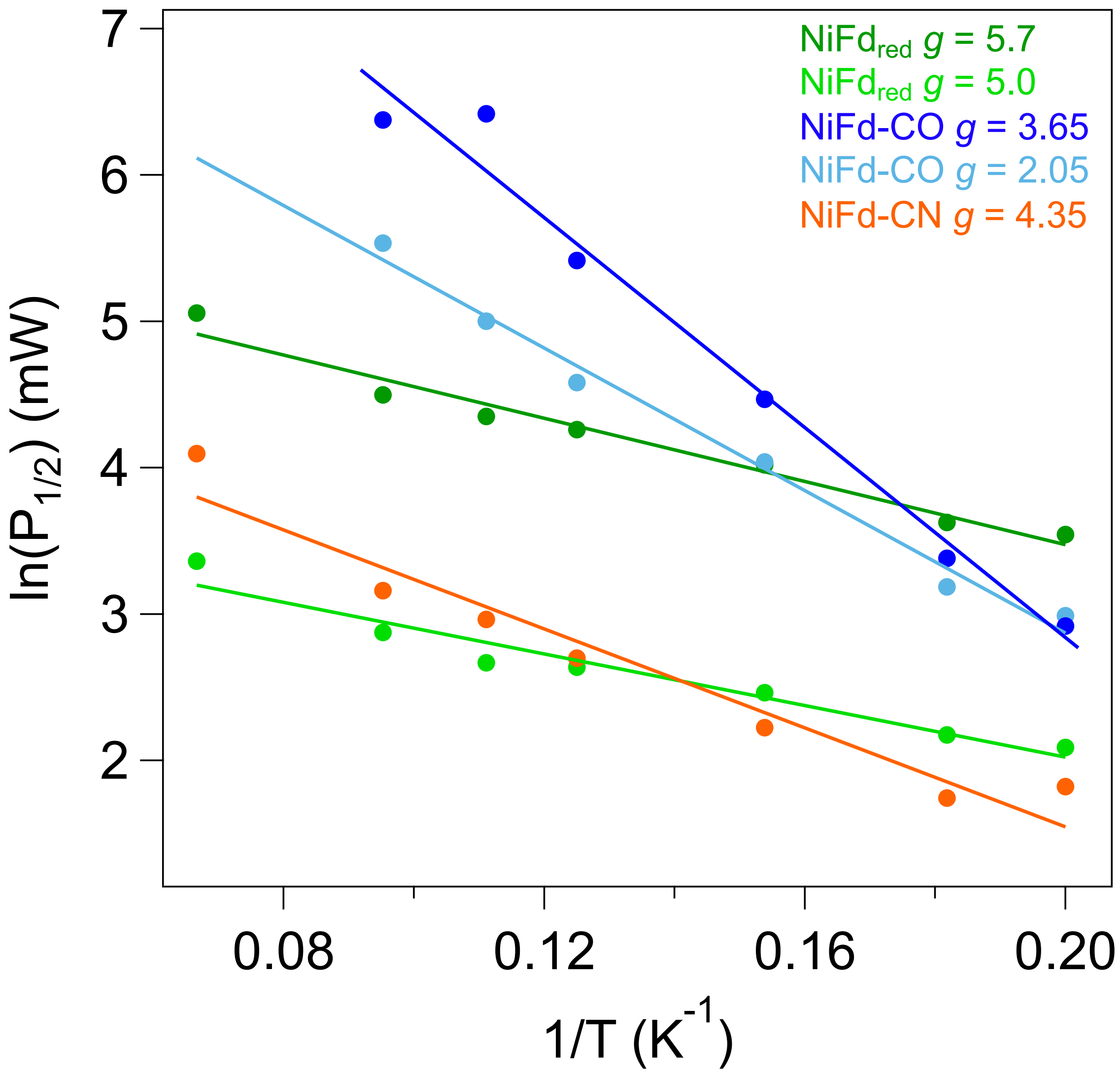

Figure S13. Orbach relaxation fits for the indicated NiFd species at the indicated g-values.

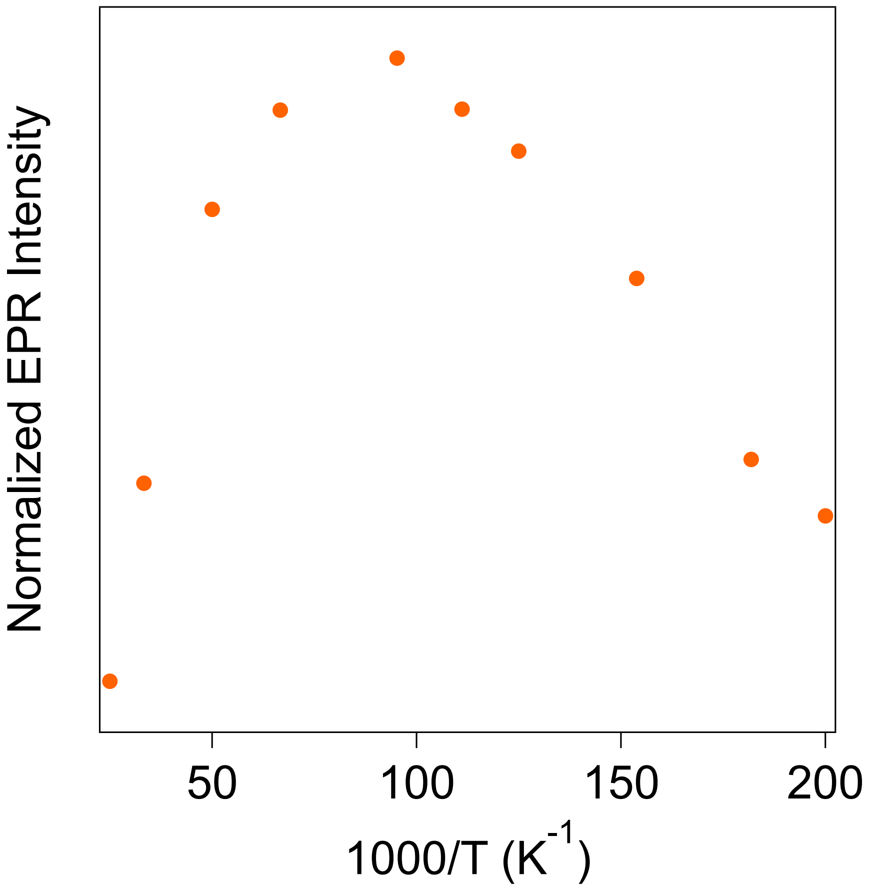

Figure S14. Curie plot of the NiFd-CN CW EPR intensities ($P_{\mu w}$ = 20 mW) monitored using the g = 3.65 feature.

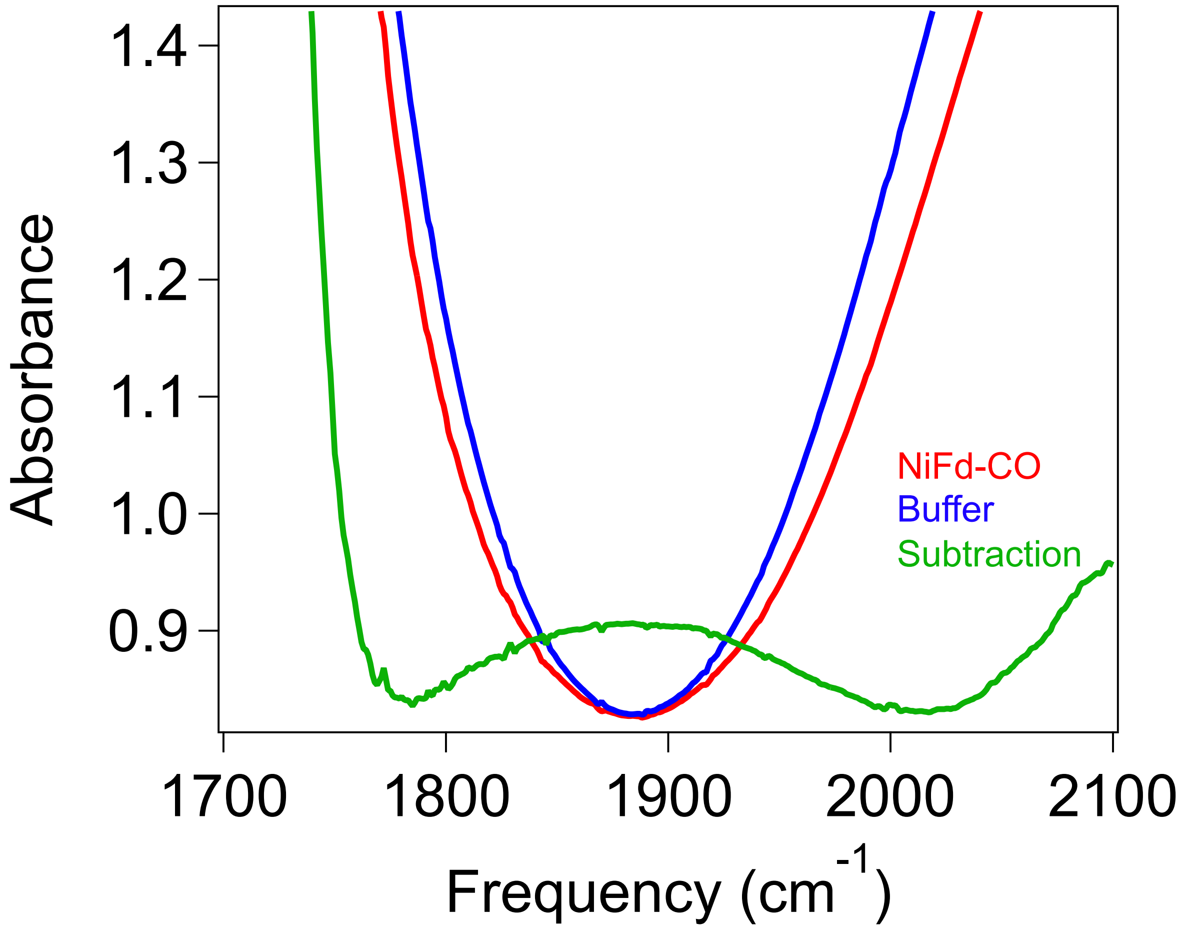

Figure S15. FTIR spectra (T = 10 ºC) of 1 mM NiFd-CO (*red*), 100 mM HEPES buffer (*blue*), and the direct subtraction. (*green*)

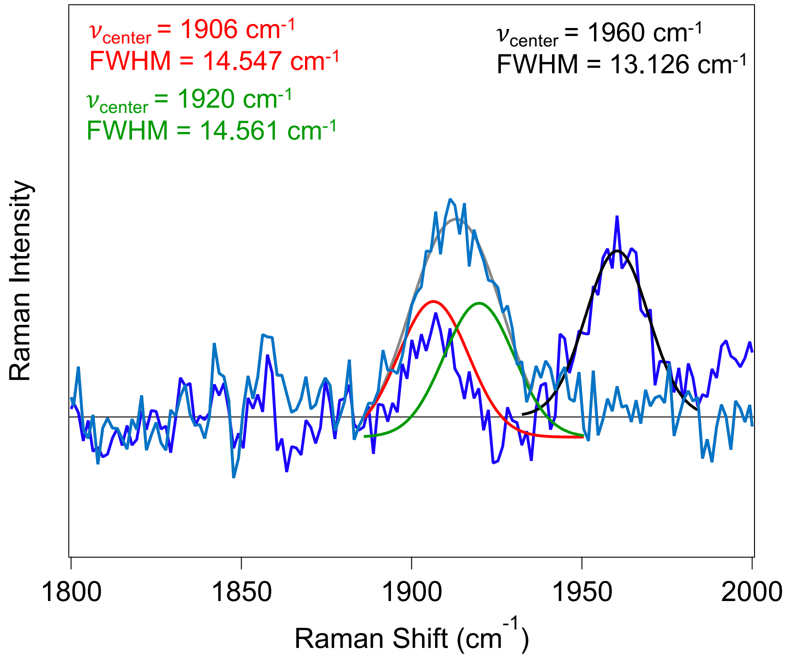

Figure S16. Gaussian fits of the high frequency region of the resonance Raman spectra of NiFd-^{NA}CO (*blue*) and NiFd-^{13}CO Fd (*light blue*) (77 K, λ_{ex} = 407 nm, P = 8 mW). Both spectra were decomposed into a sum of Gaussian curves, with the gray Gaussian curve showing the sum of the red and green Gaussian curves. The band at 1906 cm^{-1} is present in both samples and independent of the CO isotope. The zero line (horizontal black line) is included for reference.

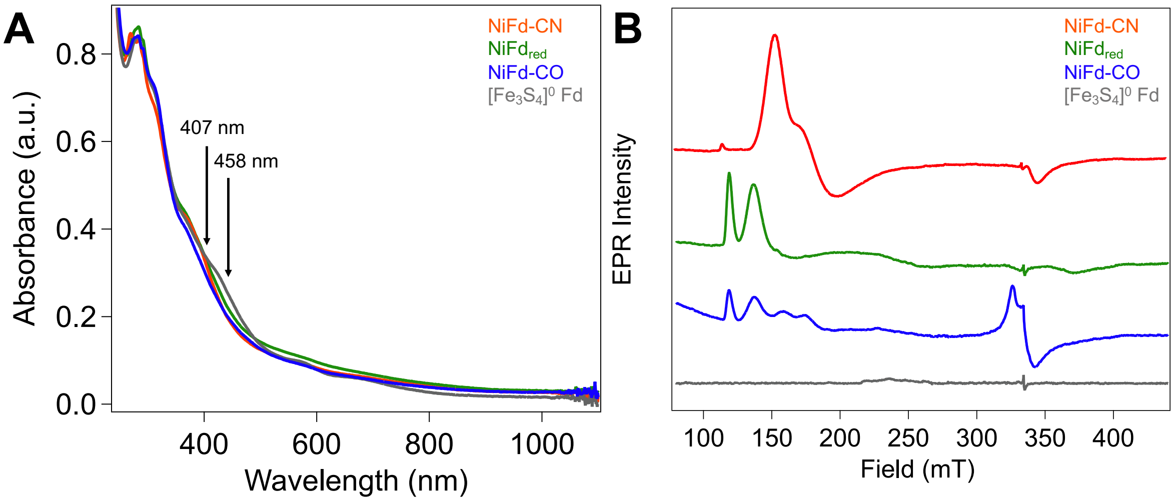

Figure S17. (**A**) Optical spectra of NiFd-CN (*orange*), NiFd$_{red}$ (*green*), NiFd-CO (*blue*), and [Fe$_3$S$_4$]^0 Fd (*gray)*. Arrows indicate excitation wavelengths selected for resonance Raman spectroscopy. (**B**) CW EPR spectra (ν = 9.37 GHz, $P_{\mu w}$ = 20 mW, T = 8.0 K) of corresponding UV-Vis samples.

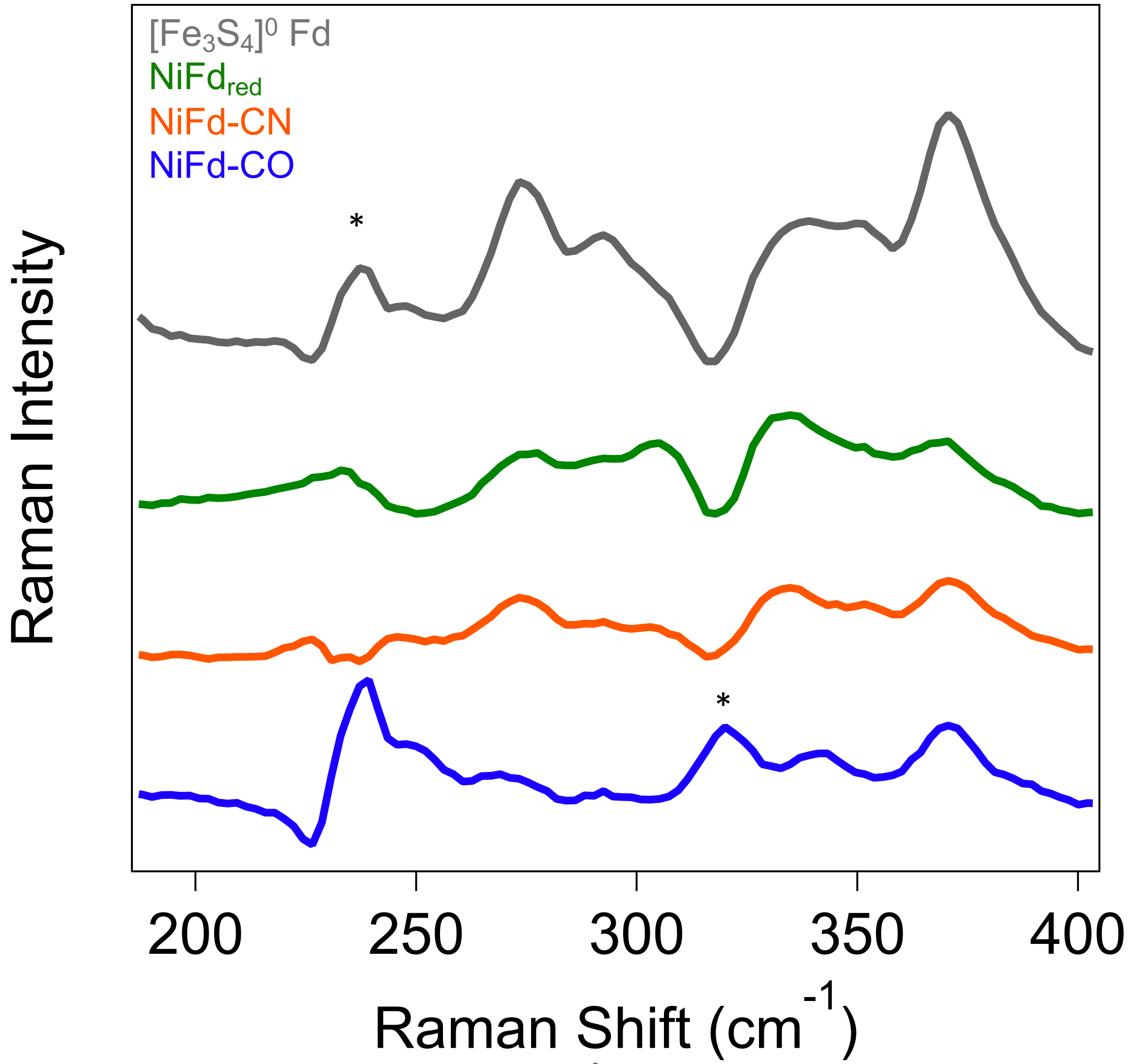

Figure S18. Resonance Raman spectra of $[Fe_3S_4]^0$ Fd (*gray*), $NiFd_{red}$ (*green*), NiFd-CN (*orange*), and NiFd-CO (*blue*) collected at 77 K using an excitation wavelength of 458 nm, P = 15 mW. Residual features corresponding to buffer are indicated with an *. Bands arising from buffer, DT, and quartz were subtracted after collection.

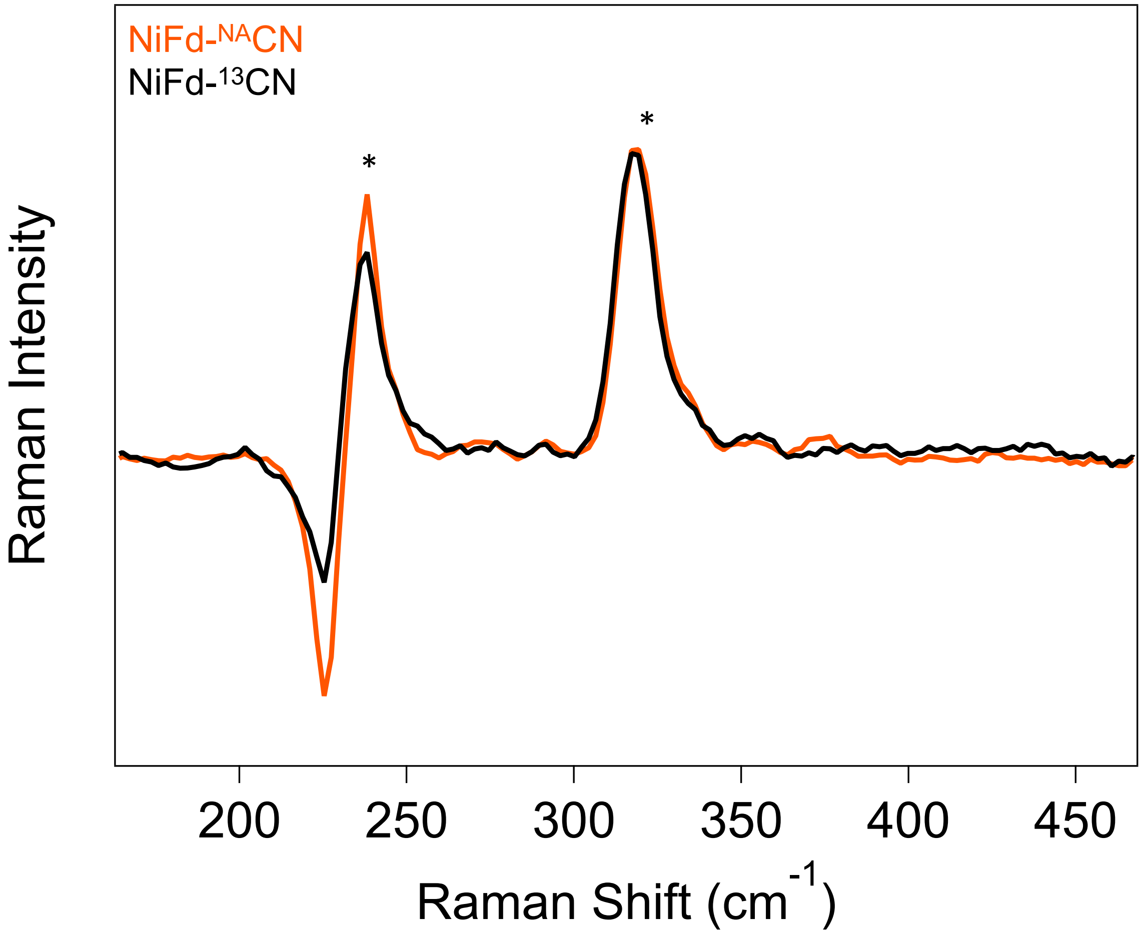

Figure S19. Resonance Raman spectra of NiFd-^{NA}CN (*orange*) and NiFd-^{13}CN (*black*), collected at 77 K using an excitation wavelength of 458 nm, P = 15 mW. Residual features corresponding to buffer are indicated with an *. Bands arising from buffer, DT, and quartz were subtracted after collection.

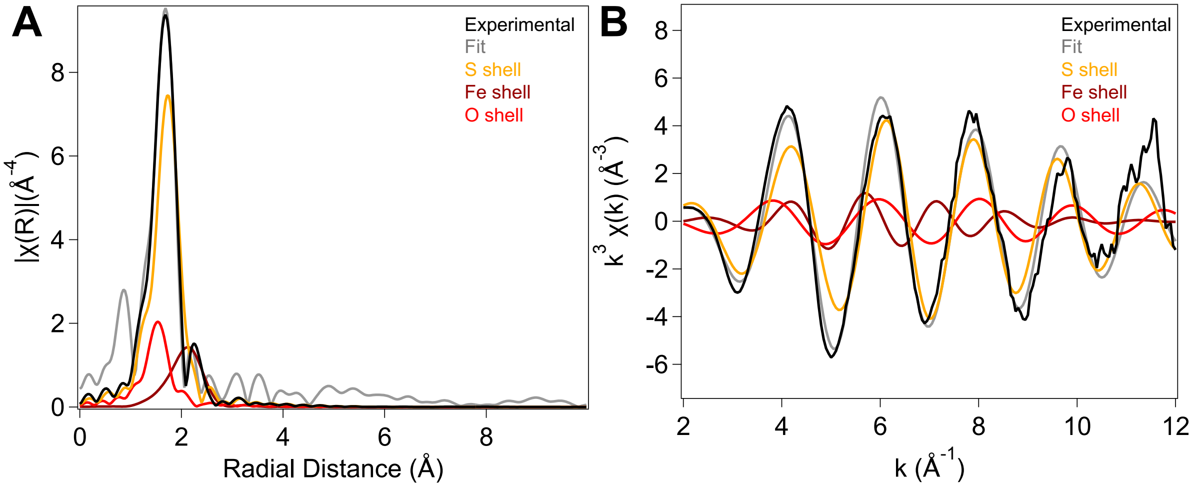

Figure S20. Ni K-edge EXAFS of the $NiFd_{ox}$ species. (**A**) Comparison of experimental Fourier transform (FT) EXAFS data (*black*) with the best fit (*gray*) and contributions from the individual scattering pathways (*colored*). (**B**) Comparison of experimental k^3 EXAFS data (*black*) with the best fit (*gray*) and contributions from the individual scattering pathways (*colored*). Fits were performed in k-space over the range from k = 2 – 12 $Å^{-1}$.

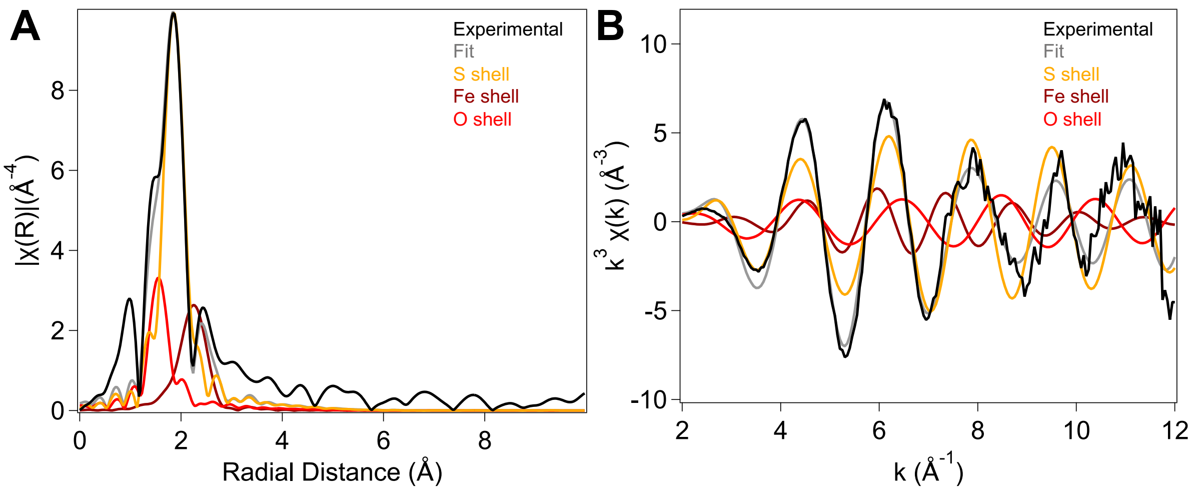

Figure S21. Ni K-edge EXAFS of the $NiFd_{red}$ species. (**A**) Comparison of experimental Fourier transform (FT) EXAFS data (*black*) with the best fit (*gray*) and contributions from the individual scattering pathways (*colored*). (**B**) Comparison of experimental k^3 EXAFS data (*black*) with the best fit (*gray*) and contributions from the individual scattering pathways (*colored*). Fits were performed in k-space over the range from k = 2 – 12 $Å^{-1}$.

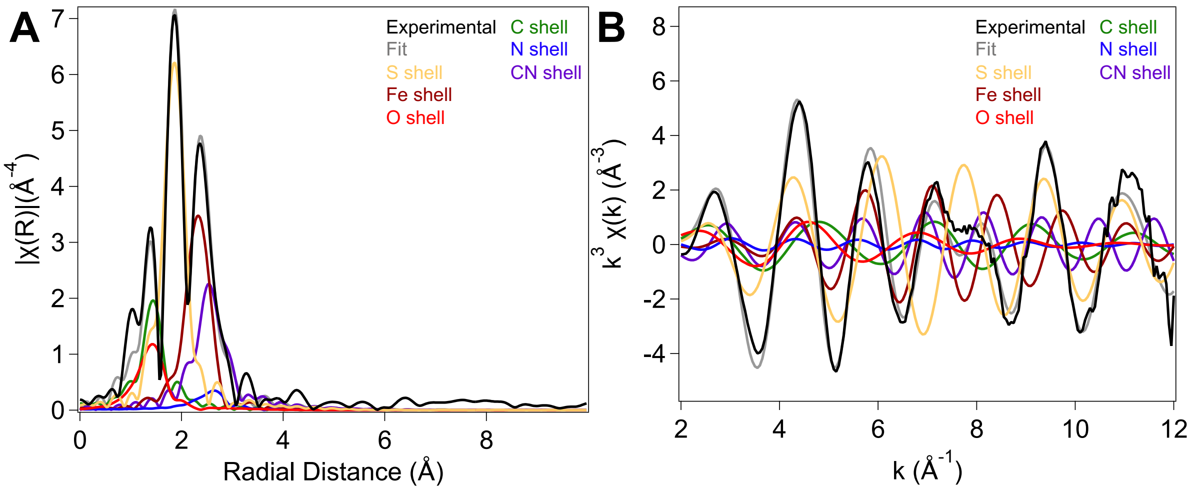

Figure S22. Ni K-edge EXAFS of the NiFd-CN species. (**A**) Comparison of experimental Fourier transform (FT) EXAFS data (*black*) with the best fit (*gray*) and contributions from the individual scattering pathways (*colored*). (**B**) Comparison of experimental k^3 EXAFS data (*black*) with the best fit (*gray*) and contributions from the individual scattering pathways (*colored*). Fits were performed in k-space over the range from k = 2 – 12 Å^{-1}.

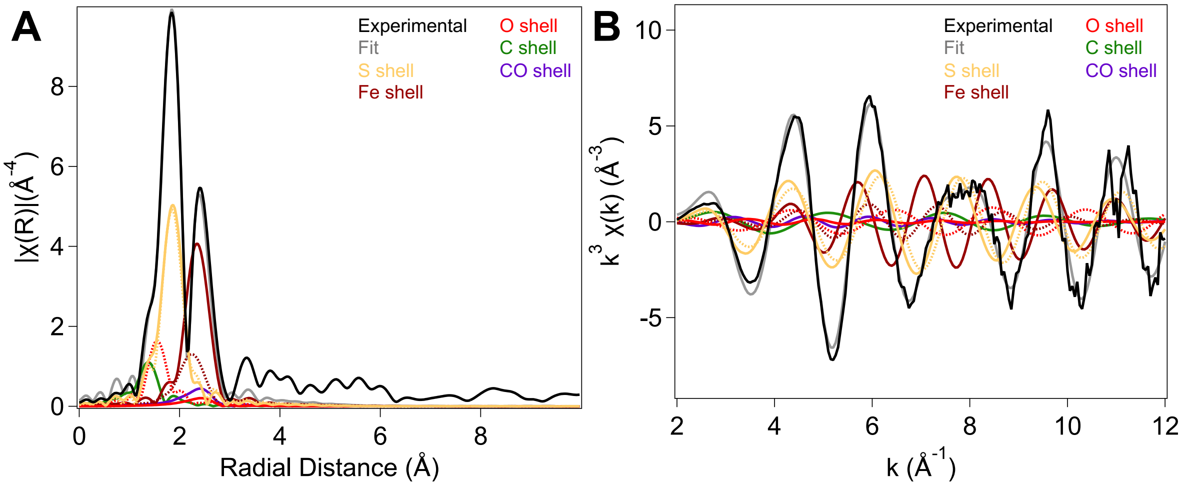

Figure S23. Ni K-edge EXAFS of the NiFd-CO species. (**A**) Comparison of experimental Fourier transform (FT) EXAFS data (*black*) with the best fit (*gray*) and contributions from the individual scattering pathways (*colored*). (**B**) Comparison of experimental k^3 EXAFS data (*black*) with the best fit (*gray*) and contributions from the individual scattering pathways (*colored*). Dashed lines indicate contributions from $NiFd_{red}$. Fits were performed in k-space over the range from k = 2 – 12 Å^{-1}.

	Reported Fit	Fit #2	Fit #3	Fit #4
Ni – O Shell				
n	1	-	1	-
r (Å)	1.95	-	1.97	-
σ^2 (Å^2)	0.00032	-	0.00022	-
Ni – S Shell				
n	3	3	2	2
r (Å)	2.27	2.26	2.26	2.20
σ^2 (Å^2)	0.00348	0.00579	0.01011	0.00329
Ni – S Shell				
n	-	-	1	1
r (Å)	-	-	2.27	2.31
σ^2 (Å^2)	-	-	0.00101	0.0000
Ni – Fe Shell				
n	3	3	2	2
r (Å)	2.65	2.65	2.68	2.71
σ^2 (Å^2)	0.01388	0.01629	0.00722	0.00607
Ni – Fe Shell				
n	-	-	1	1
r (Å)	-	-	2.51	2.55
σ^2 (Å^2)	-	-	0.00533	0.00318
ΔE_o	4.481	3.755	3.576	3.217
R – factor	0.074	0.121	0.064	0.124
Reduced χ^2	1552690	2493060	1607278	2073007

Table S1. Ni K-edge EXAFS alternative fits for the NiFd$_{ox}$ species giving number of scatters (n), interatomic distances (R) and Debye Waller factors (σ^2), delta energy (ΔE_o), R-factor, and reduced χ^2. All fits were performed in k-space in a range from k = 2 – 12 Å^{-1}.

	Reported Fit	Fit #2	Fit #3	Fit #4
Ni – O Shell				
n	1	-	1	-
r (Å)	2.01	-	1.96	-
σ^2 (Å^2)	0.00372	-	0.00288	-
Ni – S Shell				
n	3	3	2	2
r (Å)	2.21	2.19	2.21	2.14
σ^2 (Å^2)	0.00694	0.00550	0.00261	0.00408
Ni – S Shell				
n	-	-	1	1
r (Å)	-	-	2.33	2.23
σ^2 (Å^2)	-	-	0.00556	0.0000
Ni – Fe Shell				
n	3	3	2	2
r (Å)	2.64	2.58	2.80	2.57
σ^2 (Å^2)	0.02077	0.01884	0.01440	0.00730
Ni – Fe Shell				
n	-	-	1	1
r (Å)	-	-	2.61	2.75
σ^2 (Å^2)	-	-	0.00691	0.00549
**Δ**E_o	-8.72	-14.10	-6.33	-13.98
R - factor	0.054	0.062	0.050	0.049
Reduced χ^2	7349274	7376749	6036338	6628927

Table S2. Ni K-edge EXAFS alternative fits for the $NiFd_{red}$ species giving number of scatters (n), interatomic distances (R) and Debye Waller factors (σ^2), delta energy (**Δ**E_o), R-factor, and reduced χ^2. All fits were performed in k-space in a range from k = 2 – 12 Å^{-1}.

	Reported Fit	Fit #2	Fit #3	Fit #4	Fit #5	Fit #6	Fit #7	Fit #8
Ni – C Shell								
n	1	1	1	1	1	1	1	1
r (Å)	1.87	1.87	1.80	1.86	1.87	1.87	1.85	1.87
σ^2 ($Å^2$)	0.00320	0.00219	0.00234	0.00021	0.00139	0.00102	0.00274	0.00143
Ni – O Shell								
n	1	1	1	1	-	-	1	-
r (Å)	1.88	1.85	1.86	1.86	-	-	1.88	-
σ^2 ($Å^2$)	0.01247	0.01279	0.00197	0.01253	-	-	0.01004	-
Ni – S Shell								
n	2	2	3	3	2	3	2	3
r (Å)	2.29	2.29	2.30	2.30	2.29	2.30	2.30	2.31
σ^2 ($Å^2$)	0.00576	0.00554	0.00925	0.00972	0.00595	0.00967	0.00595	0.00976
Ni – Fe Shell								
n	3	2	3	2	2	3	3	3
r (Å)	2.75	2.72	2.76	2.76	2.72	2.76	2.75	2.77
σ^2 ($Å^2$)	0.01292	0.00881	0.00999	0.00897	0.00927	0.01168	0.01202	0.01076
Ni – N Shell								
n	1	1	1	1	1	1	-	-
r (Å)	3.19	3.26	3.18	3.19	3.25	3.18	-	-
σ^2 ($Å^2$)	0.00098	0.02579	0.00132	0.00084	0.00996	0.00091	-	-
Ni – CN Shell								
n	2	2	2	2	2	2	-	-
r (Å)	3.01	3.08	2.90	2.99	3.08	3.00	-	-
σ^2 ($Å^2$)	0.00892	0.00155	0.00936	0.00947	0.00219	0.01502	-	-
ΔE_o	1.989	2.073	2.197	1.929	2.721	2.953	3.363	4.877
R – factor	0.039	0.048	0.053	0.051	0.047	0.046	0.082	0.057
Reduced χ^2	2848655	2426786	2912897	2826527	3297773	3586654	4362165	4551963

Table S3. Ni K-edge EXAFS alternative fits for the NiFd-CN species giving number of scatters (n), interatomic distances (R) and Debye Waller factors (σ^2), delta energy (ΔE_o), R-factor, and reduced χ^2. All fits were performed in k-space in a range from k = 2 – 12 $Å^{-1}$.

	**Reported Fit**	**Fit #2**	**Fit #3**	**Fit #4**	**Fit #5**	**Fit #6**	**Fit #7**	**Fit #8**	**Fit #9**
Ni – C Shell									
n	1	1	1	1	1	1	1	1	1
r (Å)	1.80	1.82	1.83	1.81	1.82	1.92	1.84	1.83	1.79
σ^2 (Å^2)	0.00584	0.00604	0.00557	0.00142	0.01607	0.01579	0.00900	0.01608	0.00538
Ni – O Shell									
n	-	1	1	-	-	1	-	1	-
r (Å)	-	2.14	2.10	-	-	2.13	-	2.13	-
σ^2 (Å^2)	-	0.00791	0.0000	-	-	0.00326	-	0.00532	-
Ni – S Shell									
n	3	3	3	3	3	3	3	3	3
r (Å)	2.29	2.29	2.34	2.31	2.29	2.29	2.29	2.29	2.30
σ^2 (Å^2)	0.00635	0.00691	0.00859	0.00709	0.00581	0.00695	0.00577	0.00675	0.00625
Ni – Fe Shell									
n	3	3	3	3	3	3	3	3	3
r (Å)	2.73	2.73	2.75	2.74	2.73	2.73	2.72	2.73	2.73
σ^2 (Å^2)	0.00747	0.00736	0.00696	0.00604	0.00838	0.00720	0.00793	0.00775	0.00678
Ni – O Shell									
n	1	1	1	1	1	1	-	-	-
r (Å)	3.05	3.01	3.01	3.01	3.03	2.83	-	-	-
σ^2 (Å^2)	0.00930	0.01420	0.01378	0.01378	0.01102	0.0023	-	-	-
Ni – CO Shell									
n	2	2	2	2	2	2	-	-	-
r (Å)	2.99	2.99	2.99	2.96	3.00	2.97	-	-	-
σ^2 (Å^2)	0.00039	0.00087	0.00034	0.00076	0.00067	0.00067	-	-	-
% contribution	60	60	50	50	70	70	70	70	60
ΔE_o	3.160	3.445	5.123	3.242	3.248	4.600	3.166	4.601	3.862
R – factor	0.042	0.034	0.0329	0.047	0.038	0.042	0.069	0.041	0.062
Reduced χ^2	1001669	984983	938802	1067175	1115997	1038178	1222675	1218096	1185356

Table S4. Ni K-edge EXAFS alternative fits for the NiFd-CO species giving number of scatters (n), interatomic distances (R) and Debye Waller factors (σ^2), delta energy (ΔE_o), R-factor, and reduced χ^2. All fits were performed in k-space in a range from k = 2 – 12 Å^{-1}.

	Fit #1	Fit #2	Fit #3	Fit #4	Fit #5	Fit #6	Fit #7	Fit #8
Ni – C/N/O Shell								
n	2	1	2	2	1	2	1	2
r (Å)	1.84	1.84	1.85	1.84	1.84	1.84	1.84	1.85
σ^2 (Å^2)	0.00629	0.00020	0.00710	0.00552	0.00139	0.00556	0.00036	0.00648
Ni – S Shell								
n	2	3	2	3	2	3	3	2
r (Å)	2.29	2.31	2.30	2.30	2.30	2.30	2.31	2.30
σ^2 (Å^2)	0.00546	0.00955	0.00539	0.00880	0.00602	0.00892	0.00977	0.00569
Ni – Fe Shell								
n	3	3	2	3	2	3	3	3
r (Å)	2.73	2.76	2.73	2.75	2.73	2.76	2.77	2.75
σ^2 (Å^2)	0.01279	0.01118	0.00833	0.01126	0.00942	0.01094	0.01091	0.01140
Ni – N Shell								
n	1	1	1	1	1	-	-	-
r (Å)	3.14	3.19	3.15	3.17	3.15	-	-	-
σ^2 (Å^2)	0.00098	0.0000	0.02579	0.00132	0.00996	-	-	-
Ni – CN Shell								
n	2	2	2	2	2	-	-	-
r (Å)	3.09	3.15	3.11	3.16	3.09	-	-	-
σ^2 (Å^2)	0.00892	0.07961	0.00155	0.09741	0.00219	-	-	-
ΔE_o	1.943	4.391	3.542	2.197	3.384	3.729	5.221	3.275
R – factor	0.047	0.030	0.052	0.099	0.025	0.130	0.042	0.097
Reduced χ^2	1992880	1791549	2361374	3027915	1858559	4329991	2565013	6628283

Table S5. Ni K-edge EXAFS alternative fits for the NiFd-CN species using a single C/N/O scattering pathway. Number of scatters (n), interatomic distances (R) and Debye Waller factors (σ^2), delta energy (**Δ**E_o), R-factor, and reduced χ^2 are as reported from final fit parameters. All fits were performed in k-space in a range from k = 2 – 12 Å^{-1}.

	Fit #1	Fit #2	Fit #3	Fit #4	Fit #5	Fit #6	Fit #7	Fit #8	Fit #9
Ni – C/N/O Shell									
n	1	2	1	1	1	1	2	1	1
r (Å)	1.75	1.78	1.80	1.76	1.77	1.75	1.87	1.76	1.78
σ^2 (Å^2)	0.0104	0.00912	0.01306	0.00686	0.00386	0.01030	0.02196	0.0115	0.01574
Ni – C/N/O Shell									
n	-	-	1	1	-	-		-	2.12
r (Å)	-	-	2.12	2.12	-	-		-	0.01574
σ^2 (Å^2)	-	-	0.00019	0.0000	-	-		-	
Ni – S Shell									
n	3	3	3	3	3	3	3	3	3
r (Å)	2.30	2.30	2.31	2.34	2.31	2.29	2.29	2.30	2.33
σ^2 (Å^2)	0.00615	0.00615	0.00821	0.00781	0.00662	0.00579	0.00574	0.00610	0.0076
Ni – Fe Shell									
n	3	3	3	3	3	3	3	3	3
r (Å)	2.73	2.73	2.74	2.75	2.73	2.73	2.73	2.73	2.75
σ^2 (Å^2)	0.00722	0.00722	0.00719	0.00576	0.00608	0.00818	0.00826	0.00690	0.00651
Ni – O Shell									
n	1	1	1	1	1	1	1	-	-
r (Å)	2.96	3.01	3.01	3.00	2.93	2.97	2.84	-	-
σ^2 (Å^2)	0.0144	0.01420	0.01420	0.00940	0.02025	0.01400	0.1176	-	-
Ni – CO Shell									
n	2	2	2	2	2	2	2	-	-
r (Å)	3.01	2.98	2.99	3.05	3.00	3.01	2.94	-	-
σ^2 (Å^2)	0.00023	0.00087	0.00087	0.00031	0.00030	0.00025	0.0000	-	-
% contribution	60	60	60	50	50	70	70	60	60
ΔE_o	3.382	3.446	4.525	5.123	3.379	3.328	2.92	3.752	5.900
R – factor	0.049	0.100	0.035	0.039	0.059	0.051	0.076	0.065	0.044
Reduced χ^2	1239980	1373529	1818251	1006638	1151856	1068077	1233354	1226881	1044580

Table S6. Ni K-edge EXAFS fits for the NiFd-CO species using a single C/N/O scattering pathway. Number of scatters (n), interatomic distances (R) and Debye Waller factors (σ^2), delta energy (**Δ**E_o), R-factor, and reduced χ^2 are as reported from final fit parameters. All fits were performed in k-space in a range from k = 2 – 12 Å^{-1}.

Discussion of Ni K-edge EXAFS fitting

The fits to the Ni K-edge EXAFS spectrum for the $[NiFe_3S_4]^{2+}$ and $[NiFe_3S_4]^{+}$ Fd were more straightforward than those for the $[NiFe_3S_4]$-CN and $[NiFe_3S_4]^{+}$-CO Fd, so we will first discuss how those EXAFS fits were obtained. All fits were obtained using the Artemis software provided within the Demeter package (Version 0.9.26)[5] using a k-range of 2-12 $Å^{-1}$. The best fit to the data for the $[NiFe_3S_4]^{2+}$ Fd includes a single O scattering pathway, three identical S scattering pathways, and three identical Fe scattering pathways with distances of 2.01, 2.21, and 2.64 Å, respectively (**Table S1**). The DFT-optimized structures suggest that there would be two short Ni-S bonds and one long Ni-S bond, thus making the three sulfurs inequivalent. Therefore, we constructed a model that included inequivalent S and Fe scattering pathways (Fit 3). This fit reduces the R-factor; however, it increases the reduced χ^2. Therefore, in the absence of additional data, we chose to reduce the number of variables by making the S and Fe scattering pathways equivalent to obtain a better statistical model. Additionally, we sought to determine if the O scattering pathway, likely belonging to the aspartate residue, was necessary to include in the fit. By removing the O scattering pathway, the results of the fit were dependent on whether the system was modelled with three equivalent S atoms. For the model containing three equivalent S scattering pathways, the R-factor and reduced χ^2 both increased while also predicting an unreasonable $\mathbf{\Delta}E_o$ (Fit 2). For the model containing two equivalent S scattering pathways and one inequivalent pathway, the R-factor does not change significantly; however, the reduced χ^2 increases, and the predicted $\mathbf{\Delta}E_o$ is also unreasonably large (Fit 4). Therefore, we suggest that the aspartate residue is likely bound to the nickel center in the $[NiFe_3S_4]^{2+}$ Fd.

We performed a similar analysis on the Ni K-edge EXAFS data collected for the $[NiFe_3S_4]^{+}$ Fd. The best fit to the data includes a single O scattering pathway, three identical S scattering pathways, and three identical Fe scattering pathways with distances of 1.95, 2.27, 2.65 Å, respectively (**Table S2**). Once again, the DFT-optimized models suggest two long Ni-S bonds, and one short Ni-S bond, so we used a model including two equivalent S scattering atoms, and one inequivalent S scattering pathway. This time, the R-factor and reduced χ^2 decreased; however, the decrease in the R-factor was much smaller than in the $[NiFe_3S_4]^{2+}$ model (Fit 3). Additionally, the distances that this fit predicts are nearly identical for the different scattering pathways. We concluded that an acceptable model could be obtained without the inclusion of additional variables, leading to the selected model containing three equivalent S and Fe scattering pathways. Like the $[NiFe_3S_4]^{2+}$ fit, removal of the O scattering pathway significantly increases both the R-factor and reduced χ^2 (Fits 2/4), suggesting that this pathway is necessary to include and that the aspartate remains bound to the nickel center upon reduction.

The $[NiFe_3S_4]$-CN models become slightly more complicated due to the identity of an additional possible scattering pathway from binding CN^-. The best fit to the data includes a single C scattering pathway, a single O scattering pathway, two S scattering pathways, and three Fe scattering pathways with distances of 1.88. 1.87, 2.29, and 2.72 Å, respectively (**Table S3**). Additional scattering pathways responsible for multiple scattering to the nitrogen of the CN^- ligand were included as well. Removal of the multiple scattering pathway significantly increased the R-factor and reduced χ^2 (Fits 7/8), so we decided to keep this pathway in the model for all additional fits. In comparison to the rest of the fits (Fits 2-6) the reported fit has the lowest R-factor and a comparably low reduced χ^2. However, a few trends emerge from the different models. First, lower R-factors are reported for 3-4 coordinate Ni centers than for the 5 coordinate models (Fits 3/4). Second, the distances reported for all the scattering pathways are very similar, regardless of the fit. Finally, the C and O distances are nearly identical in all but one fit, and within the 0.13 Å resolution obtained at k = 12 $Å^{-1}$. Thus, we decided to explore whether an acceptable fit could be obtained with a single C/N/O scattering pathway. Refitting the data using a single C/N/O pathway produces acceptable fits, with similar R-factor's and reduced χ^2 values (**Table S5**). Once again, fits including a 3-4 coordinate nickel center (Fits 1-3/5) have significantly lower R-factors and reduced χ^2 values than those with 5-coordinate geometry at the nickel (Fits 4/6). Removal of the multiple scattering pathway significantly increases the R-factor and reduced χ^2 (Fits 6-8). Overall, the Ni-S and Ni-Fe distances do not change significantly in comparison to the fits from **Table S3**, but the Ni-C/N/O distances are all slightly

shorter (~1.87 to 1.84 Å). From this data, it appears that combining the C and O scattering pathways into a single C/N/O scattering pathway produces acceptable models of the experimental data.

Modeling the $[NiFe_3S_4]^+$-CO Fd EXAFS traces is more complicated because the $[NiFe_3S_4]^+$-CO Fd sample contains residual $[NiFe_3S_4]^+$ Fd due to the low binding affinity for CO. These fits were carried out including previously reported parameters for the $[NiFe_3S_4]^+$ Fd model while modifying the contributing amount, as given by the parameter "% contribution" in **Table S4**. The best fit to the data includes a single C scattering pathway, three S scattering pathways, and three Fe scattering pathways with distances of 1.80, 2.29, and 2.73 Å, respectively. Additional scattering pathways responsible for the multiple scattering to the O of the CO ligand were included as well. Similar to the $[NiFe_3S_4]$-CN fits, removal of the multiple scattering pathway generally increased the R-factor and χ^2 (Fits 7-9). By adding an additional primary sphere O scattering pathway to make the nickel center 5-coordinate, the R-factor and χ^2 both decrease (Fit 2). However, the best-fit distance of 2.14 Å for the Ni-O bond is substantially longer than would be chemically expected, leading us to reject that model. Changing the "% contribution" from 60:40 $[NiFe_3S_4]^+$-CO:$[NiFe_3S_4]^+$ to 50:50 or 70:30 (Fits 3-6) produced insignificant changes in bond distances and the resulting R-factors and reduced χ^2 values such that we cannot definitively constrain those parameters beyond these bounds. However, the XANES data suggest that a lower bound of 50:50 is a reasonable assumption. Similar to the fits of the $[NiFe_3S_4]$-CN species, we sought to determine if a better model is obtained by combining the C and O scattering pathways into a single C/N/O pathway. A variety of models were generated using this combined scattering pathway (**Table S6**). By using a single C/N/O pathway, we were able to obtain a reasonable R-factor and reduced χ^2 for direct comparison to our reported fit (Fit 1). The Ni-S and Ni-Fe distances remain unchanged; however, the Ni-C/N/O distance is significantly shorter (1.80 to 1.75 Å). This distance is unreasonably short for a Ni-C bond, preventing us from considering this a viable model of the data. Introduction of an additional atom to this scattering pathway significantly increases the R-factor and reduced χ^2, while also predicting unreasonably short Ni-C/N/O bonds (Fit 2). Breaking this into two inequivalent C/N/O scattering pathways produces similar results as reported in **Table S4**, but breaking the degeneracy of this scattering pathway is essentially the same as modeling with a single C and O pathway, thus suggesting that this would be a less satisfactory model of the data (Fits 3-4). Overall, the general increase in R-factor and reduced χ^2 values and the unreasonably short Ni-C/N/O distances obtained with this alternative model indicate that the data is best reproduced including a single C scattering pathway and a single O scattering pathway, as represented in **Table S4**.

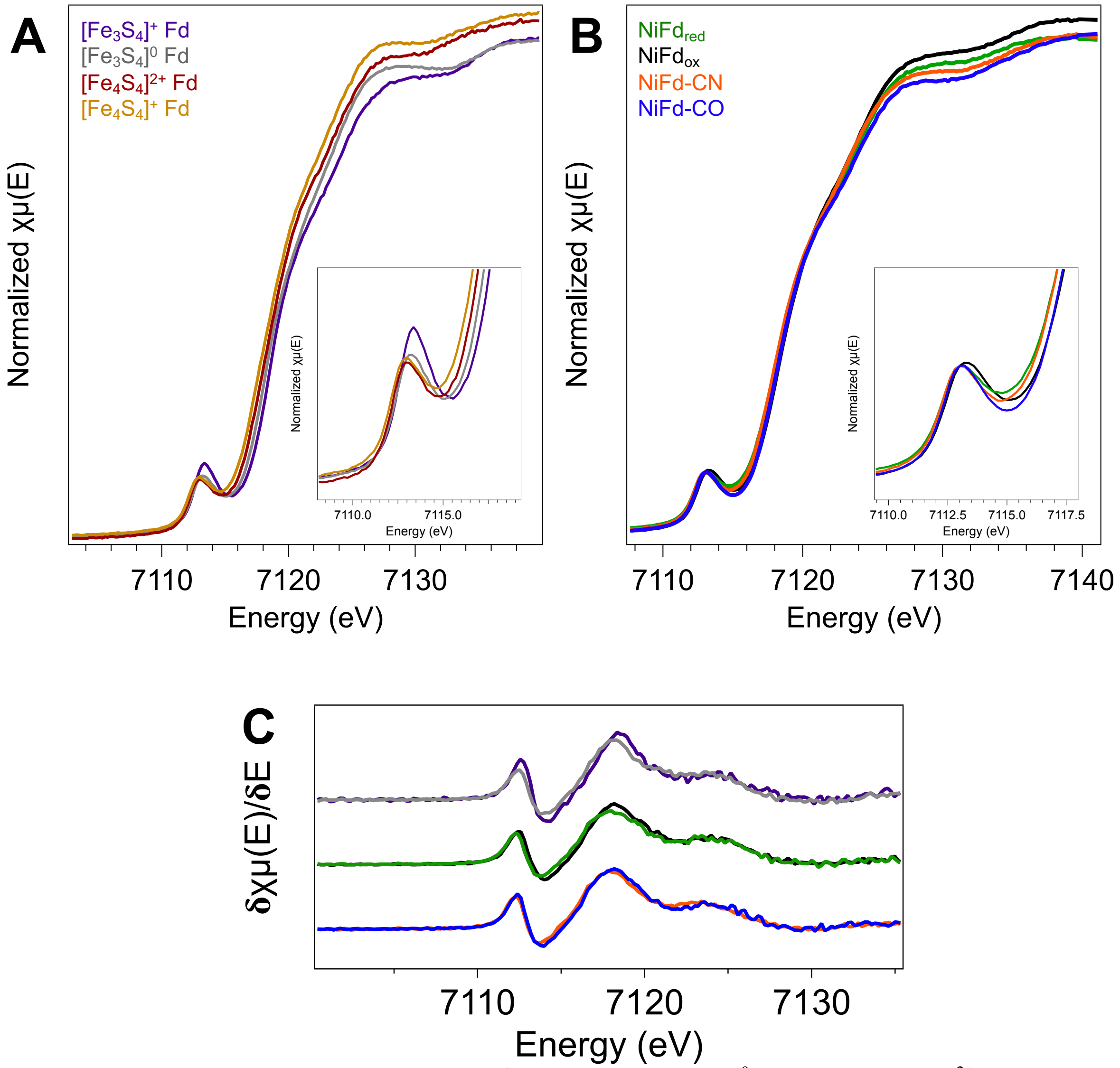

Figure S24. (**A**) Fe K-edge XANES of $[Fe_3S_4]^+$ Fd (*purple)*, $[Fe_3S_4]^0$ Fd (*gray*), $[Fe_4S_4]^{2+}$ Fd (*brown*), $[Fe_4S_4]^+$ Fd (*yellow*). (*Inset)* Zoomed-in view of the pre-edge region. (**B**) Fe K-edge XANES of the four isolated forms of NiFd. (*Inset)* Zoomed-in view of the pre-edge region. (**C**) Fe K-edge first-derivative spectra of $[Fe_3S_4]^+$ (*purple*), $[Fe_3S_4]^0$ Fd (*gray*), $NiFd_{red}$ (*green*), $NiFd_{ox}$ (*black*), NiFd-CN (*orange*), and NiFd-CO (*blue)*.

Distances (Å) and Angles (°)	$[NiFe_3S_4]^+$	$[NiFe_3S_4]^{2+}$
Ni-O12	1.959	1.912
Ni-S4	2.349	2.356
Ni-S5	2.307	2.331
Ni-S7	2.356	2.307
Ni-Fe3	2.570	2.616
Ni-Fe1	2.667	2.621
Ni-Fe2	2.670	2.689
∠(S4-Ni-S5)	112.56	109.45
∠(S4-Ni-S7)	106.23	106.44
∠(S5-Ni-S7)	108.33	108.44
∠(S4-Ni-O12)	118.64	114.03
∠(S5-Ni-O12)	112.47	108.56
∠(S7-Ni-O12)	96.69	109.79

Table S7. DFT-optimized geometric parameters of $NiFd_{red}$ and $NiFd_{ox}$ models.

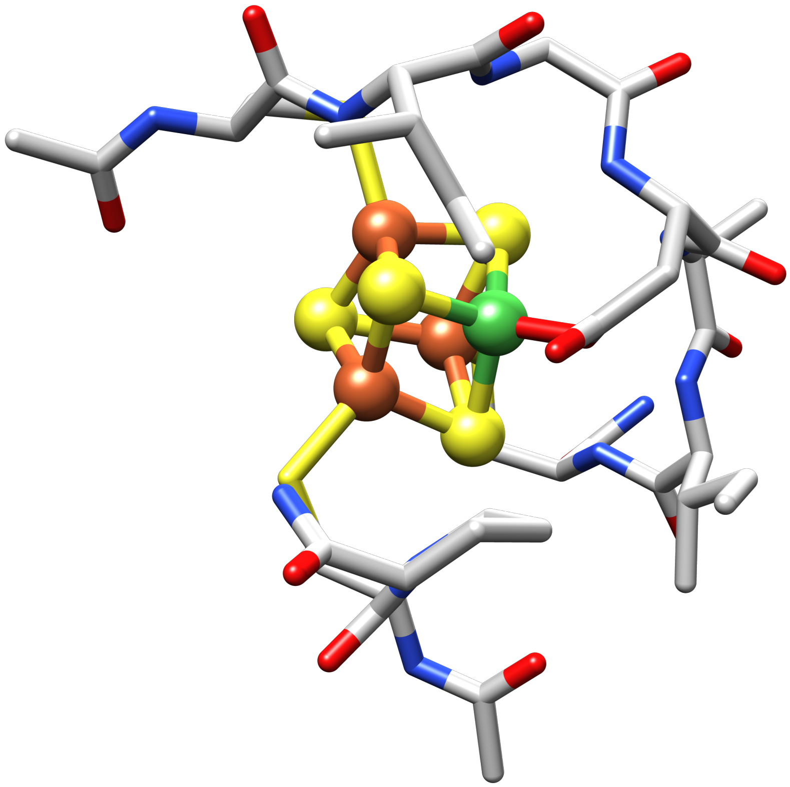

Figure S25. High-spin DFT geometry-optimized structure of the $NiFd_{ox}$ model.

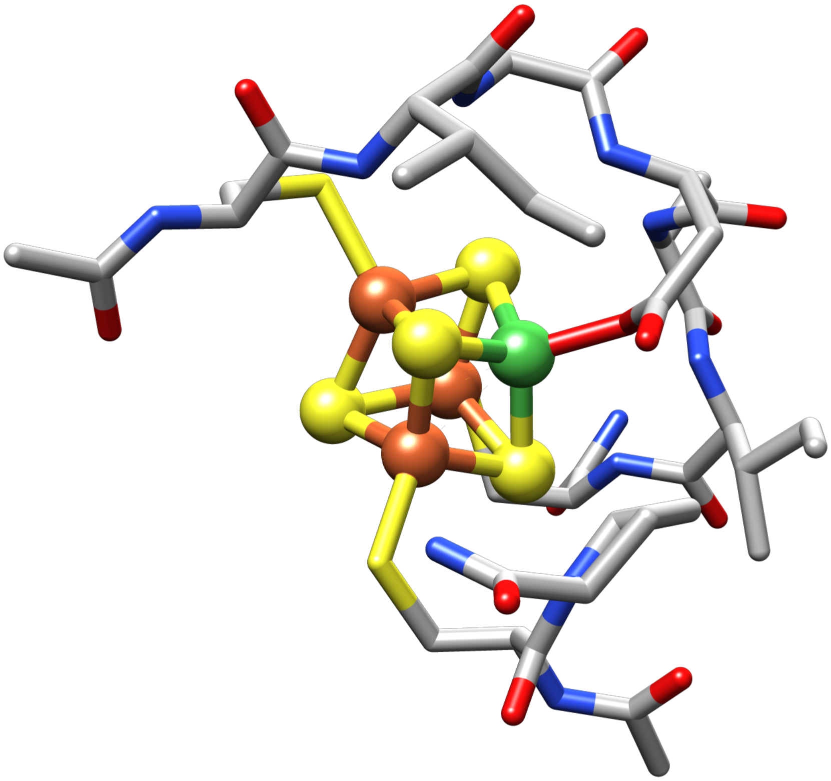

Figure S26. High-spin DFT geometry-optimized structure of the $NiFd_{red}$ model.

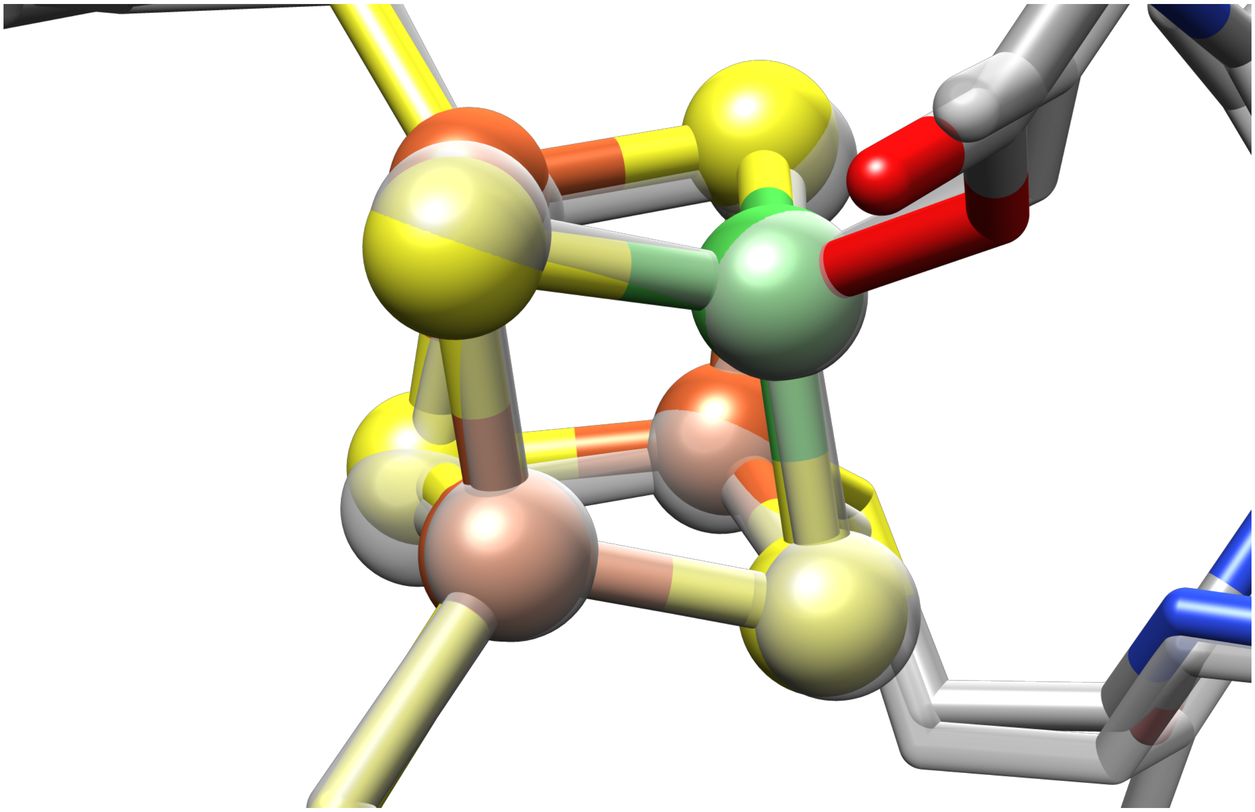

Figure S27. High-spin DFT geometry-optimized structures of the $NiFd_{red}$ (*grey*) and $NiFd_{ox}$ (*color*) models overlaid for comparison.

Distances (Å) and Angles (°)	$[NiFe_3S_4]^+$-CO	$[NiFe_3S_4]^+$-CO
	A	B
Ni-C40	1.840	1.805
Ni-O12	2.154	---
Ni-S4	2.601	2.322
Ni-S5	2.369	2.314
Ni-S7	2.393	2.320
Ni-Fe1	2.865	2.635
Ni-Fe2	2.769	2.521
Ni-Fe3	2.900	2.604
∠(C40-Ni-O12)	89.32	---
∠(S4-Ni-S5)	97.66	107.69
∠(S4-Ni-S7)	103.53	113.67
∠(S5-Ni-S7)	100.67	106.47
∠(S4-Ni-O12)	160.26	---
∠(S5-Ni-O12)	92.06	---
∠(S7-Ni-O12)	91.41	---

Table S8. DFT-optimized geometric parameters of the (**A**) D14-bound and (**B**) D14-unbound NiFd-CO models.

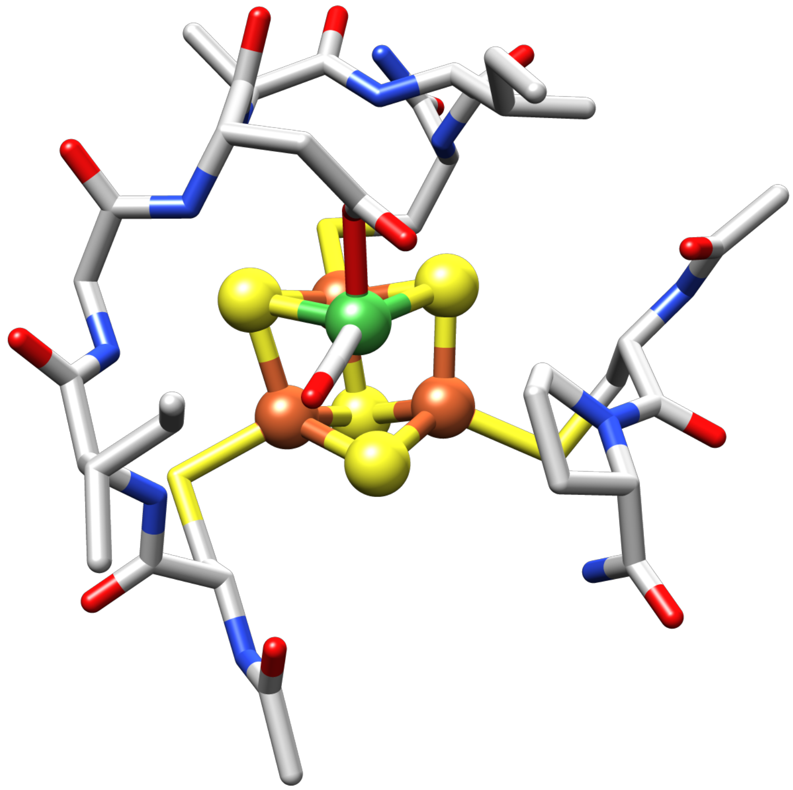

Figure S28. High-spin DFT geometry-optimized structure of the NiFd-CO model with ligation of aspartate (D14) residue.

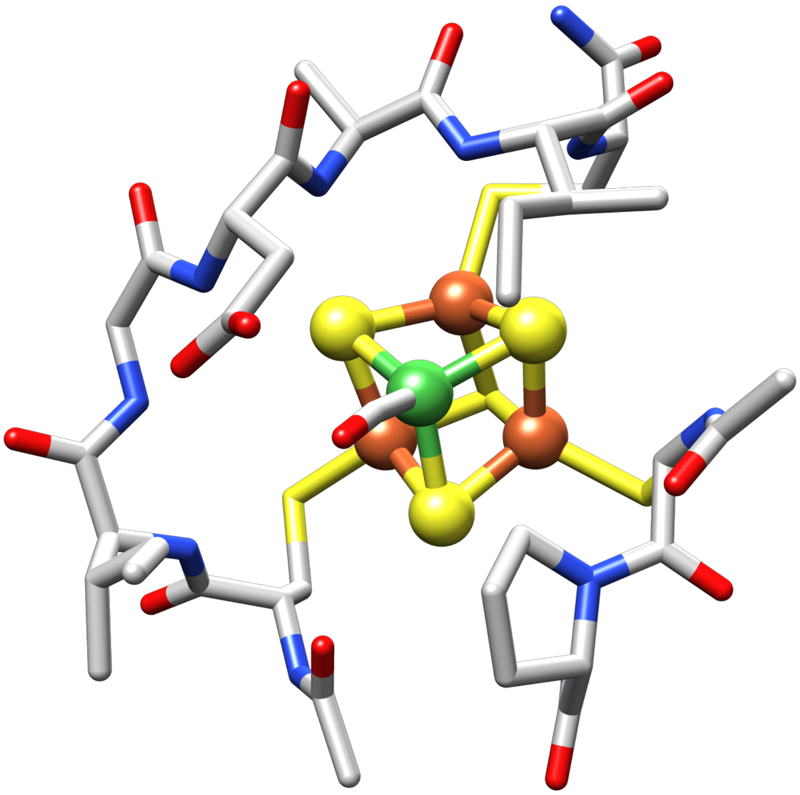

Figure S29. High-spin DFT geometry-optimized structure of the NiFd-CO model without ligation of aspartate (D14) residue.

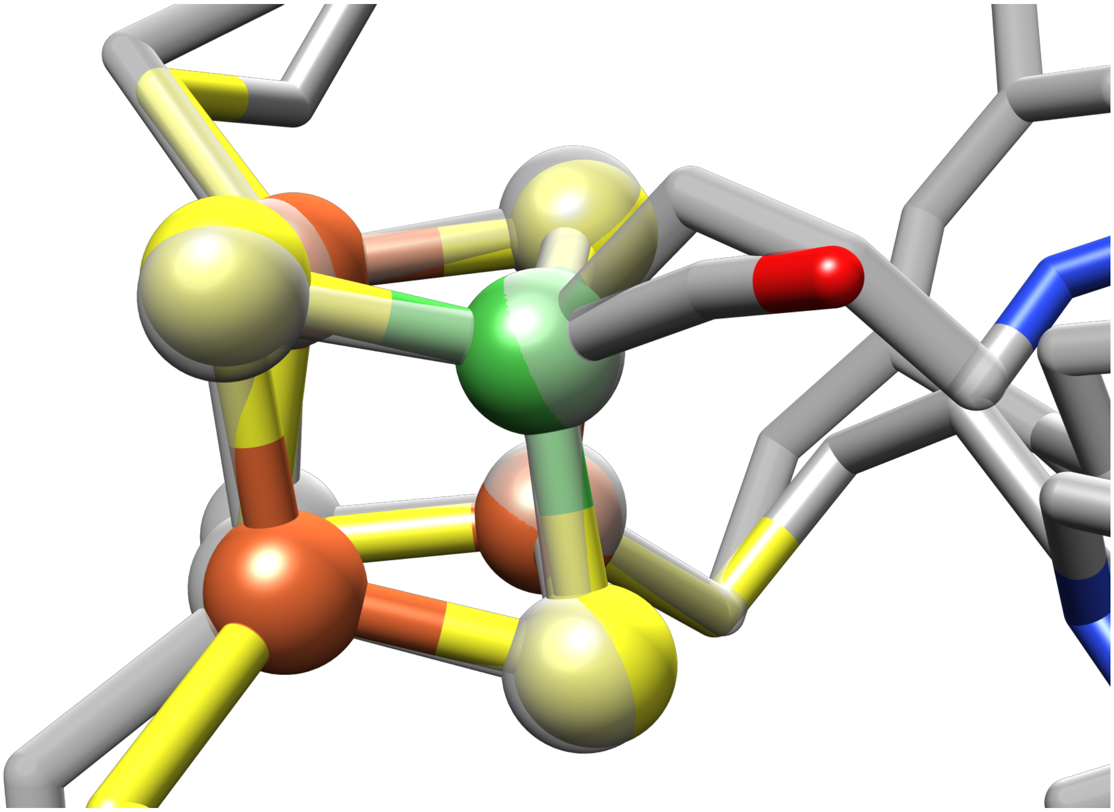

Figure S30. High-spin DFT geometry-optimized structures of the $NiFd_{red}$ (*gray*) and NiFd-CO (*color*) models overlaid for comparison.

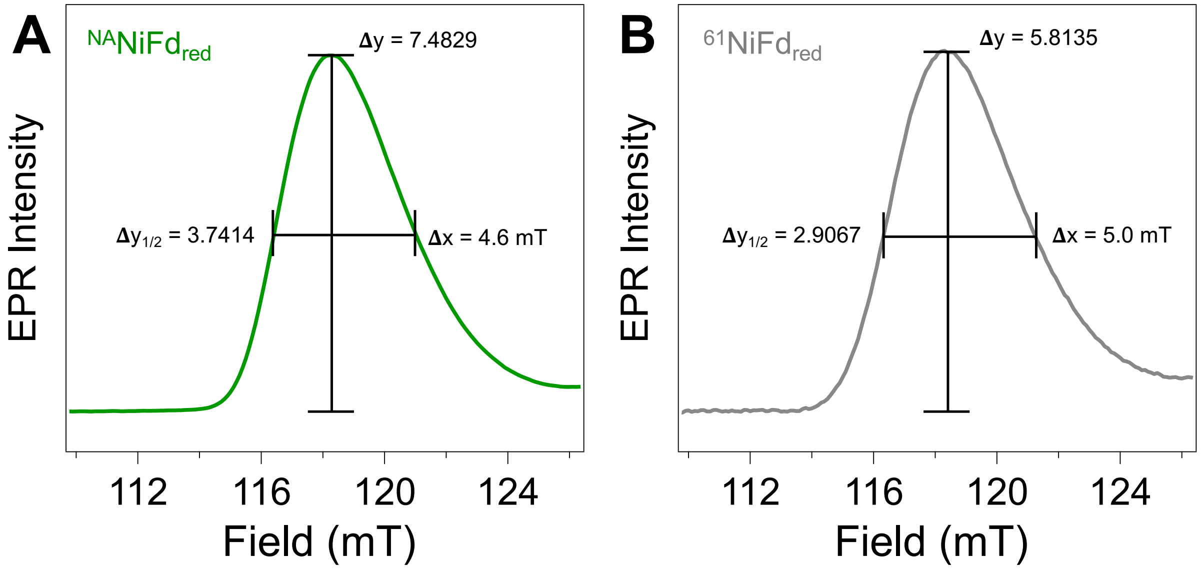

Figure S31. CW EPR spectra (ν = 9.37 GHz, T = 8 K, $P_{\mu w}$ = 20 mW) of (**A**) natural-abundance Ni (^{NA}Ni) (*green*) and (**B**) ^{61}Ni-labelled (*gray*) $NiFd_{red}$ samples. Vertical hashmarks indicate total EPR intensity of the g = 5.7 transition. Horizontal hashmarks are placed at half the maximum of the EPR intensity value spanning the width of the EPR transition (FWHM).

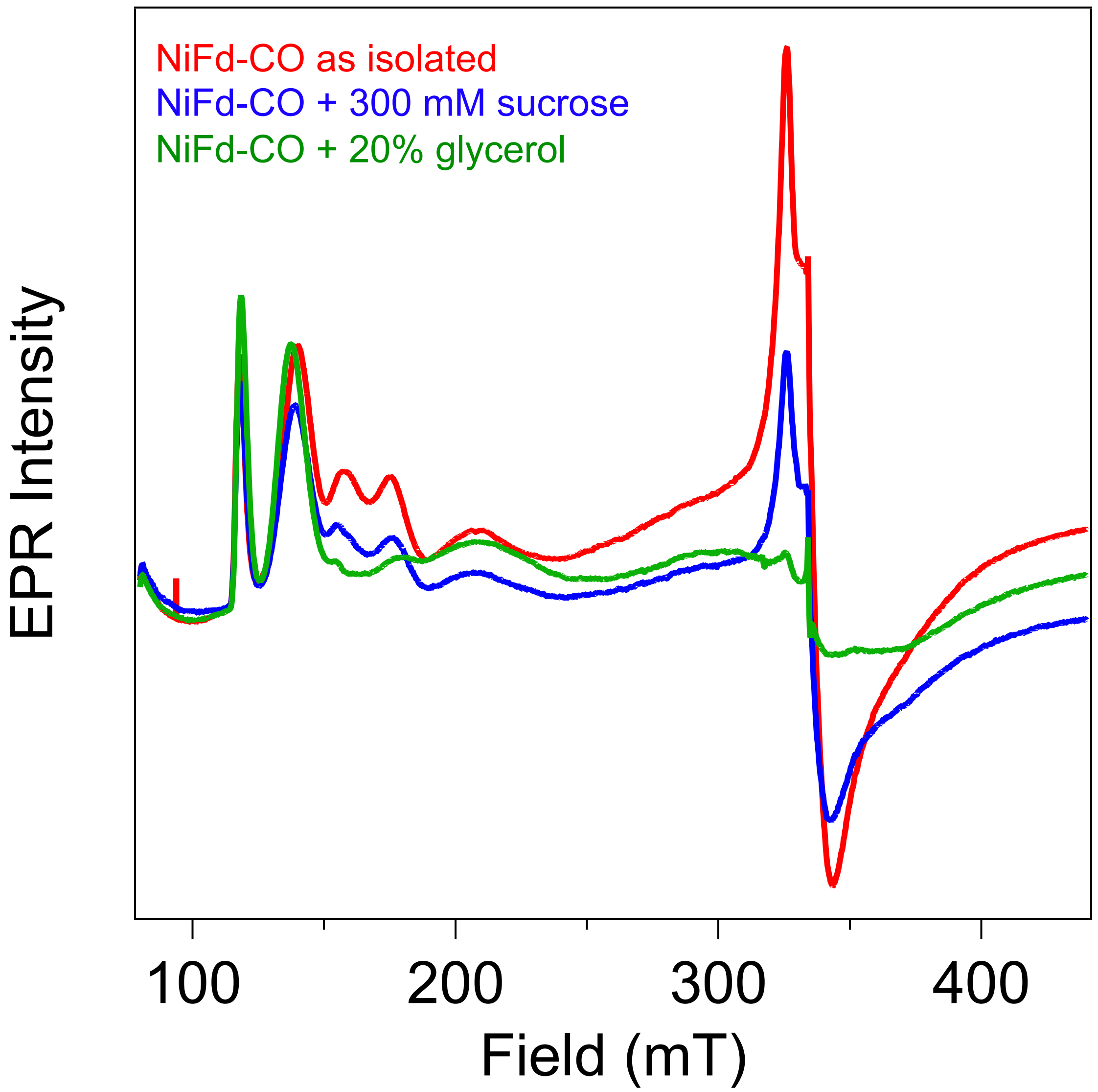

Figure S32. CW-EPR spectra (ν = 9.37 GHz, T = 8 K, $P_{\mu w}$ = 20 mW) of NiFd-CO as isolated (*red*), with 300 mM sucrose (*blue*), and 20 % glycerol (*green*) added as a glassing agent.

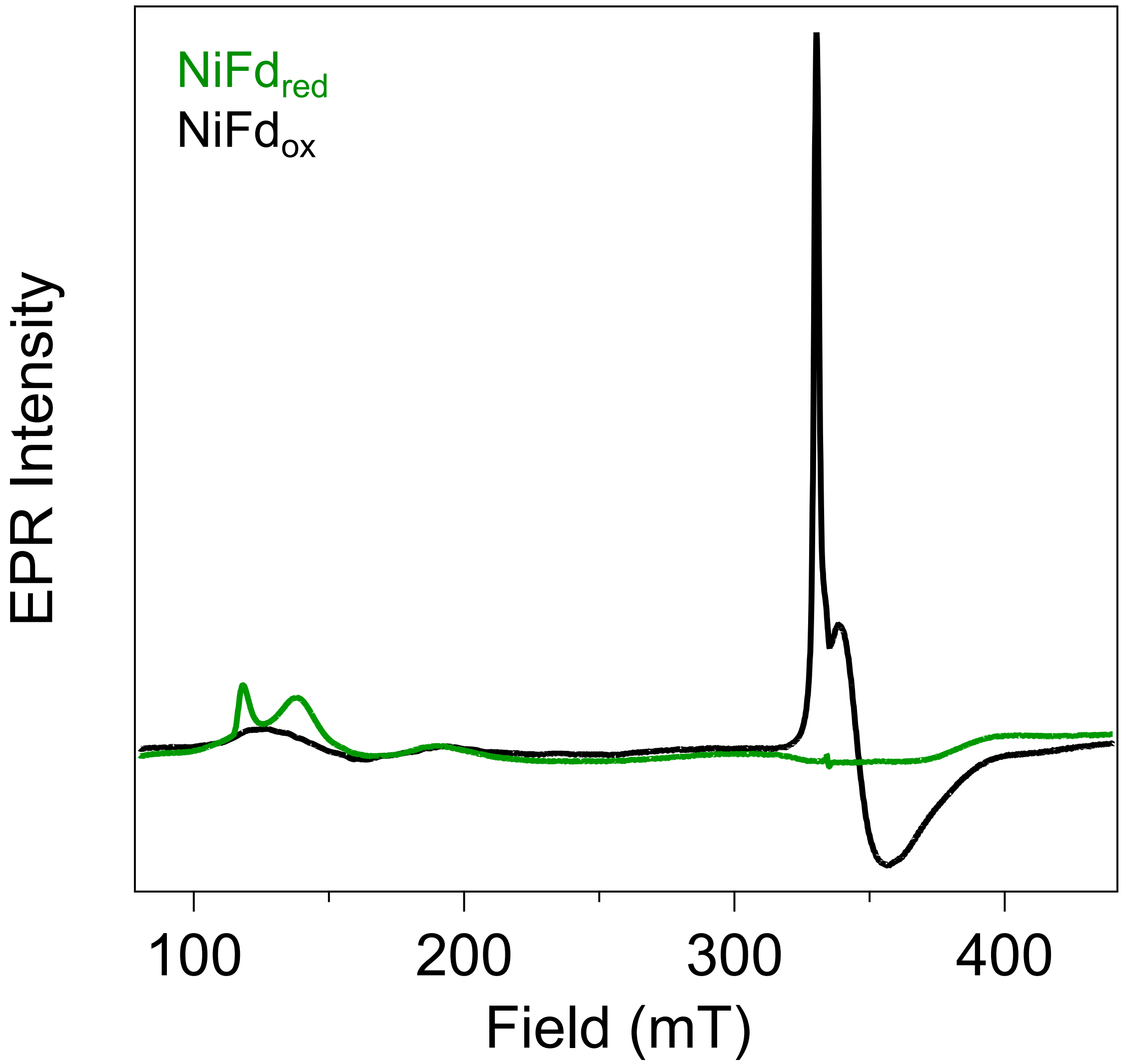

Figure S33. CW-EPR spectra (ν = 9.37 GHz, T = 8 K, $P_{\mu w}$ = 20 mW) of XAS samples of NiFd$_{red}$ (*green*) and NiFd$_{ox}$ (*black*). NiFd$_{red}$ and NiFd$_{ox}$ were supplemented with 300 mM sucrose and 20 % glycerol, respectively, as glassing agents.

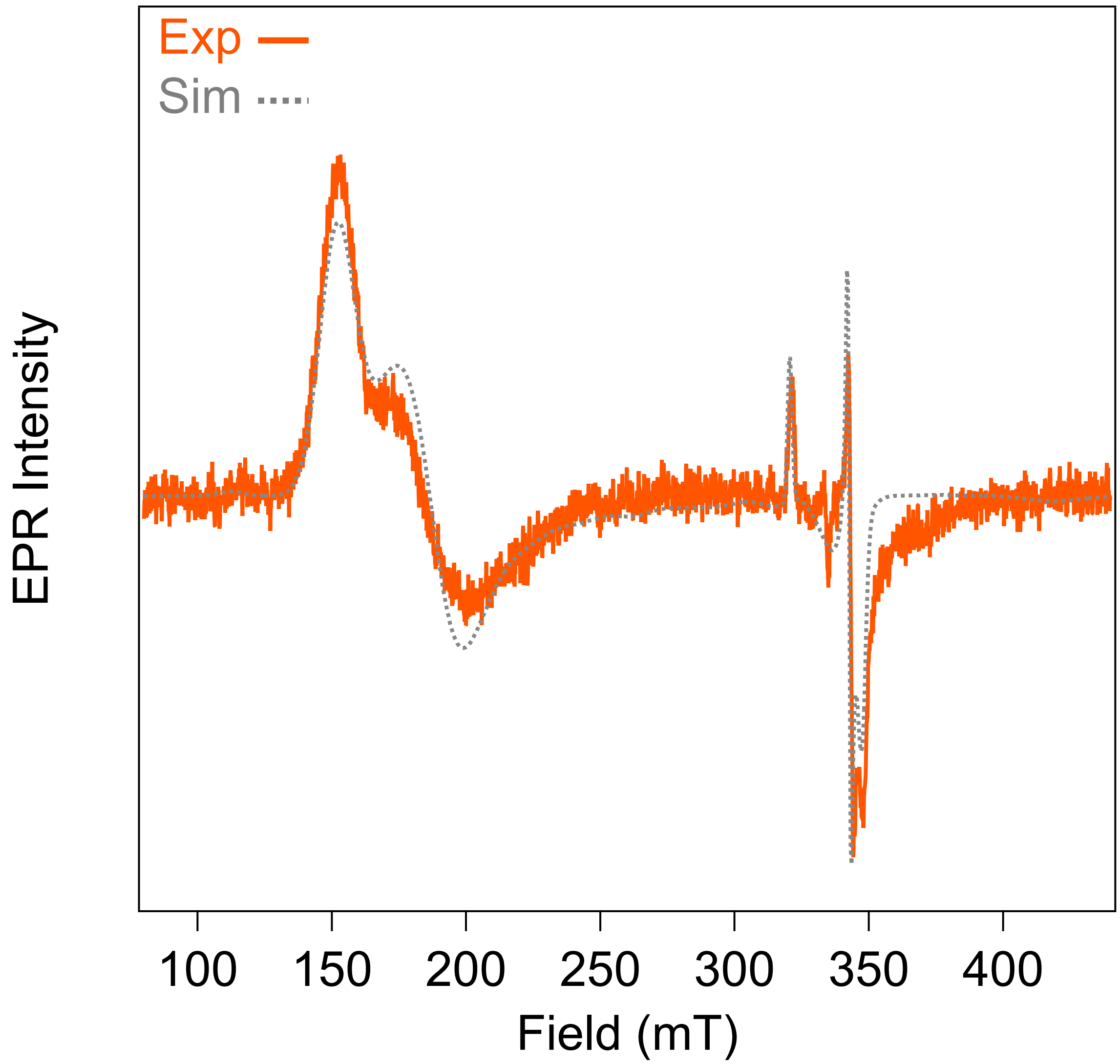

Figure S34. CW-EPR Spectra (n = 9.37 GHz, T = 10.5 K, $P_{\mu w}$ = 2 µW) of NiFd-CN experimental (*orange*) and simulated (*gray*). Simulation parameters: $[Fe_4S_4]$-CN S = ½ , g = [2.089 1.953 1.926], 4% contribution, NiFd-CN S = 3/2, g_{iso} = 2.0, E/D = 0.07, 96% contribution

Appendix S1. Example ORCA Input File

```
# JS051022_2z8q_NiFe3S4_WT_Fd_MR_Hopt_HS.inp
# Hydrogen optimization of NiFe3S4_Fd_MR Wild Type mutant from 2z8q
# calculation parameters
! UKS TPSSh LooseSCF Grid4 GridX4 NormalPrint PrintBasis UNO UCO
! RIJCOSX ZORA-def2-TZVP(-f) def2/J DecontractAux ZORA D3 SlowConv
! OPT

#parallel
%maxcore 7500
%pal
 nprocs 28
end

%geom Constraints #Lock Alpha Carbons
{ C 69 C } #Cys
{ C 92 C } #Asp
{ C 112 C } #Cys
{ C 53 C } #Cys
end
end

%scf
  maxiter 3000
AutoStart true
end


* xyz #charge# -3  #spin#  16
  C   -14.68081578563548     41.41963326250767    -13.22630309634737
  O   -14.02813480928998     42.42037235041950    -12.95647861123537
  C   -14.62511292611868     38.80259199589004    -10.65007982250591
  O   -15.60443917918184     38.20077630325205    -11.11505298359716
  S   -14.82900573789319     41.12066094169669     -8.33970159039862
  C   -14.21575069287523     36.84706124581985     -7.82594916628730
  O   -14.37441588012966     35.76376862524248     -7.26270230793884
  C   -13.66409763475487     37.61979640360165     -4.81498128497526
  O   -13.98470734824216     37.12259589902028     -3.73781323871707
  C   -10.95214283671992     38.43982996819009     -3.20477964541398
  O   -10.24920551749452     38.06885514269839     -2.26125364716090
  C   -10.09404693932918     41.30487772318711     -2.00462880517038
  O   -9.92398412331721     42.05877045450885     -1.03744857983322
  C   -7.63461715917463     43.03113802976073     -2.80459870572071
  O   -6.73675847794458     43.63009425165561     -2.21165493373874
  C   -9.09928654534324     45.85929569835658     -2.57430556651435
  O   -8.99747065799455     47.08572321309484     -2.52583728837861
  S   -11.10555383604692     44.79416267939541     -4.94201883082835
  C   -4.12531468717952     42.32126009849461     -7.68996363108924
  O   -4.09875544943810     41.12632374909916     -7.41224924049372
  C   -5.63233772293404     40.92968093988387     -9.96379131936040
  O   -4.89326991659135     41.09447548296701    -10.93589864921515
  S   -8.38752070906200     42.27673870753855    -10.76818981634515
  N   -6.13221932196337     39.72021913542179     -9.62036979956439
  C   -6.42835519781713     38.43880003310423    -11.77463823218626
  O   -6.00828810737756     37.62936147531722    -12.60430941194667
```

Fe	-11.06080949054705	43.17568990729951	-6.57575258715039
Fe	-12.56281314397591	41.60095511538679	-8.15736781474417
Fe	-9.85661108733898	42.08103083542508	-8.98579840512689
S	-11.02515725894759	40.06493999924839	-9.13777464330459
S	-8.86720916271279	42.34313653304635	-6.88379617469762
S	-11.59309735746299	43.63790171071501	-8.83478459366786
S	-12.32757751334238	41.38725131298754	-5.83138365496679
C	-15.35942434041022	41.27252138957590	-14.58525235583655
H	-16.05206297733888	42.10642760196448	-14.71803431399016
H	-14.59534001298656	41.34574175581009	-15.36155568702603
H	-15.90107766277187	40.33092219819493	-14.70174655505417
N	-9.66805658222644	45.12595231025678	-1.60044209596199
H	-9.71587790032618	44.11330517383340	-1.62198837013415
H	-10.02074158151595	45.61315865479182	-0.79440541610870
C	-3.15747152573591	43.30088575903967	-7.04640492323258
H	-3.42751302976727	43.40432419968859	-5.99296463283041
H	-2.15100748048542	42.88383462989719	-7.09933890331443
H	-3.16828683936889	44.28872152543271	-7.51299542640255
N	-7.51763952145840	39.20647240786797	-11.95970027105644
H	-7.94782269939599	39.18786807137477	-12.87014994755651
H	-7.73948681731429	39.99506739975489	-11.35064532628120
C	-15.23770043328332	41.20311265592890	-10.12043338026750
H	-16.29074149157672	40.93503448279267	-10.23831594917631
H	-15.08712346153817	42.22087890002832	-10.47995535241941
C	-6.90692148100992	43.07377486307082	-10.04890205105931
H	-7.21724821348138	43.94758158941971	-9.46981048057415
H	-6.26283051002195	43.38523603778865	-10.87475299165645
C	-6.12800000122362	42.12900206738498	-9.14099998461580
H	-6.79220064358894	41.81131539069102	-8.33809312328984
C	-6.69412618151485	39.34339552148547	-8.30419650781632
H	-6.37040338250388	40.04457933353627	-7.54249068760723
H	-7.78326906744142	39.30978920656383	-8.34035306352116
C	-6.11679767360765	37.94742752917149	-8.09934771700963
H	-6.69938267575373	37.37933991875926	-7.37875442793568
H	-5.07731912237173	38.02790047959019	-7.76557929638698
C	-6.18761364662019	37.35778321790408	-9.50512781919100
H	-5.54711666540331	36.49025367670906	-9.66731492966038
H	-7.21900906270548	37.07285411355294	-9.72650749538973
C	-5.76700000120478	38.53499994213040	-10.40300001417404
H	-4.68948617985084	38.53853670805125	-10.58502019962941
C	-9.39088873739867	45.44726259958075	-5.03967037935082
H	-8.89379599216169	45.03987883711589	-5.92064285136214
H	-9.42722068163742	46.53437489469779	-5.10870628309005
C	-8.57199999520964	45.08999998535238	-3.79699999280457
H	-7.54450412732127	45.44131057411877	-3.92829291476434
C	-7.75500005851925	41.49599799950864	-2.67599999642150
H	-7.44754172749349	41.27626187299469	-1.65131576051157
C	-6.86240447785269	40.74803807605490	-3.69401339853657
H	-7.39605812292832	40.77962524093056	-4.65219793093777
C	-5.49971733797639	41.40545585326007	-3.90104883085750
H	-4.94956411467833	41.47546315160628	-2.95796238654581
H	-4.91143523035857	40.83079905599725	-4.61842728349585
H	-5.60811574896068	42.41609721294995	-4.29644741186908
C	-6.73279467653330	39.28006032513701	-3.25615658824708
H	-7.70599319660027	38.91730666165713	-2.91290078621176
H	-6.06042737357711	39.23826988562482	-2.38831657942495

C	-6.22554590025370	38.35877501339592	-4.36219344125746
H	-6.16682616261018	37.32464364896485	-4.00901846208128
H	-6.90304354203730	38.38078799604469	-5.21689533841483
H	-5.23383025167165	38.64957929657785	-4.71788077884462
C	-11.47299993917475	40.70399900457692	-2.28900002619045
H	-12.04315912273927	41.53071988794060	-2.72570806293891
C	-12.14528481184246	40.25761503095866	-0.98970532659016
H	-11.62541771855929	39.38961066997646	-0.58411867302470
H	-12.11057651743669	41.07169708757643	-0.26556838314455
H	-13.18165599919553	39.98435594416587	-1.19609208482156
C	-11.29599995163482	37.42999999478824	-4.32699993917534
H	-11.68196464454248	36.58321742576738	-3.75338314308597
C	-14.73100004139307	38.08399999868007	-5.80000003785376
H	-14.97626911521134	39.12344206784034	-5.57137113938362
H	-15.60300914676288	37.45302576687694	-5.62258691214732
C	-13.88219923914148	36.89148759918269	-9.32253589273865
H	-14.76529633494563	36.50203695641580	-9.83394231859355
C	-12.68013069860923	35.97804576562008	-9.66357602335127
H	-12.97900000565825	34.95999900865609	-9.37900000293749
C	-12.42817786245039	36.00815096346758	-11.17284498243287
H	-11.69359935946156	35.25200568741518	-11.45884324344710
H	-13.35149963425148	35.81898262826779	-11.72920972760335
H	-12.04319120411994	36.98376046103285	-11.47955835471813
C	-11.43117292774405	36.34019205423368	-8.85443727040694
H	-11.69806740910967	36.34791446495642	-7.79404736717309
H	-11.09914926672399	37.35408173243529	-9.09735323071830
C	-10.26762760697716	35.37239810231957	-9.05662534828591
H	-9.88976633698792	35.40326900229043	-10.08258930595434
H	-9.45014566053223	35.64856202162871	-8.38979967705260
H	-10.57260210142186	34.34114807765846	-8.83949236366020
C	-14.40300000460373	40.27299899653649	-11.01500000572891
H	-13.34772396403510	40.54604766203713	-10.94475736573594
N	-8.53738711230449	43.64788299165041	-3.59926284879340
H	-9.26339564111890	43.11146602895450	-4.06472991143364
N	-9.11908170771935	41.02652284103566	-2.88143393174653
H	-9.32939427333903	40.42915968152636	-3.67643629119651
N	-11.50353154267161	39.66319393262195	-3.30499249216840
H	-11.89825335504513	39.94067432225501	-4.20580340081879
N	-12.38116190752722	37.81878793586649	-5.19768856216902
H	-12.19595544561612	38.36715026487659	-6.02886682602632
N	-14.32778267879129	38.03443199738285	-7.19202600902114
H	-14.34830907372563	38.91261867675021	-7.72270325697711
N	-13.72312114650344	38.24685025659083	-9.82683181640488
H	-12.92006424351522	38.81350394446002	-9.53361648105842
N	-14.85736318874403	40.36520832958211	-12.40124451159551
H	-15.49633171702098	39.61885028340743	-12.64812289411804
N	-5.00255272799746	42.86416671997715	-8.56216249560836
H	-5.00307176881433	43.86434987350270	-8.68336966982880
Ni	-10.39776531625226	40.61862323773784	-6.94264502818186
C	-10.05795149492108	36.96584770193966	-5.08194483275327
H	-10.26122980314238	36.03497096826651	-5.61387869876227
H	-9.27554722584312	36.76955859152556	-4.34278400491674
O	-9.78268143412115	39.21616754826236	-5.72101376891100
C	-9.53993367397923	37.99661241767713	-6.07770954937264
O	-8.95769292334009	37.62208869450964	-7.09463230322690

*

Supplemental References

(1) Lewis, L. C.; Shafaat, H. S. Reversible Electron Transfer and Substrate Binding Support [$NiFe_3S_4$] Ferredoxin as a Protein-Based Model for [NiFe] Carbon Monoxide Dehydrogenase. *Inorg. Chem.* **2021**, *60* (18), 13869–13875. https://doi.org/10.1021/acs.inorgchem.1c01323.

(2) Telser, J.; Lee, H.-I.; Smith, E. T.; Huang, H.; Brereton, P.; Adams, M. W. W.; Conover, R. C.; Johnson, M. K.; Hoffman, B. M. Investigation by EPR and ENDOR Spectroscopy of the Novel 4Fe Ferredoxin fromPyrococcus Furiosus. *Appl. Magn. Reson.* **1998**, *14* (2–3), 305–321. https://doi.org/10.1007/BF03161897.

(3) Slater, J. W.; Marguet, S. C.; Cirino, S. L.; Maugeri, P. T.; Shafaat, H. S. Experimental and DFT Investigations Reveal the Influence of the Outer Coordination Sphere on the Vibrational Spectra of Nickel-Substituted Rubredoxin, a Model Hydrogenase Enzyme. *Inorg. Chem.* **2017**, *56* (7), 3926–3938. https://doi.org/10.1021/acs.inorgchem.6b02934.

(4) McCreery, R. L. *Raman Spectroscopy for Chemical Analysis*, 1st ed.; John Wiley & Sons, Ltd, 2000. https://doi.org/10.1002/0471721646.

(5) Ravel, B.; Newville, M. *ATHENA* , *ARTEMIS* , *HEPHAESTUS* : Data Analysis for X-Ray Absorption Spectroscopy Using *IFEFFIT*. *J. Synchrotron Radiat.* **2005**, *12* (4), 537–541. https://doi.org/10.1107/S0909049505012719.

(6) Hummer, A. A.; Rompel, A. X-Ray Absorption Spectroscopy. In *Advances in Protein Chemistry and Structural Biology*; Elsevier, 2013; Vol. 93, pp 257–305. https://doi.org/10.1016/B978-0-12-416596-0.00008-7.

(7) Rehr, J. J.; Kas, J. J.; Prange, M. P.; Sorini, A. P.; Takimoto, Y.; Vila, F. Ab Initio Theory and Calculations of X-Ray Spectra. *Comptes Rendus Phys.* **2009**, *10* (6), 548–559. https://doi.org/10.1016/j.crhy.2008.08.004.

(8) Neese, F.; Wennmohs, F.; Becker, U.; Riplinger, C. The ORCA Quantum Chemistry Program Package. *J. Chem. Phys.* **2020**, *152* (22), 224108. https://doi.org/10.1063/5.0004608.

(9) Van Wüllen, C. Molecular Density Functional Calculations in the Regular Relativistic Approximation: Method, Application to Coinage Metal Diatomics, Hydrides, Fluorides and Chlorides, and Comparison with First-Order Relativistic Calculations. *J. Chem. Phys.* **1998**, *109* (2), 392–399. https://doi.org/10.1063/1.476576.

(10) Van Lenthe, E.; Ehlers, A.; Baerends, E.-J. Geometry Optimizations in the Zero Order Regular Approximation for Relativistic Effects. *J. Chem. Phys.* **1999**, *110* (18), 8943–8953. https://doi.org/10.1063/1.478813.

(11) Izsák, R.; Neese, F. An Overlap Fitted Chain of Spheres Exchange Method. *J. Chem. Phys.* **2011**, *135* (14), 144105. https://doi.org/10.1063/1.3646921.
